# Supplementary material for: Risks for death after admission to pediatric intensive care (PICU)—A comparison with the general population
Source: PLoS One. 2022 Oct 7;17(10):e0265792. doi: 10.1371/journal.pone.0265792 (PMC9543762; doi:10.1371/journal.pone.0265792)
Supplement: S1 Table — Anonymized. (PDF) [file pone.0265792.s001.pdf]

Suppl. Table S1. Complete dataset on the entire study population

Admission year	Age (years)	Follow up time (years)	Sex (Females=1)	PDRLast	Outcome (Deceased=1)	Single/repeated admission	Diagnostic group
2008	0	1.57E-01	0	1.29E+01	1	Repeated admissions	1
2008	9	1.23E+01	0	4.93E+00	0	Repeated admissions	1
2008	2	1.22E+01	0	9.13E+00	0	Single admission	1
2008	0	1.19E+01	0	3.05E+00	0	Single admission	1
2008	8	1.18E+01	0	1.11E+00	0	Single admission	1
2008	1	9.78E-01	0	5.90E+01	1	Repeated admissions	2
2008	0	1.24E+01	0	6.35E+00	0	Single admission	2
2008	0	1.21E+01	1	8.41E+00	0	Single admission	2
2008	0	1.20E+01	1	1.17E+00	0	Single admission	2
2008	0	1.19E+01	0	2.71E-01	0	Single admission	2
2008	0	1.18E+01	1	2.13E+00	0	Single admission	2
2008	0	1.18E+01	0	2.25E+00	0	Single admission	2
2008	0	1.16E+01	0	1.40E+00	0	Single admission	2
2008	0	1.15E+01	0	2.25E+00	0	Single admission	2
2008	0	1.15E+01	0	1.60E+01	0	Single admission	2
2008	0	1.15E+01	0	1.03E+01	0	Single admission	2
2008	0	1.15E+01	0	1.17E+00	0	Single admission	2
2008	0	1.24E+01	1	1.13E+00	0	Single admission	3
2008	6	1.24E+01	1	7.14E+00	0	Single admission	3
2008	2	1.23E+01	0	5.91E-01	0	Repeated admissions	3
2008	2	1.23E+01	0	1.16E+00	0	Repeated admissions	3
2008	2	1.23E+01	1	2.98E+00	0	Single admission	3
2008	0	1.22E+01	0	1.66E+00	0	Single admission	3
2008	2	1.22E+01	0	6.94E+00	0	Single admission	3
2008	5	1.22E+01	1	4.11E-01	0	Single admission	3
2008	1	1.20E+01	0	3.02E+00	0	Single admission	3
2008	18	4.82E+00	0	5.69E-01	1	Repeated admissions	3
2008	0	1.19E+01	0	2.02E+00	0	Single admission	3

Admission year	Age (years)	Follow up time (years)	Sex (Females=1)	PDRLast	Outcome (Deceased=1)	Single/repeated admission	Diagnostic group
2008	0	1.18E+01	0	2.80E+00	0	Single admission	3
2008	0	1.17E+01	0	1.09E+00	0	Single admission	3
2008	0	1.17E+01	0	8.05E+00	0	Single admission	3
2008	17	1.17E+01	0	7.91E-01	0	Single admission	3
2008	0	1.17E+01	0	6.98E-01	0	Single admission	3
2008	0	9.00E+00	0	1.43E+00	0	Single admission	3
2008	0	1.16E+01	0	5.52E+00	0	Single admission	3
2008	0	1.16E+01	0	7.14E+00	0	Repeated admissions	3
2008	1	1.25E+01	0	1.39E+00	0	Single admission	4
2008	0	2.09E-03	1	8.05E+01	1	Single admission	4
2008	0	1.24E+01	1	3.19E+00	0	Single admission	4
2008	1	1.24E+01	0	7.95E-01	0	Repeated admissions	4
2008	15	7.53E-01	0	2.42E+01	1	Single admission	4
2008	1	1.04E+01	0	4.24E+00	0	Single admission	4
2008	1	2.85E+00	0	2.87E+01	0	Single admission	4
2008	0	1.22E+01	0	1.42E+00	0	Single admission	4
2008	11	1.22E+01	0	9.21E-01	0	Single admission	4
2008	16	1.21E+01	0	8.59E+00	0	Single admission	4
2008	15	1.00E-01	0	9.90E-01	1	Single admission	4
2008	10	1.21E+01	0	3.01E+00	0	Single admission	4
2008	2	1.21E+01	0	7.51E-01	0	Single admission	4
2008	4	1.21E+01	1	1.60E+00	0	Single admission	4
2008	6	1.21E+01	1	3.78E+00	0	Single admission	4
2008	16	1.20E+01	0	3.99E+00	0	Single admission	4
2008	0	1.20E+01	1	1.29E+00	0	Single admission	4
2008	3	2.54E-03	1	1.83E+00	1	Single admission	4
2008	1	1.20E+01	1	3.88E+00	0	Single admission	4
2008	17	1.14E+01	0	1.06E+00	1	Repeated admissions	4
2008	16	1.18E+01	0	1.45E+00	0	Single admission	4
2008	3	1.18E+01	0	5.25E+00	0	Single admission	4

Admission year	Age (years)	Follow up time (years)	Sex (Females=1)	PDRLast	Outcome (Deceased=1)	Single/repeated admission	Diagnostic group
2008	17	1.18E+01	0	3.52E-01	0	Repeated admissions	4
2008	0	1.18E+01	1	4.17E-01	0	Repeated admissions	4
2008	7	1.18E+01	1	4.27E+00	0	Single admission	4
2008	1	1.18E+01	0	7.51E-01	0	Single admission	4
2008	0	6.15E-02	1	9.50E-01	1	Repeated admissions	4
2008	1	1.16E+01	0	1.53E+00	0	Single admission	4
2008	3	1.16E+01	0	7.69E+01	0	Single admission	4
2008	8	2.32E-02	0	5.60E+00	1	Single admission	4
2008	0	1.16E+01	0	1.33E+00	0	Single admission	4
2008	0	1.16E+01	0	4.24E+00	0	Single admission	4
2008	0	1.15E+01	1	7.51E-01	0	Repeated admissions	4
2008	2	1.25E+01	0	8.69E-01	0	Single admission	5
2008	1	1.24E+01	0	2.18E+01	0	Single admission	5
2008	15	1.80E+00	1	9.62E-01	1	Repeated admissions	5
2008	16	1.24E+01	1	1.41E-01	0	Single admission	5
2008	14	1.24E+01	1	1.32E-01	0	Repeated admissions	5
2008	8	1.23E+01	1	7.97E-01	0	Repeated admissions	5
2008	0	1.23E+01	1	2.25E+00	0	Single admission	5
2008	3	1.69E+00	0	1.50E-01	1	Repeated admissions	5
2008	12	1.21E+01	1	1.72E-01	0	Single admission	5
2008	3	1.21E+01	1	7.77E-01	0	Repeated admissions	5
2008	0	2.48E+00	0	2.12E-01	1	Repeated admissions	5
2008	0	1.21E+01	0	8.46E-01	0	Repeated admissions	5
2008	3	1.90E-01	1	2.35E+00	1	Single admission	5
2008	4	1.21E+01	0	1.76E-01	0	Single admission	5
2008	16	1.21E+01	1	8.39E-01	0	Repeated admissions	5
2008	5	1.20E+01	0	7.05E-01	0	Single admission	5
2008	6	1.20E+01	0	7.51E-01	0	Single admission	5
2008	9	1.19E+01	0	1.15E+00	0	Single admission	5
2008	9	1.19E+01	0	6.78E+00	0	Repeated admissions	5

Admission year	Age (years)	Follow up time (years)	Sex (Females=1)	PDRLast	Outcome (Deceased=1)	Single/repeated admission	Diagnostic group
2008	13	4.12E+00	1	1.15E+00	1	Repeated admissions	5
2008	11	1.09E+01	0	4.06E-01	1	Single admission	5
2008	2	1.18E+01	0	5.50E-01	0	Single admission	5
2008	8	1.90E+00	0	2.31E+01	1	Repeated admissions	5
2008	1	1.18E+01	0	2.22E-01	0	Single admission	5
2008	16	1.95E-01	0	3.07E+01	1	Single admission	5
2008	1	1.17E+01	1	1.07E-01	0	Single admission	5
2008	0	1.09E+00	1	1.48E+00	1	Single admission	5
2008	7	1.17E+01	0	1.25E-01	0	Repeated admissions	5
2008	3	1.16E+01	0	5.69E-01	0	Single admission	5
2008	6	5.49E-01	0	5.22E-01	1	Single admission	5
2008	0	1.16E+01	0	2.71E-01	0	Repeated admissions	5
2008	9	1.15E+01	1	1.83E-01	0	Single admission	5
2008	1	6.84E-03	1	9.98E+01	1	Single admission	5
2008	3	6.36E-01	0	7.51E-01	1	Single admission	5
2008	11	1.25E+01	1	7.72E-01	0	Single admission	6
2008	16	1.24E+01	1	1.59E+01	0	Single admission	6
2008	8	1.24E+01	0	3.83E-01	0	Single admission	6
2008	6	1.23E+01	1	2.87E+00	0	Repeated admissions	6
2008	2	1.23E+01	1	1.17E-01	0	Repeated admissions	6
2008	15	1.23E+01	0	6.17E+01	0	Single admission	6
2008	0	2.51E+00	1	5.71E+01	1	Single admission	6
2008	0	1.21E+01	0	1.36E-01	0	Repeated admissions	6
2008	1	1.21E+01	0	5.27E+00	0	Single admission	6
2008	0	1.20E+01	1	1.37E+00	0	Repeated admissions	6
2008	5	1.19E+01	1	4.27E+00	0	Single admission	6
2008	0	7.49E-03	1	4.24E+01	1	Single admission	6
2008	4	1.19E+01	0	1.72E+01	0	Single admission	6
2008	1	7.48E-01	1	2.01E+01	1	Single admission	6
2008	2	4.75E-03	0	5.66E+01	1	Single admission	6

Admission year	Age (years)	Follow up time (years)	Sex (Females=1)	PDRLast	Outcome (Deceased=1)	Single/repeated admission	Diagnostic group
2008	13	1.18E+01	0	1.38E+00	0	Repeated admissions	6
2008	13	1.17E+01	0	5.11E+00	0	Single admission	6
2008	9	1.17E+01	1	9.56E-01	0	Single admission	6
2008	1	1.15E-02	1	1.03E+01	1	Single admission	6
2008	0	1.16E+01	0	3.22E+00	0	Single admission	6
2008	0	1.16E+01	1	4.11E+01	0	Single admission	6
2008	0	1.16E+01	1	3.54E+00	0	Single admission	6
2008	16	1.15E+01	0	8.27E-01	0	Single admission	6
2008	2	4.47E-01	1	1.86E+00	0	Single admission	7
2008	12	1.24E+01	0	1.49E+00	0	Single admission	7
2008	17	1.24E+01	1	1.04E+00	0	Single admission	7
2008	3	1.24E+01	0	1.93E+00	0	Single admission	7
2008	4	1.23E+01	0	7.12E+00	0	Single admission	7
2008	14	1.23E+01	1	4.66E-01	0	Single admission	7
2008	14	1.23E+01	1	1.51E+00	0	Single admission	7
2008	16	1.23E+01	1	5.36E+00	0	Repeated admissions	7
2008	13	1.22E+01	1	5.97E-01	0	Single admission	7
2008	3	9.73E-01	0	2.72E+00	1	Repeated admissions	7
2008	11	1.22E+01	1	2.63E+00	0	Single admission	7
2008	0	2.74E-04	1	2.11E+01	1	Single admission	7
2008	0	2.74E-04	1	4.65E+01	1	Single admission	7
2008	8	1.21E+01	1	1.52E+00	0	Single admission	7
2008	5	1.20E+01	1	9.37E+00	0	Single admission	7
2008	2	1.19E+01	0	9.85E+00	0	Single admission	7
2008	10	1.19E+01	1	2.51E+00	0	Single admission	7
2008	15	1.18E+01	1	1.11E+00	0	Single admission	7
2008	9	1.18E+01	1	7.85E-01	0	Single admission	7
2008	16	1.18E+01	1	1.55E+01	0	Single admission	7
2008	0	1.18E+01	0	2.75E+00	0	Single admission	7
2008	13	1.18E+01	1	4.44E+00	0	Single admission	7

Admission year	Age (years)	Follow up time (years)	Sex (Females=1)	PDRLast	Outcome (Deceased=1)	Single/repeated admission	Diagnostic group
2008	3	1.18E+01	0	1.18E+00	0	Single admission	7
2008	0	1.18E+01	0	1.41E+01	0	Single admission	7
2008	11	1.18E+01	1	5.28E+00	0	Repeated admissions	7
2008	6	6.24E+00	1	2.33E+01	1	Repeated admissions	7
2008	17	1.17E+01	0	4.21E-01	0	Single admission	7
2008	11	1.17E+01	0	9.88E+00	0	Repeated admissions	7
2008	4	1.16E+01	0	1.46E+00	0	Single admission	7
2008	1	2.31E-01	0	4.41E+00	1	Single admission	7
2008	3	1.15E+01	1	2.85E+00	0	Single admission	7
2008	14	1.15E+01	0	9.85E-01	0	Repeated admissions	7
2008	0	1.15E+01	0	3.15E+00	0	Repeated admissions	7
2008	8	1.15E+01	1	9.72E+00	0	Single admission	7
2008	0	1.15E+01	0	1.41E+01	0	Repeated admissions	7
2008	13	1.15E+01	0	7.38E-01	0	Single admission	7
2008	2	1.15E+01	0	9.32E-01	0	Repeated admissions	7
2008	14	1.25E+01	1	1.03E+00	0	Single admission	8
2008	5	1.24E+01	1	3.46E+00	0	Single admission	8
2008	4	1.24E+01	0	9.90E-01	0	Single admission	8
2008	4	1.24E+01	0	1.07E-01	0	Repeated admissions	8
2008	18	1.24E+01	0	1.11E+00	0	Single admission	8
2008	18	1.24E+01	1	7.51E-01	0	Single admission	8
2008	14	1.23E+01	1	5.45E+01	0	Single admission	8
2008	2	1.23E+01	0	8.31E-01	0	Single admission	8
2008	10	1.23E+01	1	8.65E-01	0	Single admission	8
2008	1	1.23E+01	0	8.62E-01	0	Single admission	8
2008	6	1.23E+01	0	8.85E-01	0	Repeated admissions	8
2008	7	1.23E+01	0	1.81E-01	0	Repeated admissions	8
2008	6	1.22E+01	1	1.14E+00	0	Single admission	8
2008	13	1.22E+01	1	2.25E+00	0	Single admission	8
2008	14	1.22E+01	0	1.14E+00	0	Single admission	8

Admission year	Age (years)	Follow up time (years)	Sex (Females=1)	PDRLast	Outcome (Deceased=1)	Single/repeated admission	Diagnostic group
2008	0	1.22E+01	0	3.92E+00	0	Single admission	8
2008	17	1.22E+01	1	1.26E+00	0	Single admission	8
2008	18	1.22E+01	1	1.43E+01	0	Single admission	8
2008	18	1.21E+01	1	7.51E-01	0	Single admission	8
2008	5	1.21E+01	0	6.89E+00	0	Single admission	8
2008	6	1.21E+01	0	9.90E-01	0	Single admission	8
2008	5	1.21E+01	0	5.60E-01	0	Single admission	8
2008	7	1.21E+01	0	9.93E-01	0	Single admission	8
2008	8	1.21E+01	0	9.24E-01	0	Single admission	8
2008	3	1.21E+01	0	7.51E-01	0	Single admission	8
2008	6	1.21E+01	0	7.27E+00	0	Single admission	8
2008	18	7.57E+00	0	9.02E-01	0	Single admission	8
2008	17	1.13E+01	1	3.43E+00	0	Single admission	8
2008	14	1.20E+01	1	1.90E+00	0	Single admission	8
2008	18	1.09E+01	0	8.02E+00	1	Single admission	8
2008	5	1.20E+01	1	8.62E-01	0	Single admission	8
2008	6	1.20E+01	0	8.05E-01	0	Single admission	8
2008	13	1.20E+01	0	8.83E-01	0	Single admission	8
2008	10	1.20E+01	1	8.62E-01	0	Single admission	8
2008	15	1.20E+01	0	3.52E+00	0	Single admission	8
2008	8	1.20E+01	0	8.51E-01	0	Single admission	8
2008	15	1.20E+01	1	9.90E-01	0	Single admission	8
2008	17	1.20E+01	1	1.13E+00	0	Single admission	8
2008	12	1.20E+01	0	6.90E+00	0	Single admission	8
2008	14	1.20E+01	0	8.62E-01	0	Single admission	8
2008	8	1.19E+01	1	1.03E+00	0	Single admission	8
2008	1	1.19E+01	0	3.95E+01	0	Single admission	8
2008	7	1.19E+01	1	1.18E+00	0	Single admission	8
2008	2	1.19E+01	0	4.34E+00	0	Single admission	8
2008	2	1.19E+01	0	7.51E-01	0	Single admission	8

Admission year	Age (years)	Follow up time (years)	Sex (Females=1)	PDRLast	Outcome (Deceased=1)	Single/repeated admission	Diagnostic group
2008	4	1.19E+01	0	9.37E-01	0	Single admission	8
2008	3	1.19E+01	1	3.42E+00	0	Single admission	8
2008	1	1.19E+01	0	5.22E+00	0	Single admission	8
2008	12	1.19E+01	0	8.05E-01	0	Single admission	8
2008	5	1.19E+01	0	4.19E+00	0	Single admission	8
2008	8	1.19E+01	0	1.03E+00	0	Single admission	8
2008	14	1.19E+01	0	1.39E+00	0	Single admission	8
2008	13	1.19E+01	0	4.08E+00	0	Single admission	8
2008	1	1.19E+01	1	2.26E+01	0	Single admission	8
2008	3	1.18E+01	1	1.04E+00	0	Single admission	8
2008	10	1.18E+01	1	9.37E-01	0	Single admission	8
2008	9	1.18E+01	0	1.13E+00	0	Single admission	8
2008	17	1.18E+01	1	7.51E-01	0	Single admission	8
2008	3	1.18E+01	0	1.09E+00	0	Single admission	8
2008	11	1.18E+01	0	7.83E-01	0	Single admission	8
2008	13	1.18E+01	0	3.38E-01	0	Repeated admissions	8
2008	17	1.18E+01	0	1.45E+00	0	Single admission	8
2008	7	1.18E+01	0	9.76E-01	0	Single admission	8
2008	14	1.17E+01	1	1.78E+00	0	Single admission	8
2008	3	1.17E+01	1	1.27E+00	0	Single admission	8
2008	16	1.17E+01	1	7.51E-01	0	Single admission	8
2008	11	1.17E+01	0	7.72E-01	0	Single admission	8
2008	17	1.17E+01	1	2.19E+00	0	Single admission	8
2008	14	1.17E+01	1	3.59E+00	0	Single admission	8
2008	18	1.17E+01	1	8.62E-01	0	Single admission	8
2008	11	1.17E+01	1	2.41E+00	0	Single admission	8
2008	15	1.16E+01	1	9.90E-01	0	Single admission	8
2008	8	1.16E+01	1	7.51E-01	0	Single admission	8
2008	6	1.16E+01	0	9.77E-01	0	Single admission	8
2008	10	1.16E+01	0	8.92E-01	0	Single admission	8

Admission year	Age (years)	Follow up time (years)	Sex (Females=1)	PDRLast	Outcome (Deceased=1)	Single/repeated admission	Diagnostic group
2008	16	1.16E+01	0	1.56E+00	0	Single admission	8
2008	1	1.16E+01	0	6.72E+00	0	Single admission	8
2008	17	1.16E+01	1	1.17E+00	0	Single admission	8
2008	17	1.16E+01	0	1.21E+00	0	Single admission	8
2008	17	1.16E+01	1	8.65E-01	0	Single admission	8
2008	2	1.16E+01	0	6.01E+00	0	Single admission	8
2008	14	1.16E+01	1	1.41E+01	0	Single admission	8
2008	15	1.16E+01	0	3.88E+00	0	Single admission	8
2008	18	1.16E+01	1	7.51E-01	0	Single admission	8
2008	17	1.15E+01	1	7.51E-01	0	Single admission	8
2008	14	2.73E-03	0	8.97E+01	1	Single admission	8
2008	0	1.15E+01	0	7.51E-01	0	Single admission	8
2008	15	1.15E+01	0	7.51E-01	0	Single admission	8
2008	14	1.15E+01	0	1.98E+01	0	Single admission	8
2008	1	1.25E+01	0	7.51E-01	0	Single admission	9
2008	1	1.25E+01	0	4.11E-01	0	Single admission	9
2008	3	1.25E+01	0	3.39E-01	0	Single admission	9
2008	5	1.24E+01	1	5.92E-01	0	Single admission	9
2008	7	1.24E+01	1	1.07E-01	0	Single admission	9
2008	2	1.24E+01	0	3.76E-01	0	Single admission	9
2008	13	1.24E+01	1	7.04E-01	0	Single admission	9
2008	3	1.23E+01	1	1.03E+00	0	Single admission	9
2008	6	1.23E+01	0	7.51E-01	0	Repeated admissions	9
2008	0	1.23E+01	0	1.46E+00	0	Single admission	9
2008	4	1.40E+00	0	7.78E-01	1	Single admission	9
2008	0	1.23E+01	0	5.69E-01	0	Single admission	9
2008	4	1.23E+01	1	2.79E+00	0	Single admission	9
2008	15	1.23E+01	0	2.54E+00	0	Single admission	9
2008	12	1.22E+01	1	1.16E+00	0	Single admission	9
2008	0	1.22E+01	0	8.07E-01	0	Single admission	9

Admission year	Age (years)	Follow up time (years)	Sex (Females=1)	PDRLast	Outcome (Deceased=1)	Single/repeated admission	Diagnostic group
2008	5	1.22E+01	0	2.94E-01	0	Single admission	9
2008	0	1.22E+01	0	3.80E+00	0	Single admission	9
2008	4	1.21E+01	1	2.48E+00	0	Single admission	9
2008	3	1.21E+01	1	1.58E+00	0	Single admission	9
2008	1	1.21E+01	1	1.98E+00	0	Repeated admissions	9
2008	0	1.21E+01	0	1.42E-01	0	Single admission	9
2008	11	1.21E+01	0	1.06E+01	0	Single admission	9
2008	0	1.21E+01	0	3.86E+00	0	Single admission	9
2008	3	1.21E+01	0	2.84E-01	0	Single admission	9
2008	2	1.21E+01	0	1.03E+00	0	Repeated admissions	9
2008	0	1.21E+01	1	1.43E+00	0	Single admission	9
2008	0	1.21E+01	0	1.40E+00	0	Single admission	9
2008	7	1.21E+01	1	1.07E-01	0	Repeated admissions	9
2008	17	1.21E+01	1	8.10E-01	0	Single admission	9
2008	15	1.20E+01	0	8.42E-01	0	Single admission	9
2008	0	1.19E+01	0	3.22E-01	0	Single admission	9
2008	0	1.19E+01	0	4.06E-01	0	Single admission	9
2008	17	1.19E+01	1	8.65E-01	0	Single admission	9
2008	2	1.19E+01	1	1.63E-01	0	Single admission	9
2008	0	1.19E+01	0	5.69E-01	0	Single admission	9
2008	0	1.19E+01	0	3.57E+01	0	Single admission	9
2008	6	1.18E+01	0	7.51E-01	0	Single admission	9
2008	5	1.18E+01	0	9.23E-01	0	Repeated admissions	9
2008	13	1.18E+01	1	1.12E-01	0	Single admission	9
2008	15	1.18E+01	0	1.38E-01	0	Repeated admissions	9
2008	15	1.18E+01	0	1.23E-01	0	Single admission	9
2008	0	1.18E+01	1	7.51E-01	0	Single admission	9
2008	0	1.18E+01	1	2.71E-01	0	Single admission	9
2008	10	3.37E+00	0	2.71E-01	0	Single admission	9
2008	13	1.17E+01	1	7.51E-01	0	Repeated admissions	9

Admission year	Age (years)	Follow up time (years)	Sex (Females=1)	PDRLast	Outcome (Deceased=1)	Single/repeated admission	Diagnostic group
2008	0	1.17E+01	1	2.98E-01	0	Single admission	9
2008	0	1.17E+01	0	1.07E-01	0	Single admission	9
2008	14	6.06E+00	0	3.43E+00	1	Single admission	9
2008	14	1.02E+01	0	1.25E+01	1	Single admission	9
2008	10	9.68E+00	1	3.28E+00	1	Repeated admissions	9
2008	0	1.17E+01	1	1.29E-01	0	Single admission	9
2008	1	1.16E+01	1	1.07E-01	0	Repeated admissions	9
2008	0	1.16E+01	1	7.51E-01	0	Repeated admissions	9
2008	13	1.16E+01	0	2.15E+00	0	Single admission	9
2008	15	1.15E+01	0	1.65E-01	0	Single admission	9
2008	10	2.28E+00	1	3.86E+00	1	Single admission	9
2008	15	1.15E+01	1	8.62E-01	0	Single admission	9
2008	0	1.15E+01	0	7.51E-01	0	Repeated admissions	9
2008	0	1.15E+01	1	2.80E+00	0	Repeated admissions	9
2008	0	1.24E+01	0	1.64E+00	0	Repeated admissions	10
2008	1	1.24E+01	0	9.90E-01	0	Single admission	10
2008	3	1.24E+01	1	1.61E+00	0	Repeated admissions	10
2008	6	1.24E+01	1	3.43E+00	0	Single admission	10
2008	2	1.24E+01	1	1.18E+00	0	Single admission	10
2008	4	6.90E+00	1	4.11E+01	1	Single admission	10
2008	8	1.24E+01	1	4.84E+00	0	Single admission	10
2008	1	5.33E-01	0	8.41E-01	1	Repeated admissions	10
2008	11	1.23E+01	1	3.88E+00	0	Repeated admissions	10
2008	12	7.72E+00	1	1.58E+00	1	Repeated admissions	10
2008	2	1.22E+01	0	3.39E+00	0	Single admission	10
2008	7	1.22E+01	1	8.86E-01	0	Repeated admissions	10
2008	8	1.22E+01	1	1.44E+00	0	Single admission	10
2008	2	1.22E+01	0	3.88E+00	0	Repeated admissions	10
2008	0	1.22E+01	0	2.35E-01	0	Single admission	10
2008	13	1.21E+01	1	9.24E-01	0	Single admission	10

Admission year	Age (years)	Follow up time (years)	Sex (Females=1)	PDRLast	Outcome (Deceased=1)	Single/repeated admission	Diagnostic group
2008	1	1.21E+01	1	1.14E+00	0	Single admission	10
2008	2	1.21E+01	1	7.51E-01	0	Single admission	10
2008	16	1.21E+01	0	9.53E-01	0	Single admission	10
2008	2	1.20E+01	1	7.51E-01	0	Single admission	10
2008	11	1.20E+01	0	1.53E+00	0	Single admission	10
2008	6	1.20E+01	0	1.48E+00	0	Single admission	10
2008	14	1.20E+01	0	1.85E+00	0	Repeated admissions	10
2008	5	1.19E+01	1	9.63E-01	0	Single admission	10
2008	1	1.19E+01	0	6.02E+00	0	Single admission	10
2008	14	1.19E+01	0	1.10E+00	0	Repeated admissions	10
2008	2	1.18E+01	1	2.51E+00	0	Single admission	10
2008	15	1.18E+01	1	7.51E-01	0	Repeated admissions	10
2008	5	3.35E+00	1	3.88E+00	0	Repeated admissions	10
2008	13	1.18E+01	0	4.42E+00	0	Single admission	10
2008	2	1.18E+01	0	1.31E+00	0	Single admission	10
2008	0	7.70E-04	0	4.52E+01	1	Single admission	10
2008	0	2.71E-02	1	2.27E+01	1	Repeated admissions	10
2008	2	1.18E+01	1	9.90E-01	0	Repeated admissions	10
2008	0	1.17E+01	0	1.02E+00	0	Single admission	10
2008	0	1.17E+01	1	7.51E-01	0	Single admission	10
2008	3	1.17E+01	0	8.68E-01	0	Single admission	10
2008	0	1.17E+01	1	1.75E+00	0	Single admission	10
2008	2	1.17E+01	1	7.51E-01	0	Single admission	10
2008	8	1.17E+01	0	3.88E+00	0	Single admission	10
2008	3	1.17E+01	1	1.14E+00	0	Single admission	10
2008	11	1.16E+01	0	8.62E-01	0	Single admission	10
2008	18	1.16E+01	1	3.53E+00	0	Single admission	10
2008	5	2.18E+00	1	9.41E+01	1	Repeated admissions	10
2008	10	1.16E+01	1	1.19E+00	0	Single admission	10
2008	4	1.16E+01	0	1.18E-01	0	Repeated admissions	10

Admission year	Age (years)	Follow up time (years)	Sex (Females=1)	PDRLast	Outcome (Deceased=1)	Single/repeated admission	Diagnostic group
2008	5	4.19E+00	1	8.67E+01	1	Repeated admissions	10
2008	2	1.16E+01	0	7.41E-02	0	Single admission	10
2008	6	1.16E+01	0	6.20E+00	0	Single admission	10
2008	8	1.15E+01	0	5.50E+00	0	Repeated admissions	10
2008	0	1.15E+01	1	7.51E-01	0	Single admission	10
2008	4	1.15E+01	0	1.23E+00	0	Single admission	10
2008	10	1.15E+01	0	7.51E-01	0	Single admission	10
2008	1	1.15E+01	0	2.82E+00	0	Single admission	10
2008	3	1.15E+01	0	7.51E-01	0	Repeated admissions	10
2008	14	1.15E+01	0	8.05E-01	0	Repeated admissions	10
2008	7	1.15E+01	0	4.46E+01	0	Single admission	10
2008	0	1.25E+01	1	1.60E+00	0	Single admission	11
2008	1	1.24E+01	1	3.13E-01	0	Single admission	11
2008	12	1.24E+01	0	1.22E+01	0	Single admission	11
2008	1	1.09E+00	0	1.14E+00	1	Repeated admissions	11
2008	1	1.24E+01	1	2.37E-01	0	Single admission	11
2008	4	1.24E+01	1	3.13E-01	0	Single admission	11
2008	0	1.24E+01	0	2.06E+00	0	Single admission	11
2008	1	1.78E+00	0	2.13E-01	1	Repeated admissions	11
2008	3	2.58E-01	0	3.02E+01	1	Repeated admissions	11
2008	0	1.23E+01	1	1.56E+00	0	Single admission	11
2008	1	1.23E+01	0	2.14E-01	0	Repeated admissions	11
2008	0	1.23E+01	0	1.70E-01	0	Single admission	11
2008	1	1.23E+01	1	1.42E-01	0	Repeated admissions	11
2008	1	1.23E+01	1	3.44E-01	0	Single admission	11
2008	0	1.22E+01	0	1.52E+00	0	Single admission	11
2008	0	1.22E+01	0	1.80E-01	0	Single admission	11
2008	0	1.22E+01	0	3.11E+01	0	Repeated admissions	11
2008	7	1.22E+01	0	1.38E-01	0	Single admission	11
2008	1	1.22E+01	1	1.54E-01	0	Single admission	11

Admission year	Age (years)	Follow up time (years)	Sex (Females=1)	PDRLast	Outcome (Deceased=1)	Single/repeated admission	Diagnostic group
2008	0	1.21E+01	0	1.67E+00	0	Single admission	11
2008	0	2.03E-01	0	9.51E-01	1	Single admission	11
2008	0	4.83E+00	0	1.94E+01	0	Single admission	11
2008	1	1.21E+01	0	1.42E-01	0	Single admission	11
2008	1	1.21E+01	1	3.38E-01	0	Single admission	11
2008	1	1.21E+01	1	4.06E-01	0	Repeated admissions	11
2008	0	1.21E+01	0	6.31E+00	0	Single admission	11
2008	2	1.21E+01	0	1.75E-01	0	Single admission	11
2008	0	1.21E+01	1	9.60E+00	0	Single admission	11
2008	0	1.21E+01	0	3.05E+00	0	Single admission	11
2008	0	1.21E+01	1	2.22E+01	0	Single admission	11
2008	0	1.20E+01	1	6.07E+00	0	Single admission	11
2008	0	1.20E+01	0	4.78E+00	0	Single admission	11
2008	1	1.20E+01	1	1.21E+01	0	Single admission	11
2008	0	1.20E+01	1	2.56E+00	0	Single admission	11
2008	0	1.20E+01	0	5.44E+00	0	Single admission	11
2008	0	1.20E+01	1	8.82E+00	0	Single admission	11
2008	0	1.20E+01	0	8.71E-01	0	Single admission	11
2008	0	1.20E+01	1	2.12E+01	0	Single admission	11
2008	0	1.19E+01	1	2.98E-01	0	Single admission	11
2008	0	1.19E+01	0	9.96E+01	0	Single admission	11
2008	0	1.19E+01	1	1.74E+00	0	Single admission	11
2008	3	1.19E+01	0	1.14E+00	0	Repeated admissions	11
2008	6	1.19E+01	0	3.03E-01	0	Repeated admissions	11
2008	0	1.19E+01	0	3.26E-01	0	Single admission	11
2008	0	1.19E+01	1	1.30E+00	0	Repeated admissions	11
2008	0	1.19E+01	1	9.54E+00	0	Single admission	11
2008	1	1.19E+01	1	6.02E+00	0	Repeated admissions	11
2008	0	1.18E+01	0	3.88E+00	0	Single admission	11
2008	1	1.18E+01	1	1.12E-01	0	Single admission	11

Admission year	Age (years)	Follow up time (years)	Sex (Females=1)	PDRLast	Outcome (Deceased=1)	Single/repeated admission	Diagnostic group
2008	12	1.18E+01	0	9.04E-01	0	Repeated admissions	11
2008	0	1.18E+01	1	2.47E-01	0	Single admission	11
2008	0	1.09E+00	1	1.99E+00	0	Single admission	11
2008	0	1.17E+01	0	7.51E-01	0	Repeated admissions	11
2008	0	3.29E-01	0	1.26E+01	1	Single admission	11
2008	0	1.17E+01	0	7.51E-01	0	Repeated admissions	11
2008	0	1.17E+01	0	2.49E+01	0	Single admission	11
2008	0	1.17E+01	0	1.65E+00	0	Single admission	11
2008	0	1.17E+01	0	1.60E-01	0	Single admission	11
2008	0	2.48E+00	1	1.60E+00	0	Repeated admissions	11
2008	1	1.16E+01	1	2.55E+00	0	Single admission	11
2008	1	6.07E-01	0	4.41E+01	1	Repeated admissions	11
2008	0	1.16E+01	1	2.01E-01	0	Single admission	11
2008	0	1.16E+01	0	1.75E-01	0	Single admission	11
2008	0	1.16E+01	0	2.48E-01	0	Single admission	11
2008	0	1.16E+01	0	1.10E+01	0	Single admission	11
2008	0	1.16E+01	0	1.07E-01	0	Single admission	11
2008	0	1.16E+01	0	1.27E+00	0	Single admission	11
2008	0	1.16E+01	0	1.44E+00	0	Single admission	11
2008	0	1.16E+01	1	9.84E-01	0	Single admission	11
2008	1	1.16E+01	0	1.87E-01	0	Single admission	11
2008	1	1.15E+01	1	7.51E-01	0	Repeated admissions	11
2008	2	1.25E+01	0	4.67E+00	0	Single admission	12
2008	2	1.25E+01	1	3.46E-01	0	Single admission	12
2008	1	1.25E+01	1	2.74E-01	0	Single admission	12
2008	0	1.25E+01	0	1.87E-01	0	Single admission	12
2008	0	1.25E+01	0	5.25E-01	0	Single admission	12
2008	0	1.25E+01	0	3.72E-01	0	Single admission	12
2008	0	1.24E+01	0	8.27E-01	0	Single admission	12
2008	0	1.24E+01	1	1.31E+00	0	Single admission	12

Admission year	Age (years)	Follow up time (years)	Sex (Females=1)	PDRLast	Outcome (Deceased=1)	Single/repeated admission	Diagnostic group
2008	0	1.24E+01	0	4.29E-01	0	Single admission	12
2008	1	1.24E+01	1	1.75E-01	0	Repeated admissions	12
2008	0	1.24E+01	1	4.20E+00	0	Single admission	12
2008	2	1.24E+01	1	1.56E-01	0	Single admission	12
2008	5	1.24E+01	0	2.71E-01	0	Repeated admissions	12
2008	0	1.24E+01	1	3.26E+00	0	Single admission	12
2008	0	1.24E+01	1	8.47E+00	0	Single admission	12
2008	4	1.24E+01	0	3.91E+00	0	Repeated admissions	12
2008	0	1.24E+01	0	7.51E-01	0	Single admission	12
2008	0	1.24E+01	0	2.46E+00	0	Single admission	12
2008	7	3.20E+00	1	8.73E+00	1	Repeated admissions	12
2008	3	1.24E+01	1	7.51E-01	0	Single admission	12
2008	0	1.24E+01	1	2.33E+00	0	Single admission	12
2008	0	1.24E+01	0	1.56E-01	0	Single admission	12
2008	0	1.24E+01	0	8.33E-01	0	Single admission	12
2008	1	1.24E+01	0	9.82E-01	0	Single admission	12
2008	2	1.24E+01	0	7.51E-01	0	Single admission	12
2008	6	1.24E+01	0	1.21E+00	0	Single admission	12
2008	0	1.24E+01	1	1.50E+00	0	Single admission	12
2008	10	1.24E+01	1	2.72E+00	0	Single admission	12
2008	0	1.24E+01	0	5.91E-01	0	Single admission	12
2008	0	1.24E+01	0	2.64E-01	0	Single admission	12
2008	0	1.24E+01	0	3.67E+00	0	Single admission	12
2008	1	1.23E+01	0	1.56E-01	0	Single admission	12
2008	0	1.23E+01	1	4.70E+00	0	Single admission	12
2008	0	1.23E+01	1	2.39E+00	0	Single admission	12
2008	4	1.23E+01	1	5.16E+00	0	Single admission	12
2008	0	1.23E+01	0	7.51E-01	0	Single admission	12
2008	0	1.23E+01	1	5.76E+00	0	Single admission	12
2008	2	1.05E-01	1	1.21E+01	1	Single admission	12

Admission year	Age (years)	Follow up time (years)	Sex (Females=1)	PDRLast	Outcome (Deceased=1)	Single/repeated admission	Diagnostic group
2008	1	1.23E+01	1	4.65E+00	0	Single admission	12
2008	5	1.23E+01	0	4.30E+00	0	Repeated admissions	12
2008	9	1.23E+01	1	7.78E+00	0	Repeated admissions	12
2008	0	1.23E+01	0	1.73E-01	0	Repeated admissions	12
2008	0	1.23E+01	1	5.91E-01	0	Single admission	12
2008	11	1.23E+01	1	1.33E+01	0	Repeated admissions	12
2008	3	1.23E+01	0	3.63E+00	0	Single admission	12
2008	0	1.23E+01	0	4.50E+00	0	Single admission	12
2008	4	3.64E+00	0	7.51E-01	1	Repeated admissions	12
2008	2	1.22E+01	1	5.75E+00	0	Repeated admissions	12
2008	13	1.22E+01	1	2.98E-01	0	Single admission	12
2008	9	1.22E+01	0	8.14E-01	0	Single admission	12
2008	8	8.45E+00	0	2.04E+01	1	Repeated admissions	12
2008	3	1.22E+01	0	8.14E-01	0	Single admission	12
2008	3	1.22E+01	1	5.15E+00	0	Single admission	12
2008	14	1.22E+01	0	7.50E+00	0	Single admission	12
2008	1	1.22E+01	1	5.94E+00	0	Single admission	12
2008	7	1.22E+01	0	1.42E-01	0	Repeated admissions	12
2008	0	1.22E+01	0	5.39E+00	0	Single admission	12
2008	11	1.22E+01	0	4.29E-01	0	Single admission	12
2008	4	1.22E+01	1	8.60E+00	0	Single admission	12
2008	4	1.21E+01	0	3.58E-01	0	Single admission	12
2008	0	1.21E+01	0	6.30E+00	0	Single admission	12
2008	1	1.21E+01	1	5.10E-01	0	Repeated admissions	12
2008	6	1.21E+01	0	9.39E+00	0	Single admission	12
2008	1	1.21E+01	1	7.51E-01	0	Single admission	12
2008	8	1.21E+01	0	2.70E+01	0	Single admission	12
2008	3	1.21E+01	0	5.36E-01	0	Single admission	12
2008	0	1.21E+01	0	1.67E+00	0	Single admission	12
2008	18	1.21E+01	0	5.46E-01	0	Single admission	12

Admission year	Age (years)	Follow up time (years)	Sex (Females=1)	PDRLast	Outcome (Deceased=1)	Single/repeated admission	Diagnostic group
2008	9	1.21E+01	1	7.51E-01	0	Single admission	12
2008	8	1.21E+01	1	1.15E+00	0	Single admission	12
2008	1	1.21E+01	0	7.51E-01	0	Single admission	12
2008	1	1.21E+01	0	3.88E+00	0	Single admission	12
2008	1	1.20E+01	0	2.36E-01	0	Single admission	12
2008	3	5.96E-01	1	5.12E+00	1	Repeated admissions	12
2008	0	1.20E+01	1	1.03E+00	0	Single admission	12
2008	3	1.20E+01	0	2.09E+01	0	Single admission	12
2008	1	1.20E+01	0	2.60E+00	0	Repeated admissions	12
2008	0	1.20E+01	0	1.70E-01	0	Repeated admissions	12
2008	0	1.20E+01	0	2.76E+00	0	Single admission	12
2008	12	2.34E-01	1	4.18E+00	1	Single admission	12
2008	3	1.18E+01	0	1.26E+00	0	Single admission	12
2008	2	1.18E+01	0	5.71E+00	0	Single admission	12
2008	7	1.18E+01	1	1.11E+00	0	Repeated admissions	12
2008	1	4.74E-01	0	1.34E+01	1	Repeated admissions	12
2008	4	1.18E+01	1	1.34E+00	0	Single admission	12
2008	11	1.18E+01	1	2.13E+00	0	Single admission	12
2008	1	1.18E+01	1	9.53E+00	0	Single admission	12
2008	7	8.87E+00	0	9.90E-01	1	Repeated admissions	12
2008	4	1.18E+01	0	1.46E-01	0	Repeated admissions	12
2008	1	1.18E+01	1	7.51E-01	0	Single admission	12
2008	1	1.18E+01	1	9.46E-01	0	Single admission	12
2008	17	1.23E-02	0	2.90E+01	1	Single admission	12
2008	2	1.18E+01	1	7.51E-01	0	Single admission	12
2008	1	1.18E+01	1	7.51E-01	0	Single admission	12
2008	6	1.18E+01	0	1.06E+01	0	Repeated admissions	12
2008	9	1.17E+01	1	1.02E+00	0	Repeated admissions	12
2008	7	1.17E+01	0	2.94E-01	0	Repeated admissions	12
2008	0	1.17E+01	0	1.09E+00	0	Single admission	12

Admission year	Age (years)	Follow up time (years)	Sex (Females=1)	PDRLast	Outcome (Deceased=1)	Single/repeated admission	Diagnostic group
2008	13	2.74E-04	0	8.59E-01	1	Single admission	12
2008	0	1.16E+01	1	2.15E+00	0	Single admission	12
2008	2	1.16E+01	0	2.62E+00	0	Repeated admissions	12
2008	11	2.97E+00	1	1.33E+01	1	Repeated admissions	12
2008	0	1.16E+01	0	2.11E+00	0	Single admission	12
2008	0	1.16E+01	1	5.40E+00	0	Single admission	12
2008	4	1.16E+01	0	6.27E+00	0	Repeated admissions	12
2008	2	1.16E+01	1	3.88E+00	0	Single admission	12
2008	17	1.16E+01	0	7.51E-01	0	Repeated admissions	12
2008	15	1.12E+01	1	1.63E-01	1	Repeated admissions	12
2008	2	1.16E+01	0	4.31E+00	0	Single admission	12
2008	1	1.16E+01	0	2.57E-01	0	Single admission	12
2008	17	1.16E+01	0	1.45E+00	0	Single admission	12
2008	18	1.15E+01	1	1.08E+00	0	Repeated admissions	12
2008	2	1.15E+01	0	1.18E+00	0	Single admission	12
2008	1	1.15E+01	1	1.35E+00	0	Single admission	12
2008	3	1.15E+01	1	7.51E-01	0	Repeated admissions	12
2008	0	1.15E+01	0	7.45E+00	0	Repeated admissions	12
2008	10	1.15E+01	0	7.51E-01	0	Single admission	12
2008	2	1.15E+01	0	7.51E-01	0	Single admission	12
2008	0	1.15E+01	1	8.27E-01	0	Repeated admissions	12
2008	9	1.15E+01	0	7.21E-02	0	Repeated admissions	12
2008	1	1.15E+01	1	1.56E-01	0	Single admission	12
2008	8	1.15E+01	1	7.85E+00	0	Single admission	12
2008	1	1.15E+01	0	3.88E+00	0	Single admission	12
2008	0	1.15E+01	0	1.56E-01	0	Single admission	12
2009	3	1.13E+01	0	1.60E+00	0	Single admission	1
2009	11	1.10E+01	0	9.24E-01	0	Single admission	1
2009	2	1.08E+01	1	1.92E+00	0	Repeated admissions	1
2009	6	6.88E+00	0	4.85E+00	1	Repeated admissions	1

Admission year	Age (years)	Follow up time (years)	Sex (Females=1)	PDRLast	Outcome (Deceased=1)	Single/repeated admission	Diagnostic group
2009	6	1.05E+01	0	1.22E+00	0	Single admission	1
2009	0	1.15E+01	1	1.10E+01	0	Single admission	2
2009	0	1.15E+01	1	2.80E+00	0	Single admission	2
2009	0	1.14E+01	0	1.55E+00	0	Single admission	2
2009	0	1.13E+01	0	1.10E+00	0	Single admission	2
2009	1	1.11E+01	1	1.00E+00	0	Single admission	2
2009	0	1.90E-01	1	4.29E+01	1	Repeated admissions	2
2009	0	1.09E+01	0	1.92E+00	0	Single admission	2
2009	0	1.08E+01	0	1.76E+01	0	Single admission	2
2009	0	1.08E+01	0	1.59E+00	0	Single admission	2
2009	0	1.07E+01	1	5.69E-01	0	Repeated admissions	2
2009	0	1.06E+01	0	6.77E+00	0	Single admission	2
2009	0	1.06E+01	1	1.46E+00	0	Repeated admissions	2
2009	0	1.05E+01	1	1.70E+01	0	Single admission	2
2009	2	1.14E+01	0	2.09E+00	0	Single admission	3
2009	1	1.14E+01	0	9.37E+00	0	Single admission	3
2009	2	1.14E+01	1	1.52E+00	0	Single admission	3
2009	0	1.14E+01	0	1.72E+00	0	Single admission	3
2009	0	5.74E+00	1	5.60E+00	0	Single admission	3
2009	0	1.13E+01	0	7.51E-01	0	Repeated admissions	3
2009	2	1.13E+01	0	5.28E+00	0	Single admission	3
2009	0	1.12E+01	1	2.54E+00	0	Single admission	3
2009	0	1.12E+01	1	2.47E-01	0	Single admission	3
2009	1	1.12E+01	0	1.30E+00	0	Single admission	3
2009	13	1.12E+01	0	5.43E-01	0	Single admission	3
2009	12	1.12E+01	1	4.42E-01	0	Single admission	3
2009	0	1.11E+01	0	3.48E+00	0	Single admission	3
2009	1	1.11E+01	0	6.36E+00	0	Single admission	3
2009	1	1.11E+01	1	1.11E+00	0	Repeated admissions	3
2009	1	1.11E+01	0	6.23E-01	0	Single admission	3

Admission year	Age (years)	Follow up time (years)	Sex (Females=1)	PDRLast	Outcome (Deceased=1)	Single/repeated admission	Diagnostic group
2009	0	1.11E+01	0	1.77E-01	0	Single admission	3
2009	1	1.10E+01	0	1.18E+00	0	Single admission	3
2009	0	1.10E+01	1	7.44E-01	0	Repeated admissions	3
2009	2	1.10E+01	1	7.97E-01	0	Repeated admissions	3
2009	0	1.10E+01	0	1.97E+00	0	Single admission	3
2009	1	1.10E+01	1	1.08E+00	0	Single admission	3
2009	4	1.09E+01	0	1.07E-01	0	Single admission	3
2009	0	1.09E+01	0	6.23E-01	0	Single admission	3
2009	11	1.09E+01	0	9.89E-01	0	Single admission	3
2009	0	6.24E+00	0	1.43E+00	0	Repeated admissions	3
2009	0	1.03E+01	0	7.51E-01	0	Single admission	3
2009	0	1.08E+01	0	1.07E-01	0	Single admission	3
2009	0	1.08E+01	0	9.24E-01	0	Single admission	3
2009	1	4.77E+00	1	3.88E+00	0	Repeated admissions	3
2009	3	1.08E+01	1	2.00E+01	0	Repeated admissions	3
2009	0	1.08E+01	1	7.51E-01	0	Repeated admissions	3
2009	3	1.07E+01	0	2.71E-01	0	Single admission	3
2009	0	1.07E+01	1	1.50E+00	0	Repeated admissions	3
2009	17	1.07E+01	1	2.04E+00	0	Single admission	3
2009	1	8.96E-01	1	2.10E+01	1	Repeated admissions	3
2009	1	1.05E+01	1	2.84E+00	0	Repeated admissions	3
2009	4	1.05E+01	1	1.07E+00	0	Single admission	3
2009	0	1.15E+01	0	1.67E+00	0	Single admission	4
2009	13	1.14E+01	0	7.51E-01	0	Single admission	4
2009	3	1.13E+01	0	4.67E+01	0	Single admission	4
2009	2	1.12E+01	0	3.63E+00	0	Single admission	4
2009	1	1.12E+01	1	1.81E-01	0	Single admission	4
2009	0	1.11E+01	0	7.51E-01	0	Repeated admissions	4
2009	0	1.11E+01	0	4.05E+00	0	Repeated admissions	4
2009	15	1.11E+01	1	2.75E+00	0	Repeated admissions	4

Admission year	Age (years)	Follow up time (years)	Sex (Females=1)	PDRLast	Outcome (Deceased=1)	Single/repeated admission	Diagnostic group
2009	2	3.74E-01	0	1.52E-01	1	Repeated admissions	4
2009	13	1.10E+01	1	1.14E+00	0	Single admission	4
2009	14	1.09E+01	1	8.33E-01	0	Single admission	4
2009	1	1.09E+01	0	5.58E+00	0	Single admission	4
2009	5	1.09E+01	1	1.93E+00	0	Repeated admissions	4
2009	0	2.74E-04	0	5.87E+01	1	Single admission	4
2009	4	5.33E+00	1	3.88E+00	1	Single admission	4
2009	4	2.43E+00	1	9.73E+01	1	Repeated admissions	4
2009	0	1.07E+01	0	6.77E+00	0	Single admission	4
2009	18	1.07E+01	1	1.84E+01	0	Single admission	4
2009	0	7.92E+00	1	4.13E+00	0	Single admission	4
2009	13	1.07E+01	0	1.51E+00	0	Single admission	4
2009	0	1.06E+01	0	3.88E+00	0	Single admission	4
2009	3	2.74E-04	0	7.27E+00	1	Single admission	4
2009	13	1.06E+01	0	1.27E+00	0	Repeated admissions	4
2009	1	1.06E+01	0	1.03E+00	0	Single admission	4
2009	0	1.06E+01	0	3.88E+00	0	Single admission	4
2009	16	1.06E+01	0	4.82E+00	0	Single admission	4
2009	6	1.06E+01	0	1.40E+00	0	Single admission	4
2009	9	2.99E-03	1	4.52E+01	1	Single admission	4
2009	0	3.07E-02	1	3.88E+00	1	Single admission	4
2009	2	1.15E+01	0	7.23E+00	0	Single admission	5
2009	12	1.14E+01	0	1.54E-01	0	Single admission	5
2009	11	1.14E+01	0	2.10E-01	0	Repeated admissions	5
2009	1	1.14E+01	0	3.88E+00	0	Single admission	5
2009	1	7.08E-01	1	7.51E-01	1	Repeated admissions	5
2009	14	2.31E+00	0	3.54E-01	1	Single admission	5
2009	7	1.12E+01	0	1.07E-01	0	Single admission	5
2009	2	1.12E+01	1	5.82E-01	0	Single admission	5
2009	13	1.11E+01	0	6.16E-01	0	Single admission	5

Admission year	Age (years)	Follow up time (years)	Sex (Females=1)	PDRLast	Outcome (Deceased=1)	Single/repeated admission	Diagnostic group
2009	11	4.33E-01	0	4.78E+01	1	Single admission	5
2009	7	1.11E+01	0	7.51E-01	0	Single admission	5
2009	2	1.11E+01	0	6.07E-01	0	Single admission	5
2009	2	1.11E+01	1	3.56E+00	0	Single admission	5
2009	5	5.00E-01	0	4.22E-01	1	Single admission	5
2009	0	1.10E+01	0	1.17E+00	0	Repeated admissions	5
2009	2	2.57E+00	0	6.15E+00	1	Repeated admissions	5
2009	8	1.10E+01	0	4.11E-01	0	Single admission	5
2009	3	1.10E+01	0	5.69E-01	0	Single admission	5
2009	6	8.87E+00	0	7.23E+00	0	Single admission	5
2009	0	1.08E+01	1	7.15E+00	0	Single admission	5
2009	2	1.08E+01	0	7.51E-01	0	Single admission	5
2009	3	1.08E+01	1	1.06E+00	0	Single admission	5
2009	15	1.07E+01	0	1.52E-01	0	Single admission	5
2009	6	1.07E+01	1	2.98E-01	0	Single admission	5
2009	1	1.06E+01	0	9.37E-01	0	Single admission	5
2009	7	1.22E-01	0	2.28E+00	1	Repeated admissions	5
2009	1	1.06E+01	0	9.37E-01	0	Single admission	5
2009	7	1.06E+01	0	1.07E-01	0	Single admission	5
2009	17	2.31E-02	0	5.67E+00	1	Single admission	5
2009	0	1.06E+01	0	2.66E+00	0	Single admission	5
2009	0	4.09E-01	0	4.57E+01	1	Single admission	5
2009	7	4.47E-04	0	2.80E+01	1	Single admission	5
2009	0	5.23E-02	1	7.51E-01	1	Repeated admissions	5
2009	0	1.05E+01	0	5.00E-01	0	Single admission	5
2009	1	1.05E+01	0	7.51E-01	0	Single admission	5
2009	2	5.49E-02	1	8.52E+00	1	Repeated admissions	6
2009	15	1.14E+01	1	3.91E+00	0	Single admission	6
2009	13	1.14E+01	0	7.83E-01	0	Single admission	6
2009	9	5.98E-03	1	2.31E+01	1	Single admission	6

Admission year	Age (years)	Follow up time (years)	Sex (Females=1)	PDRLast	Outcome (Deceased=1)	Single/repeated admission	Diagnostic group
2009	6	1.14E+01	0	1.09E+00	0	Single admission	6
2009	6	1.14E+01	0	1.39E+00	0	Single admission	6
2009	16	1.14E+01	0	8.99E-01	0	Single admission	6
2009	0	8.54E-03	1	2.79E+01	1	Single admission	6
2009	15	1.13E+01	1	3.48E+00	0	Single admission	6
2009	11	1.12E+01	0	1.14E+00	0	Single admission	6
2009	0	1.12E+01	0	7.51E-01	0	Single admission	6
2009	14	1.12E+01	1	9.59E-01	0	Single admission	6
2009	0	1.61E-02	0	9.25E+01	1	Single admission	6
2009	10	1.11E+01	0	7.51E-01	0	Repeated admissions	6
2009	9	1.09E+01	0	9.06E+01	0	Single admission	6
2009	7	1.09E+01	1	9.90E-01	0	Single admission	6
2009	14	1.09E+01	0	3.11E-01	0	Single admission	6
2009	0	1.09E+01	0	1.58E+00	0	Repeated admissions	6
2009	1	1.09E+01	0	5.90E+00	0	Single admission	6
2009	1	1.08E+01	1	1.67E+01	0	Repeated admissions	6
2009	15	1.07E+01	1	8.51E-01	0	Single admission	6
2009	11	1.07E+01	1	5.63E+01	0	Single admission	6
2009	6	1.07E+01	0	1.27E+00	0	Single admission	6
2009	7	1.07E+01	0	1.78E+01	0	Repeated admissions	6
2009	9	1.60E-03	0	9.84E+01	1	Single admission	6
2009	1	1.07E+01	0	8.24E-01	0	Single admission	6
2009	6	3.13E-03	1	2.18E+01	1	Single admission	6
2009	0	1.06E+01	1	1.36E+01	0	Single admission	6
2009	11	1.06E+01	1	7.51E-01	0	Single admission	6
2009	0	1.06E+01	0	7.51E-01	0	Single admission	6
2009	5	1.06E+01	1	2.82E+01	0	Repeated admissions	6
2009	2	1.05E+01	0	1.50E+00	0	Single admission	6
2009	6	1.62E+00	1	2.61E+00	1	Repeated admissions	7
2009	5	1.13E+01	1	1.26E+00	0	Single admission	7

Admission year	Age (years)	Follow up time (years)	Sex (Females=1)	PDRLast	Outcome (Deceased=1)	Single/repeated admission	Diagnostic group
2009	3	1.13E+01	0	1.26E+00	0	Single admission	7
2009	13	1.13E+01	0	2.98E+00	0	Single admission	7
2009	12	1.12E+01	0	1.30E+00	0	Single admission	7
2009	7	1.12E+01	0	1.15E+00	0	Single admission	7
2009	14	1.12E+01	0	9.40E+00	0	Single admission	7
2009	15	1.11E+01	1	1.64E+00	0	Single admission	7
2009	18	1.10E+01	0	5.96E-01	0	Single admission	7
2009	17	1.10E+01	0	6.26E-01	0	Single admission	7
2009	3	1.09E+01	1	7.72E+00	0	Single admission	7
2009	0	1.09E+01	1	8.30E-01	0	Single admission	7
2009	10	1.08E+01	0	5.88E+00	0	Single admission	7
2009	8	1.08E+01	0	1.37E+00	0	Single admission	7
2009	14	1.07E+01	1	2.78E+00	0	Single admission	7
2009	0	2.74E-04	1	7.22E+01	1	Single admission	7
2009	0	1.07E+01	0	2.61E+00	0	Single admission	7
2009	12	1.06E+01	0	4.69E+00	0	Single admission	7
2009	14	1.06E+01	0	1.71E+00	0	Single admission	7
2009	1	1.06E+01	0	1.45E+01	0	Repeated admissions	7
2009	0	1.06E+01	1	2.14E+00	0	Repeated admissions	7
2009	8	1.06E+01	0	1.33E+00	0	Single admission	7
2009	11	1.05E+01	0	9.24E-01	0	Single admission	7
2009	12	1.05E+01	1	9.58E-01	0	Single admission	7
2009	11	1.05E+01	1	7.51E-01	0	Single admission	7
2009	15	1.15E+01	1	8.27E-01	0	Single admission	8
2009	11	1.14E+01	1	3.88E+00	0	Single admission	8
2009	1	1.14E+01	1	7.51E-01	0	Single admission	8
2009	12	1.14E+01	0	9.24E-01	0	Single admission	8
2009	3	1.14E+01	0	9.90E-01	0	Single admission	8
2009	5	1.14E+01	1	7.51E-01	0	Single admission	8
2009	0	1.13E+01	0	1.57E+00	0	Repeated admissions	8

Admission year	Age (years)	Follow up time (years)	Sex (Females=1)	PDRLast	Outcome (Deceased=1)	Single/repeated admission	Diagnostic group
2009	8	1.13E+01	1	7.51E-01	0	Repeated admissions	8
2009	8	1.12E+01	1	5.28E+00	0	Single admission	8
2009	14	1.12E+01	1	3.46E+00	0	Repeated admissions	8
2009	15	1.12E+01	1	5.22E+00	0	Single admission	8
2009	7	1.12E+01	1	1.60E+00	0	Single admission	8
2009	8	1.12E+01	1	7.51E-01	0	Single admission	8
2009	14	1.12E+01	0	8.27E-01	0	Single admission	8
2009	15	1.12E+01	1	9.37E-01	0	Single admission	8
2009	8	1.11E+01	0	2.04E+00	0	Single admission	8
2009	1	1.11E+01	1	3.88E+00	0	Single admission	8
2009	10	1.11E+01	0	7.51E-01	0	Single admission	8
2009	4	1.11E+01	0	7.51E-01	0	Single admission	8
2009	7	1.34E+00	1	7.51E-01	0	Single admission	8
2009	14	1.11E+01	0	7.51E-01	0	Single admission	8
2009	10	1.11E+01	0	4.52E-01	0	Repeated admissions	8
2009	14	1.11E+01	1	1.53E+00	0	Single admission	8
2009	10	1.10E+01	0	8.39E-01	0	Single admission	8
2009	14	1.10E+01	1	3.88E+00	0	Single admission	8
2009	17	1.10E+01	1	9.90E-01	0	Single admission	8
2009	18	1.10E+01	1	2.21E+00	0	Single admission	8
2009	4	1.10E+01	0	1.22E+00	0	Single admission	8
2009	5	1.10E+01	0	1.81E+00	0	Repeated admissions	8
2009	18	1.10E+01	1	3.39E+00	0	Single admission	8
2009	1	1.10E+01	0	2.44E+01	0	Single admission	8
2009	12	1.31E+00	0	8.74E-01	0	Single admission	8
2009	1	1.09E+01	0	7.51E-01	0	Single admission	8
2009	6	1.09E+01	1	7.51E-01	0	Single admission	8
2009	2	1.09E+01	0	9.41E+01	0	Single admission	8
2009	13	1.09E+01	0	7.51E-01	0	Single admission	8
2009	18	1.09E+01	0	1.58E+00	0	Single admission	8

Admission year	Age (years)	Follow up time (years)	Sex (Females=1)	PDRLast	Outcome (Deceased=1)	Single/repeated admission	Diagnostic group
2009	2	1.15E-02	1	9.47E+01	1	Single admission	8
2009	3	1.09E+01	0	1.19E+00	0	Repeated admissions	8
2009	4	1.08E+01	1	3.88E+00	0	Single admission	8
2009	1	6.64E+00	0	2.00E+00	0	Single admission	8
2009	5	1.08E+01	1	1.29E+00	0	Single admission	8
2009	16	1.08E+01	1	7.51E-01	0	Single admission	8
2009	6	1.08E+01	0	9.24E-01	0	Single admission	8
2009	6	1.08E+01	1	3.07E+01	0	Single admission	8
2009	8	1.08E+01	0	3.88E+00	0	Repeated admissions	8
2009	3	1.08E+01	0	1.06E+00	0	Single admission	8
2009	3	1.08E+01	0	3.88E+00	0	Single admission	8
2009	6	1.08E+01	1	7.51E-01	0	Single admission	8
2009	9	1.08E+01	1	9.24E-01	0	Single admission	8
2009	16	1.08E+01	0	3.88E+00	0	Single admission	8
2009	10	1.08E+01	0	7.59E-01	0	Single admission	8
2009	3	1.08E+01	1	3.88E+00	0	Single admission	8
2009	1	1.08E+01	0	1.00E+00	0	Single admission	8
2009	11	1.07E+01	0	3.88E+00	0	Single admission	8
2009	10	1.07E+01	1	1.06E+00	0	Single admission	8
2009	12	1.07E+01	0	3.88E+00	0	Repeated admissions	8
2009	12	1.06E+01	0	1.09E+00	0	Single admission	8
2009	7	1.06E+01	1	5.45E+00	0	Single admission	8
2009	17	8.35E+00	1	9.37E-01	0	Single admission	8
2009	15	1.05E+01	1	7.51E-01	0	Single admission	8
2009	16	1.05E+01	1	7.51E-01	0	Single admission	8
2009	3	1.05E+01	0	1.06E+01	0	Repeated admissions	8
2009	9	1.05E+01	0	3.91E+00	0	Single admission	8
2009	3	1.14E+01	0	7.51E-01	0	Repeated admissions	9
2009	1	1.14E+01	0	1.07E-01	0	Repeated admissions	9
2009	15	1.14E+01	1	9.82E-01	0	Single admission	9

Admission year	Age (years)	Follow up time (years)	Sex (Females=1)	PDRLast	Outcome (Deceased=1)	Single/repeated admission	Diagnostic group
2009	2	1.14E+01	1	1.30E+00	0	Single admission	9
2009	1	1.14E+01	0	1.42E-01	0	Single admission	9
2009	7	1.14E+01	0	1.07E-01	0	Single admission	9
2009	6	1.14E+01	1	2.71E-01	0	Single admission	9
2009	12	1.14E+01	1	2.71E-01	0	Single admission	9
2009	14	1.14E+01	0	2.71E-01	0	Single admission	9
2009	1	1.13E+01	1	7.51E-01	0	Single admission	9
2009	2	1.13E+01	1	2.98E-01	0	Single admission	9
2009	0	1.13E+01	0	1.07E-01	0	Single admission	9
2009	8	1.13E+01	0	1.14E+00	0	Repeated admissions	9
2009	2	1.12E+01	1	1.23E-01	0	Single admission	9
2009	14	1.12E+01	0	8.62E-01	0	Repeated admissions	9
2009	16	1.12E+01	1	1.07E-01	0	Repeated admissions	9
2009	0	1.12E+01	1	1.90E-01	0	Single admission	9
2009	6	1.11E+01	0	7.51E-01	0	Single admission	9
2009	1	1.11E+01	0	3.91E-01	0	Single admission	9
2009	8	1.11E+01	0	9.24E-01	0	Single admission	9
2009	3	1.11E+01	1	1.20E+00	0	Single admission	9
2009	0	1.11E+01	1	3.35E-01	0	Single admission	9
2009	8	1.10E+01	1	7.51E-01	0	Single admission	9
2009	2	1.10E+01	0	5.22E+00	0	Single admission	9
2009	0	1.10E+01	0	5.22E+00	0	Single admission	9
2009	0	1.09E+01	0	5.69E-01	0	Repeated admissions	9
2009	3	1.09E+01	1	5.69E-01	0	Repeated admissions	9
2009	0	1.09E+01	0	2.71E-01	0	Single admission	9
2009	0	1.08E+01	0	2.12E-01	0	Single admission	9
2009	1	1.08E+01	1	9.13E-01	0	Single admission	9
2009	17	1.08E+01	0	1.95E+00	0	Repeated admissions	9
2009	3	1.65E-01	0	1.11E+00	0	Repeated admissions	9
2009	0	1.08E+01	1	1.72E-01	0	Single admission	9

Admission year	Age (years)	Follow up time (years)	Sex (Females=1)	PDRLast	Outcome (Deceased=1)	Single/repeated admission	Diagnostic group
2009	1	1.08E+01	1	1.87E-01	0	Single admission	9
2009	1	1.08E+01	0	7.51E-01	0	Single admission	9
2009	0	1.07E+01	1	2.15E+00	0	Single admission	9
2009	1	1.07E+01	0	1.43E+00	0	Single admission	9
2009	0	1.07E+01	1	1.43E+00	0	Single admission	9
2009	6	1.07E+01	1	1.07E-01	0	Single admission	9
2009	0	1.07E+01	0	5.69E-01	0	Single admission	9
2009	3	1.07E+01	1	4.37E+00	0	Single admission	9
2009	0	1.07E+01	0	2.65E-01	0	Single admission	9
2009	15	1.06E+01	1	1.24E+00	0	Repeated admissions	9
2009	6	1.06E+01	1	1.07E-01	0	Single admission	9
2009	10	1.06E+01	1	1.07E-01	0	Single admission	9
2009	0	1.06E+01	0	5.22E+00	0	Single admission	9
2009	11	1.06E+01	0	2.31E+00	0	Single admission	9
2009	0	1.06E+01	0	2.69E-01	0	Single admission	9
2009	0	1.05E+01	0	3.78E-01	0	Single admission	9
2009	16	1.05E+01	1	7.51E-01	0	Single admission	9
2009	5	1.15E+01	1	9.63E-01	0	Single admission	10
2009	1	1.14E+01	0	3.88E+00	0	Repeated admissions	10
2009	3	7.84E+00	1	1.43E+00	1	Repeated admissions	10
2009	0	1.14E+01	1	8.62E-01	0	Single admission	10
2009	4	1.14E+01	0	7.51E-01	0	Single admission	10
2009	4	1.14E+01	0	1.56E-01	0	Repeated admissions	10
2009	2	1.14E+01	1	1.97E+00	0	Single admission	10
2009	7	1.13E+01	1	4.31E+00	0	Single admission	10
2009	7	1.13E+01	1	3.88E+00	0	Single admission	10
2009	12	1.13E+01	0	2.03E+00	0	Single admission	10
2009	2	1.13E+01	0	1.54E+00	0	Single admission	10
2009	1	1.12E+01	1	1.38E+00	0	Single admission	10
2009	2	1.12E+01	1	1.70E+00	0	Single admission	10

Admission year	Age (years)	Follow up time (years)	Sex (Females=1)	PDRLast	Outcome (Deceased=1)	Single/repeated admission	Diagnostic group
2009	0	1.12E+01	0	1.25E+00	0	Single admission	10
2009	2	1.12E+01	0	2.71E+00	0	Single admission	10
2009	1	1.12E+01	0	1.49E+00	0	Single admission	10
2009	0	1.12E+01	0	1.95E-01	0	Repeated admissions	10
2009	17	1.12E+01	0	7.51E-01	0	Repeated admissions	10
2009	2	1.11E+01	0	3.88E+00	0	Single admission	10
2009	2	1.11E+01	0	9.24E-02	0	Single admission	10
2009	16	5.13E+00	1	7.51E-01	1	Repeated admissions	10
2009	7	1.11E+01	0	7.94E-01	0	Repeated admissions	10
2009	0	1.11E+01	0	1.15E+00	0	Single admission	10
2009	6	1.11E+01	0	7.94E-01	0	Single admission	10
2009	7	1.10E+01	1	1.93E-01	0	Repeated admissions	10
2009	6	1.10E+01	1	2.30E+00	0	Single admission	10
2009	6	1.10E+01	1	1.25E+00	0	Single admission	10
2009	11	1.10E+01	0	9.90E-01	0	Single admission	10
2009	17	4.23E+00	0	1.64E-01	1	Repeated admissions	10
2009	1	1.10E+01	1	5.65E+00	0	Repeated admissions	10
2009	1	1.10E+01	0	1.69E+00	0	Single admission	10
2009	2	1.10E+01	0	1.16E+00	0	Single admission	10
2009	0	1.10E+01	0	6.14E+00	0	Single admission	10
2009	6	1.10E+01	1	1.83E+00	0	Single admission	10
2009	9	1.27E-03	0	3.25E+01	1	Single admission	10
2009	13	1.10E+01	0	3.91E+00	0	Single admission	10
2009	6	1.09E+01	1	8.87E-01	0	Repeated admissions	10
2009	15	1.09E+01	1	7.51E-01	0	Repeated admissions	10
2009	1	1.09E+01	0	5.18E+00	0	Single admission	10
2009	2	1.08E+01	0	1.10E+01	0	Single admission	10
2009	0	7.51E-01	1	5.72E+00	1	Single admission	10
2009	0	1.08E+01	0	8.62E-01	0	Repeated admissions	10
2009	3	1.07E+01	0	3.95E+00	0	Single admission	10

Admission year	Age (years)	Follow up time (years)	Sex (Females=1)	PDRLast	Outcome (Deceased=1)	Single/repeated admission	Diagnostic group
2009	0	1.07E+01	1	7.51E-01	0	Repeated admissions	10
2009	2	1.06E+01	0	6.36E+00	0	Single admission	10
2009	2	1.06E+01	0	1.33E+00	0	Repeated admissions	10
2009	15	1.06E+01	0	7.51E-01	0	Repeated admissions	10
2009	1	1.06E+01	1	3.36E+00	0	Single admission	10
2009	10	1.05E+01	1	7.51E-01	0	Repeated admissions	10
2009	8	1.05E+01	1	5.08E+01	0	Single admission	10
2009	0	1.15E+01	1	5.69E-01	0	Repeated admissions	11
2009	0	1.15E+01	1	7.51E-01	0	Repeated admissions	11
2009	1	1.15E+01	0	2.71E-01	0	Repeated admissions	11
2009	0	1.14E+01	1	8.10E-01	0	Single admission	11
2009	0	1.14E+01	0	1.33E+01	0	Single admission	11
2009	2	1.14E+01	1	4.01E+00	0	Single admission	11
2009	0	1.14E+01	0	2.64E+01	0	Single admission	11
2009	0	1.14E+01	0	8.82E-01	0	Single admission	11
2009	0	1.14E+01	0	2.46E-01	0	Single admission	11
2009	0	1.14E+01	0	7.51E-01	0	Repeated admissions	11
2009	2	1.14E+01	0	5.43E-01	0	Single admission	11
2009	2	1.14E+01	1	4.59E-01	0	Repeated admissions	11
2009	0	1.14E+01	1	2.86E+00	0	Single admission	11
2009	1	6.87E+00	1	4.72E-01	1	Repeated admissions	11
2009	0	1.14E+01	0	2.89E-01	0	Repeated admissions	11
2009	0	1.13E+01	1	2.25E+01	0	Repeated admissions	11
2009	3	1.13E+01	1	1.07E-01	0	Repeated admissions	11
2009	1	1.13E+01	0	1.07E-01	0	Single admission	11
2009	0	1.13E+01	1	1.30E+00	0	Single admission	11
2009	11	1.12E+01	1	2.76E-01	0	Repeated admissions	11
2009	0	1.12E+01	1	1.90E+00	0	Single admission	11
2009	0	1.12E+01	0	2.16E+01	0	Single admission	11
2009	0	1.12E+01	0	1.00E+01	0	Single admission	11

Admission year	Age (years)	Follow up time (years)	Sex (Females=1)	PDRLast	Outcome (Deceased=1)	Single/repeated admission	Diagnostic group
2009	7	1.12E+01	1	1.07E-01	0	Single admission	11
2009	0	1.69E-02	0	1.09E+01	1	Single admission	11
2009	0	1.12E+01	0	2.39E+01	0	Repeated admissions	11
2009	0	1.12E+01	0	1.70E+00	0	Single admission	11
2009	0	1.11E+01	1	4.02E+00	0	Single admission	11
2009	7	1.11E+01	0	3.51E+00	0	Single admission	11
2009	0	1.11E+01	1	2.17E+01	0	Single admission	11
2009	0	1.11E+01	1	1.13E+01	0	Single admission	11
2009	0	1.11E+01	0	3.51E+00	0	Repeated admissions	11
2009	0	1.11E+01	0	1.02E+01	0	Single admission	11
2009	0	1.11E+01	0	3.13E-01	0	Single admission	11
2009	1	1.11E+01	0	5.09E-01	0	Single admission	11
2009	0	1.11E+01	0	1.63E+00	0	Single admission	11
2009	3	1.11E+01	1	5.36E-01	0	Single admission	11
2009	0	1.10E+01	0	7.51E-01	0	Single admission	11
2009	0	1.10E+01	1	5.38E+00	0	Repeated admissions	11
2009	0	1.10E+01	1	2.59E+00	0	Single admission	11
2009	0	1.10E+01	0	3.81E+00	0	Repeated admissions	11
2009	0	1.09E+01	0	4.54E-01	0	Single admission	11
2009	0	1.09E+01	0	2.71E-01	0	Single admission	11
2009	0	1.09E+01	0	3.69E+01	0	Single admission	11
2009	2	1.09E+01	1	1.83E+00	0	Repeated admissions	11
2009	0	1.09E+01	0	1.56E+00	0	Single admission	11
2009	0	1.09E+01	1	2.71E-01	0	Single admission	11
2009	0	1.09E+01	0	1.54E-01	0	Single admission	11
2009	0	1.09E+01	1	1.77E+00	0	Single admission	11
2009	0	1.09E+01	0	1.30E-01	0	Single admission	11
2009	0	1.09E+01	0	3.88E+00	0	Repeated admissions	11
2009	2	1.09E+01	1	3.54E-01	0	Repeated admissions	11
2009	7	1.08E+01	0	4.44E+00	0	Repeated admissions	11

Admission year	Age (years)	Follow up time (years)	Sex (Females=1)	PDRLast	Outcome (Deceased=1)	Single/repeated admission	Diagnostic group
2009	0	1.08E+01	0	1.57E+00	0	Single admission	11
2009	3	1.08E+01	1	5.69E-01	0	Repeated admissions	11
2009	0	1.08E+01	0	1.22E+01	0	Single admission	11
2009	0	1.08E+01	0	3.36E+00	0	Single admission	11
2009	3	3.44E+00	1	9.28E+01	1	Repeated admissions	11
2009	0	1.08E+01	0	4.72E-01	0	Single admission	11
2009	0	1.08E+01	0	3.03E-01	0	Single admission	11
2009	0	1.08E+01	0	7.51E-01	0	Repeated admissions	11
2009	0	1.08E+01	0	7.77E+00	0	Repeated admissions	11
2009	0	3.68E-01	1	1.78E+01	1	Repeated admissions	11
2009	0	1.07E+01	1	1.50E+00	0	Single admission	11
2009	0	1.07E+01	0	1.85E+00	0	Single admission	11
2009	2	1.07E+01	0	7.61E-01	0	Repeated admissions	11
2009	0	1.07E+01	0	3.88E+00	0	Single admission	11
2009	0	1.07E+01	0	3.88E+00	0	Single admission	11
2009	0	1.07E+01	0	4.26E+00	0	Single admission	11
2009	0	1.07E+01	0	2.49E+00	0	Single admission	11
2009	0	1.07E+01	1	1.07E-01	0	Single admission	11
2009	0	1.07E+01	0	1.02E+00	0	Repeated admissions	11
2009	4	6.68E+00	0	1.37E+00	1	Repeated admissions	11
2009	0	1.06E+01	0	8.49E+00	0	Repeated admissions	11
2009	0	1.06E+01	1	5.14E+00	0	Single admission	11
2009	0	1.06E+01	1	2.36E+00	0	Single admission	11
2009	0	1.06E+01	0	1.67E+00	0	Single admission	11
2009	0	2.00E+00	0	3.54E+00	1	Repeated admissions	11
2009	0	1.06E+01	1	2.71E-01	0	Repeated admissions	11
2009	0	8.58E-02	0	6.06E+01	1	Single admission	11
2009	0	1.05E+01	0	3.00E+00	0	Single admission	11
2009	7	1.05E+01	1	1.73E+00	0	Repeated admissions	11
2009	0	1.05E+01	0	7.25E+00	0	Single admission	11

Admission year	Age (years)	Follow up time (years)	Sex (Females=1)	PDRLast	Outcome (Deceased=1)	Single/repeated admission	Diagnostic group
2009	0	1.05E+01	0	2.78E+00	0	Single admission	11
2009	1	1.15E+01	0	1.76E+00	0	Single admission	12
2009	12	1.40E-01	0	9.92E+01	1	Repeated admissions	12
2009	14	1.15E+01	0	4.19E+00	0	Repeated admissions	12
2009	0	1.15E+01	0	1.34E+01	0	Single admission	12
2009	2	8.33E-01	1	2.71E-01	1	Repeated admissions	12
2009	15	1.14E+01	0	1.14E+00	0	Repeated admissions	12
2009	4	1.14E+01	1	7.67E-01	0	Single admission	12
2009	1	1.14E+01	0	7.97E+00	0	Single admission	12
2009	4	8.40E-03	1	1.60E+00	1	Single admission	12
2009	0	1.14E+01	1	1.56E-01	0	Single admission	12
2009	9	4.09E+00	0	3.12E+01	1	Repeated admissions	12
2009	1	1.14E+01	0	9.62E-01	0	Single admission	12
2009	0	1.14E+01	0	6.33E-01	0	Single admission	12
2009	0	1.14E+01	1	8.27E-01	0	Single admission	12
2009	5	6.31E+00	1	4.66E+00	1	Repeated admissions	12
2009	0	1.14E+01	1	4.16E-01	0	Single admission	12
2009	1	1.14E+01	1	4.36E+00	0	Single admission	12
2009	0	1.14E+01	0	2.62E+00	0	Single admission	12
2009	0	1.13E+01	1	1.56E-01	0	Single admission	12
2009	0	1.13E+01	1	7.00E+00	0	Single admission	12
2009	0	1.13E+01	0	2.27E-01	0	Single admission	12
2009	0	1.13E+01	0	1.99E+00	0	Single admission	12
2009	0	1.13E+01	0	8.27E-01	0	Single admission	12
2009	1	1.13E+01	0	2.54E+00	0	Single admission	12
2009	0	1.13E+01	1	7.51E-01	0	Single admission	12
2009	0	1.13E+01	1	5.91E-01	0	Repeated admissions	12
2009	0	1.13E+01	0	3.88E+00	0	Single admission	12
2009	0	1.13E+01	0	3.88E+00	0	Repeated admissions	12
2009	0	1.13E+01	0	3.40E+00	0	Repeated admissions	12

Admission year	Age (years)	Follow up time (years)	Sex (Females=1)	PDRLast	Outcome (Deceased=1)	Single/repeated admission	Diagnostic group
2009	0	1.13E+01	1	2.16E+00	0	Single admission	12
2009	0	1.13E+01	0	6.48E-01	0	Single admission	12
2009	0	1.13E+01	1	2.22E-01	0	Single admission	12
2009	0	1.13E+01	1	8.05E-01	0	Single admission	12
2009	5	1.13E+01	0	3.18E-02	0	Repeated admissions	12
2009	0	1.13E+01	1	1.10E+00	0	Single admission	12
2009	7	1.13E+01	1	2.20E+00	0	Repeated admissions	12
2009	0	1.13E+01	1	3.00E+00	0	Single admission	12
2009	2	1.13E+01	0	3.39E+00	0	Single admission	12
2009	4	1.13E+01	1	1.57E+00	0	Repeated admissions	12
2009	0	1.13E+01	0	1.10E+00	0	Single admission	12
2009	0	1.13E+01	0	5.91E-01	0	Single admission	12
2009	0	1.13E+01	0	6.73E-01	0	Single admission	12
2009	0	1.13E+01	0	2.30E-01	0	Single admission	12
2009	0	1.13E+01	0	1.97E-01	0	Single admission	12
2009	1	1.12E+01	0	1.19E+00	0	Single admission	12
2009	17	1.12E+01	0	9.42E-02	0	Repeated admissions	12
2009	0	1.12E+01	0	1.56E-01	0	Single admission	12
2009	2	1.12E+01	1	1.17E+00	0	Single admission	12
2009	1	1.12E+01	0	8.27E-01	0	Repeated admissions	12
2009	1	1.12E+01	0	1.79E-01	0	Single admission	12
2009	2	1.12E+01	0	3.09E-01	0	Single admission	12
2009	0	1.12E+01	0	1.73E-01	0	Single admission	12
2009	0	1.12E+01	1	1.57E+01	0	Single admission	12
2009	7	1.12E+01	0	8.62E-01	0	Single admission	12
2009	2	1.12E+01	0	3.58E-01	0	Single admission	12
2009	3	1.12E+01	1	1.06E+00	0	Single admission	12
2009	0	1.12E+01	0	1.11E+00	0	Single admission	12
2009	4	1.12E+01	0	1.56E-01	0	Single admission	12
2009	0	1.12E+01	0	2.64E+00	0	Single admission	12

Admission year	Age (years)	Follow up time (years)	Sex (Females=1)	PDRLast	Outcome (Deceased=1)	Single/repeated admission	Diagnostic group
2009	1	1.12E+01	1	3.88E+00	0	Repeated admissions	12
2009	1	1.12E+01	1	1.40E+01	0	Single admission	12
2009	1	1.12E+01	0	1.56E-01	0	Single admission	12
2009	2	3.98E+00	0	1.02E+00	1	Repeated admissions	12
2009	16	9.97E+00	0	1.35E+00	1	Single admission	12
2009	0	1.11E+01	0	1.56E-01	0	Single admission	12
2009	2	1.11E+01	0	5.61E-02	0	Single admission	12
2009	1	1.11E+01	0	3.56E+01	0	Single admission	12
2009	6	1.11E+01	0	2.80E+00	0	Single admission	12
2009	3	1.11E+01	0	9.29E+00	0	Single admission	12
2009	1	1.11E+01	1	1.02E+00	0	Repeated admissions	12
2009	12	1.11E+01	0	2.24E-01	0	Repeated admissions	12
2009	2	1.11E+01	1	1.95E+01	0	Single admission	12
2009	4	1.11E+01	0	1.48E+00	0	Single admission	12
2009	1	1.11E+01	0	8.27E-01	0	Repeated admissions	12
2009	2	1.11E+01	0	1.56E-01	0	Single admission	12
2009	0	1.10E+01	1	7.51E-01	0	Single admission	12
2009	0	1.10E+01	1	2.01E+01	0	Single admission	12
2009	0	1.10E+01	0	8.27E-01	0	Single admission	12
2009	0	1.10E+01	0	1.50E+00	0	Single admission	12
2009	1	1.10E+01	0	7.13E+00	0	Repeated admissions	12
2009	11	1.10E+01	1	1.15E+00	0	Single admission	12
2009	7	3.25E+00	0	8.25E+00	1	Single admission	12
2009	0	1.41E+00	0	7.51E-01	0	Single admission	12
2009	0	1.30E-01	0	1.72E+00	1	Single admission	12
2009	7	1.10E+01	1	2.34E-01	0	Repeated admissions	12
2009	1	1.09E+01	0	2.76E+00	0	Single admission	12
2009	4	1.09E+01	1	3.47E+01	0	Repeated admissions	12
2009	2	1.09E+01	1	7.28E+00	0	Single admission	12
2009	15	1.08E+01	0	3.31E-01	0	Single admission	12

Admission year	Age (years)	Follow up time (years)	Sex (Females=1)	PDRLast	Outcome (Deceased=1)	Single/repeated admission	Diagnostic group
2009	2	1.08E+01	1	6.90E-01	0	Repeated admissions	12
2009	11	2.80E+00	0	2.57E+00	1	Single admission	12
2009	15	1.08E+01	1	8.62E-01	0	Single admission	12
2009	2	1.08E+01	0	2.36E-01	0	Single admission	12
2009	0	1.08E+01	0	1.02E+00	0	Single admission	12
2009	2	1.08E+01	0	3.88E+00	0	Single admission	12
2009	0	1.08E+01	0	6.75E+00	0	Single admission	12
2009	0	1.08E+01	0	1.07E-01	0	Repeated admissions	12
2009	1	1.07E+01	0	8.11E+00	0	Single admission	12
2009	5	4.23E+00	0	4.56E+00	1	Repeated admissions	12
2009	4	1.07E+01	0	1.06E+00	0	Repeated admissions	12
2009	1	1.06E+01	1	2.80E+00	0	Repeated admissions	12
2009	10	5.08E+00	0	3.24E+01	1	Repeated admissions	12
2009	3	4.76E-02	0	9.71E+00	1	Single admission	12
2009	0	1.06E+01	0	5.69E-01	0	Repeated admissions	12
2009	3	1.06E+01	0	4.23E-01	0	Repeated admissions	12
2009	2	1.63E+00	0	1.39E+00	1	Repeated admissions	12
2009	5	1.33E-03	0	4.11E+01	1	Single admission	12
2009	3	1.06E+01	1	3.88E+00	0	Single admission	12
2009	7	1.06E+01	1	2.20E-01	0	Single admission	12
2009	0	1.06E+01	1	4.52E+01	0	Single admission	12
2009	17	7.92E-03	0	1.64E+01	1	Single admission	12
2009	1	1.05E+01	0	8.27E-01	0	Single admission	12
2009	6	7.71E-01	1	1.41E+01	1	Single admission	12
2009	5	1.25E+00	0	8.27E-01	0	Single admission	12
2009	0	1.05E+01	0	1.45E+01	0	Single admission	12
2009	0	1.05E+01	0	1.43E+00	0	Single admission	12
2009	1	8.53E+00	1	2.16E+00	0	Single admission	12
2009	0	1.05E+01	1	1.56E-01	0	Single admission	12
2009	1	1.05E+01	1	1.50E+00	0	Single admission	12

Admission year	Age (years)	Follow up time (years)	Sex (Females=1)	PDRLast	Outcome (Deceased=1)	Single/repeated admission	Diagnostic group
2009	1	1.05E+01	0	7.51E-01	0	Single admission	12
2009	0	1.05E+01	0	3.36E-01	0	Single admission	12
2010	6	9.93E+00	0	1.12E+00	0	Repeated admissions	1
2010	0	7.44E+00	1	5.06E+00	0	Single admission	1
2010	2	9.76E+00	1	8.17E+00	0	Repeated admissions	1
2010	1	2.02E+00	0	1.23E+00	1	Repeated admissions	1
2010	2	9.71E+00	0	1.61E+00	0	Repeated admissions	1
2010	0	1.05E+01	0	1.57E+00	0	Single admission	2
2010	0	1.03E+01	1	2.23E+00	0	Single admission	2
2010	0	1.03E+01	1	2.51E+01	0	Single admission	2
2010	0	1.02E+01	0	1.38E+01	0	Single admission	2
2010	0	1.02E+01	0	1.60E+00	0	Single admission	2
2010	0	1.02E+01	1	1.44E+00	0	Repeated admissions	2
2010	0	1.01E+01	0	4.92E+00	0	Single admission	2
2010	0	1.01E+01	0	7.51E-01	0	Single admission	2
2010	0	1.00E+01	0	7.76E+00	0	Single admission	2
2010	0	9.93E+00	1	2.83E+01	0	Single admission	2
2010	0	9.88E+00	0	1.38E-01	0	Single admission	2
2010	0	9.86E+00	1	1.23E+00	0	Single admission	2
2010	0	9.84E+00	1	1.78E+00	0	Single admission	2
2010	0	6.27E-05	0	9.69E+01	1	Single admission	2
2010	0	9.77E+00	0	7.51E-01	0	Repeated admissions	2
2010	0	9.77E+00	0	9.34E-01	0	Single admission	2
2010	0	5.66E-01	0	2.40E+00	1	Repeated admissions	2
2010	0	9.52E+00	1	1.14E+01	0	Single admission	2
2010	1	1.71E-01	0	3.13E+01	1	Repeated admissions	3
2010	0	1.04E+01	0	1.43E+00	0	Repeated admissions	3
2010	0	1.04E+01	0	8.17E+00	0	Single admission	3
2010	0	3.68E-01	0	6.36E+00	1	Single admission	3
2010	6	1.03E+01	0	3.07E-01	0	Single admission	3

Admission year	Age (years)	Follow up time (years)	Sex (Females=1)	PDRLast	Outcome (Deceased=1)	Single/repeated admission	Diagnostic group
2010	0	1.03E+01	0	9.45E-01	0	Repeated admissions	3
2010	1	1.03E+01	0	5.22E+00	0	Single admission	3
2010	0	1.03E+01	0	8.33E-01	0	Single admission	3
2010	13	1.03E+01	1	1.43E+00	0	Single admission	3
2010	1	1.02E+01	1	2.54E+00	0	Single admission	3
2010	6	1.01E+01	0	7.51E-01	0	Single admission	3
2010	0	1.00E+01	1	3.23E+01	0	Single admission	3
2010	0	9.98E+00	1	2.37E+00	0	Single admission	3
2010	9	9.97E+00	1	8.41E-01	0	Single admission	3
2010	3	9.88E+00	0	1.07E-01	0	Repeated admissions	3
2010	4	2.07E+00	0	1.16E+00	0	Single admission	3
2010	0	9.88E+00	0	5.54E+00	0	Single admission	3
2010	4	9.86E+00	1	5.59E+00	0	Single admission	3
2010	2	9.77E+00	0	3.88E+00	0	Single admission	3
2010	3	9.66E+00	1	1.48E+00	0	Single admission	3
2010	0	9.51E+00	0	6.02E+00	0	Single admission	3
2010	0	9.51E+00	0	2.64E+00	0	Single admission	3
2010	0	1.04E+01	0	7.51E-01	0	Single admission	4
2010	4	1.04E+01	1	1.18E+01	0	Single admission	4
2010	0	1.04E+01	0	4.40E+00	0	Single admission	4
2010	0	1.04E+01	1	5.22E+00	0	Single admission	4
2010	1	1.03E+01	0	2.13E+00	0	Single admission	4
2010	1	1.03E+01	0	1.40E+00	0	Single admission	4
2010	0	1.03E+01	0	6.95E+00	0	Single admission	4
2010	1	1.03E+01	1	1.89E+00	0	Single admission	4
2010	0	1.02E+01	0	7.51E-01	0	Single admission	4
2010	1	1.02E+01	1	5.57E+00	0	Single admission	4
2010	1	1.01E+01	0	1.89E+01	0	Single admission	4
2010	0	1.01E+01	0	2.91E+00	0	Single admission	4
2010	13	1.01E+01	1	9.33E-01	0	Repeated admissions	4

Admission year	Age (years)	Follow up time (years)	Sex (Females=1)	PDRLast	Outcome (Deceased=1)	Single/repeated admission	Diagnostic group
2010	0	1.01E+01	0	2.01E-01	0	Repeated admissions	4
2010	0	1.01E+01	1	1.72E+00	0	Repeated admissions	4
2010	1	1.00E+01	1	8.71E+00	0	Repeated admissions	4
2010	17	1.00E+01	0	1.38E+00	0	Single admission	4
2010	0	3.03E-02	0	6.94E+01	1	Single admission	4
2010	0	9.97E+00	0	2.75E+00	0	Single admission	4
2010	3	9.89E+00	1	1.46E+00	0	Single admission	4
2010	0	9.79E+00	0	2.52E+00	0	Single admission	4
2010	12	9.70E+00	0	1.25E+00	0	Single admission	4
2010	0	9.68E+00	0	1.80E+00	0	Single admission	4
2010	1	9.62E+00	0	1.87E-01	0	Repeated admissions	4
2010	15	9.58E+00	0	1.40E+00	0	Single admission	4
2010	1	9.56E+00	1	4.84E+00	0	Single admission	4
2010	5	9.56E+00	0	1.41E+00	0	Single admission	4
2010	1	9.48E+00	0	1.51E+00	0	Single admission	4
2010	1	2.94E-01	0	4.30E+00	1	Repeated admissions	5
2010	16	2.19E+00	1	1.24E+01	1	Repeated admissions	5
2010	8	2.83E-02	0	2.84E+01	1	Single admission	5
2010	4	1.02E+01	0	1.11E+00	0	Repeated admissions	5
2010	0	1.00E-01	1	3.80E+01	1	Repeated admissions	5
2010	0	1.01E+01	0	7.18E+00	0	Single admission	5
2010	1	1.00E+01	0	1.20E+00	0	Single admission	5
2010	6	9.98E+00	0	2.23E+00	0	Single admission	5
2010	0	9.95E+00	1	7.51E-01	0	Single admission	5
2010	14	9.88E+00	0	1.42E-01	0	Single admission	5
2010	1	9.86E+00	0	1.07E-01	0	Single admission	5
2010	5	9.82E+00	0	1.42E-01	0	Single admission	5
2010	2	9.79E+00	0	2.17E-01	0	Single admission	5
2010	2	9.68E+00	0	5.38E-01	0	Single admission	5
2010	7	4.87E+00	0	1.56E-01	1	Single admission	5

Admission year	Age (years)	Follow up time (years)	Sex (Females=1)	PDRLast	Outcome (Deceased=1)	Single/repeated admission	Diagnostic group
2010	6	9.63E+00	0	1.54E-01	0	Single admission	5
2010	3	9.58E+00	0	1.83E-01	0	Single admission	5
2010	0	9.51E+00	0	2.84E-01	0	Repeated admissions	5
2010	1	1.05E+01	0	3.91E+00	0	Repeated admissions	6
2010	10	1.04E+01	0	7.51E-01	0	Repeated admissions	6
2010	17	1.04E+01	0	8.05E-01	0	Single admission	6
2010	16	3.62E-03	0	1.71E+00	1	Single admission	6
2010	0	1.03E+01	0	1.56E+00	0	Single admission	6
2010	4	1.03E+01	1	9.24E-01	0	Single admission	6
2010	0	1.24E-01	0	6.58E+01	1	Repeated admissions	6
2010	0	1.02E+01	1	4.06E-01	0	Single admission	6
2010	0	1.02E+01	0	7.51E-01	0	Single admission	6
2010	0	1.02E+01	0	1.39E+00	0	Repeated admissions	6
2010	6	1.01E+01	1	1.30E+00	0	Single admission	6
2010	8	1.01E+01	1	5.22E+00	0	Repeated admissions	6
2010	1	1.00E+01	0	7.51E-01	0	Single admission	6
2010	0	2.01E-02	1	8.02E+01	1	Single admission	6
2010	0	9.84E+00	1	6.69E+01	0	Single admission	6
2010	5	9.81E+00	1	1.71E+00	0	Single admission	6
2010	7	9.79E+00	1	1.36E-01	0	Repeated admissions	6
2010	10	9.76E+00	1	4.70E+01	0	Single admission	6
2010	0	1.94E+00	0	1.49E+00	0	Repeated admissions	6
2010	0	9.68E+00	1	1.86E+00	0	Repeated admissions	6
2010	0	5.72E-01	1	1.43E+01	1	Single admission	6
2010	14	7.36E+00	1	1.72E+00	1	Repeated admissions	6
2010	3	9.09E+00	0	1.37E+00	1	Repeated admissions	6
2010	3	9.57E+00	0	1.05E+00	0	Single admission	6
2010	8	9.55E+00	1	9.17E-01	0	Repeated admissions	6
2010	10	9.54E+00	1	1.10E+00	0	Single admission	6
2010	0	9.53E+00	0	1.65E+00	0	Single admission	6

Admission year	Age (years)	Follow up time (years)	Sex (Females=1)	PDRLast	Outcome (Deceased=1)	Single/repeated admission	Diagnostic group
2010	11	9.53E+00	1	1.53E+01	0	Single admission	6
2010	13	9.51E+00	0	2.94E-01	0	Repeated admissions	6
2010	1	1.04E+01	1	3.16E+00	0	Single admission	7
2010	17	1.00E+01	1	1.05E+00	0	Single admission	7
2010	0	1.04E+01	0	4.65E+00	0	Single admission	7
2010	7	1.03E+01	0	1.04E+00	0	Single admission	7
2010	6	8.07E+00	0	2.91E-01	1	Repeated admissions	7
2010	1	1.03E+01	0	1.26E+00	0	Repeated admissions	7
2010	1	1.03E+01	1	6.89E+00	0	Single admission	7
2010	0	1.02E+01	0	3.17E+00	0	Single admission	7
2010	11	1.02E+01	0	3.79E-01	0	Single admission	7
2010	6	1.02E+01	0	1.24E+00	0	Single admission	7
2010	13	1.01E+01	0	2.06E+00	0	Single admission	7
2010	0	1.01E+01	0	2.11E+01	0	Single admission	7
2010	10	1.01E+01	1	7.31E-01	0	Single admission	7
2010	0	1.01E+01	1	1.80E+00	0	Repeated admissions	7
2010	11	1.01E+01	1	1.06E+01	0	Single admission	7
2010	12	1.00E+01	0	1.98E+01	0	Single admission	7
2010	14	9.99E+00	0	9.72E+00	0	Single admission	7
2010	0	9.94E+00	0	1.52E-01	0	Repeated admissions	7
2010	16	9.93E+00	0	1.64E+00	0	Single admission	7
2010	10	9.85E+00	1	1.15E+00	0	Single admission	7
2010	5	9.82E+00	1	5.42E-01	0	Repeated admissions	7
2010	1	9.81E+00	0	1.47E+00	0	Single admission	7
2010	15	9.72E+00	1	8.44E-01	0	Single admission	7
2010	4	9.71E+00	1	1.20E+01	0	Single admission	7
2010	0	8.93E-03	0	3.60E+01	1	Single admission	7
2010	2	9.60E+00	1	1.71E+00	0	Repeated admissions	7
2010	17	9.60E+00	0	7.80E+00	0	Single admission	7
2010	13	9.58E+00	1	3.84E+00	0	Single admission	7

Admission year	Age (years)	Follow up time (years)	Sex (Females=1)	PDRLast	Outcome (Deceased=1)	Single/repeated admission	Diagnostic group
2010	1	9.55E+00	1	2.80E+00	0	Single admission	7
2010	12	7.92E+00	1	4.18E+00	1	Single admission	7
2010	12	9.55E+00	0	2.97E-01	0	Single admission	7
2010	10	9.52E+00	0	6.94E+00	0	Single admission	7
2010	15	9.50E+00	1	4.06E+00	0	Single admission	7
2010	1	7.69E+00	1	2.57E-01	1	Single admission	7
2010	9	1.05E+01	0	8.05E-01	0	Single admission	8
2010	17	1.04E+01	0	3.88E+00	0	Single admission	8
2010	14	1.04E+01	0	8.05E-01	0	Single admission	8
2010	6	1.04E+01	1	9.90E-01	0	Single admission	8
2010	5	1.04E+01	0	1.43E+00	0	Single admission	8
2010	8	1.03E+01	0	7.51E-01	0	Single admission	8
2010	2	1.03E+01	0	3.82E+00	0	Single admission	8
2010	15	1.03E+01	1	2.03E+00	0	Single admission	8
2010	5	1.03E+01	0	1.43E+00	0	Single admission	8
2010	3	1.03E+01	0	8.62E-01	0	Single admission	8
2010	15	1.03E+01	1	9.23E-01	0	Single admission	8
2010	14	1.02E+01	0	6.70E+00	0	Single admission	8
2010	1	8.50E-04	0	4.43E+01	1	Single admission	8
2010	7	1.02E+01	0	7.51E-01	0	Single admission	8
2010	8	1.02E+01	1	2.67E+00	0	Single admission	8
2010	17	6.23E+00	1	9.50E-01	1	Single admission	8
2010	15	1.01E+01	1	4.63E+01	0	Single admission	8
2010	0	1.01E+01	0	1.43E+00	0	Single admission	8
2010	1	1.01E+01	1	1.34E+00	0	Single admission	8
2010	7	1.01E+01	1	8.03E-01	0	Single admission	8
2010	5	1.01E+01	0	7.51E-01	0	Single admission	8
2010	10	1.01E+01	1	1.20E+01	0	Single admission	8
2010	8	1.01E+01	0	1.43E+00	0	Repeated admissions	8
2010	15	1.01E+01	0	8.38E-01	0	Single admission	8

Admission year	Age (years)	Follow up time (years)	Sex (Females=1)	PDRLast	Outcome (Deceased=1)	Single/repeated admission	Diagnostic group
2010	5	1.01E+01	1	5.17E+00	0	Single admission	8
2010	1	1.00E+01	0	1.53E+00	0	Single admission	8
2010	11	1.00E+01	0	7.51E-01	0	Single admission	8
2010	7	1.00E+01	1	1.10E+00	0	Single admission	8
2010	14	1.00E+01	0	8.99E-01	0	Single admission	8
2010	14	1.00E+01	0	3.62E+00	0	Single admission	8
2010	12	9.99E+00	0	3.66E+00	0	Single admission	8
2010	14	9.97E+00	0	8.62E-01	0	Single admission	8
2010	15	9.96E+00	0	1.07E+00	0	Single admission	8
2010	8	9.93E+00	0	7.18E+01	0	Single admission	8
2010	5	9.93E+00	0	7.51E-01	0	Single admission	8
2010	10	9.92E+00	0	6.38E+01	0	Single admission	8
2010	12	9.90E+00	0	7.51E-01	0	Single admission	8
2010	1	9.88E+00	1	5.11E+00	0	Single admission	8
2010	1	9.88E+00	0	1.95E-01	0	Single admission	8
2010	0	3.52E+00	0	4.94E+00	0	Single admission	8
2010	9	9.86E+00	1	7.51E-01	0	Single admission	8
2010	1	9.85E+00	0	6.13E-01	0	Single admission	8
2010	1	9.84E+00	0	1.30E+00	0	Single admission	8
2010	10	9.83E+00	0	1.63E+00	0	Single admission	8
2010	14	9.80E+00	0	7.83E-01	0	Single admission	8
2010	4	1.40E+00	0	5.19E-01	1	Single admission	8
2010	7	9.76E+00	1	8.51E-01	0	Single admission	8
2010	10	9.75E+00	0	1.17E+00	0	Single admission	8
2010	3	9.74E+00	0	1.02E+00	0	Single admission	8
2010	2	9.73E+00	0	1.26E+00	0	Single admission	8
2010	13	9.25E+00	0	8.27E-01	1	Single admission	8
2010	9	9.72E+00	0	9.86E-01	0	Single admission	8
2010	2	9.71E+00	1	4.78E+00	0	Single admission	8
2010	2	9.71E+00	0	7.51E-01	0	Single admission	8

Admission year	Age (years)	Follow up time (years)	Sex (Females=1)	PDRLast	Outcome (Deceased=1)	Single/repeated admission	Diagnostic group
2010	9	9.71E+00	1	1.73E+00	0	Single admission	8
2010	4	9.70E+00	0	4.16E+00	0	Single admission	8
2010	2	9.67E+00	0	1.66E+00	0	Single admission	8
2010	14	9.67E+00	1	3.59E+00	0	Single admission	8
2010	17	9.67E+00	0	9.33E-01	0	Single admission	8
2010	1	9.66E+00	0	1.14E+00	0	Single admission	8
2010	14	9.64E+00	0	8.80E-01	0	Single admission	8
2010	3	9.64E+00	0	1.50E+00	0	Single admission	8
2010	15	9.61E+00	0	1.05E+00	0	Single admission	8
2010	4	9.60E+00	0	2.47E+00	0	Single admission	8
2010	1	9.59E+00	1	1.46E+00	0	Single admission	8
2010	9	9.57E+00	0	1.05E+00	0	Single admission	8
2010	17	9.56E+00	1	9.24E-01	0	Single admission	8
2010	12	9.52E+00	1	7.51E-01	0	Single admission	8
2010	13	9.50E+00	0	1.14E+00	0	Single admission	8
2010	13	8.49E+00	1	9.83E+00	0	Single admission	9
2010	0	1.04E+01	0	1.22E+00	0	Single admission	9
2010	1	1.04E+01	1	7.94E-01	0	Single admission	9
2010	0	1.03E+01	1	3.88E+00	0	Single admission	9
2010	14	1.03E+01	1	1.23E-01	0	Single admission	9
2010	14	1.03E+01	0	1.61E+00	0	Single admission	9
2010	4	1.03E+01	1	1.43E+00	0	Repeated admissions	9
2010	6	1.03E+01	0	5.46E+00	0	Single admission	9
2010	14	1.03E+01	0	9.64E-01	0	Repeated admissions	9
2010	0	1.03E+01	0	3.88E+00	0	Repeated admissions	9
2010	12	1.03E+01	1	1.28E-01	0	Single admission	9
2010	6	1.01E+01	0	1.04E+00	0	Repeated admissions	9
2010	0	1.01E+01	0	1.20E-01	0	Single admission	9
2010	0	1.01E+01	1	1.57E+00	0	Single admission	9
2010	0	1.01E+01	1	1.87E-01	0	Single admission	9

Admission year	Age (years)	Follow up time (years)	Sex (Females=1)	PDRLast	Outcome (Deceased=1)	Single/repeated admission	Diagnostic group
2010	0	1.00E+01	1	5.69E-01	0	Single admission	9
2010	5	9.95E+00	0	5.31E+00	0	Single admission	9
2010	1	9.94E+00	1	8.14E+01	0	Single admission	9
2010	0	1.11E+00	0	6.42E-01	1	Single admission	9
2010	0	9.91E+00	0	9.34E-01	0	Repeated admissions	9
2010	7	4.90E+00	0	1.20E+00	1	Repeated admissions	9
2010	0	9.90E+00	0	1.63E-01	0	Single admission	9
2010	0	9.89E+00	0	1.07E-01	0	Single admission	9
2010	4	9.83E+00	1	2.90E-01	0	Repeated admissions	9
2010	8	9.81E+00	0	1.46E+00	0	Single admission	9
2010	6	9.81E+00	0	3.91E+00	0	Single admission	9
2010	1	9.79E+00	0	1.07E-01	0	Single admission	9
2010	0	9.77E+00	0	5.69E-01	0	Single admission	9
2010	0	9.77E+00	0	1.07E-01	0	Single admission	9
2010	11	9.75E+00	0	3.17E-01	0	Single admission	9
2010	0	9.73E+00	0	5.69E-01	0	Single admission	9
2010	6	9.71E+00	0	1.44E-01	0	Repeated admissions	9
2010	16	9.71E+00	0	6.79E-01	0	Single admission	9
2010	12	9.68E+00	0	7.51E-01	0	Single admission	9
2010	1	9.66E+00	1	1.96E-01	0	Single admission	9
2010	3	9.64E+00	0	5.75E-01	0	Single admission	9
2010	4	9.62E+00	0	1.25E-01	0	Single admission	9
2010	12	9.56E+00	1	8.84E-02	0	Repeated admissions	9
2010	2	9.56E+00	0	1.42E-01	0	Repeated admissions	9
2010	0	9.52E+00	0	5.11E+00	0	Single admission	9
2010	3	1.05E+01	0	9.11E-01	0	Single admission	10
2010	0	1.05E+01	0	7.51E-01	0	Single admission	10
2010	1	1.04E+01	0	3.88E+00	0	Repeated admissions	10
2010	1	1.04E+01	1	8.62E-01	0	Single admission	10
2010	13	1.04E+01	1	1.60E+01	0	Repeated admissions	10

Admission year	Age (years)	Follow up time (years)	Sex (Females=1)	PDRLast	Outcome (Deceased=1)	Single/repeated admission	Diagnostic group
2010	2	1.04E+01	0	6.58E+00	0	Single admission	10
2010	0	1.04E+01	1	8.82E-01	0	Single admission	10
2010	8	1.04E+01	0	7.51E-01	0	Repeated admissions	10
2010	5	1.04E+01	1	1.06E+00	0	Repeated admissions	10
2010	2	1.04E+01	0	1.00E+00	0	Single admission	10
2010	9	1.04E+01	1	9.90E-01	0	Single admission	10
2010	2	1.04E+01	1	1.02E+01	0	Single admission	10
2010	3	1.04E+01	0	8.27E-01	0	Single admission	10
2010	0	1.04E+01	0	2.71E-01	0	Single admission	10
2010	2	1.04E+01	1	6.32E+00	0	Single admission	10
2010	2	1.04E+01	0	4.10E+01	0	Repeated admissions	10
2010	11	1.03E+01	1	8.62E-01	0	Single admission	10
2010	5	1.03E+01	0	3.88E+00	0	Single admission	10
2010	2	1.02E+01	0	7.51E-01	0	Single admission	10
2010	6	5.85E+00	0	7.51E-01	1	Repeated admissions	10
2010	0	3.92E-03	1	9.88E+01	1	Single admission	10
2010	8	1.02E+01	1	7.61E-01	0	Repeated admissions	10
2010	15	1.01E+01	1	1.46E+00	0	Repeated admissions	10
2010	1	1.01E+01	0	1.83E+00	0	Single admission	10
2010	2	1.01E+01	0	7.75E-01	0	Single admission	10
2010	14	1.01E+01	1	2.27E+00	0	Single admission	10
2010	17	1.01E+01	0	1.07E-01	0	Single admission	10
2010	16	1.01E+01	1	1.07E-01	0	Single admission	10
2010	17	1.01E+01	1	5.74E-01	0	Single admission	10
2010	9	1.01E+01	1	1.07E-01	0	Single admission	10
2010	14	1.01E+01	0	7.34E+00	0	Single admission	10
2010	10	1.01E+01	1	5.95E+00	0	Single admission	10
2010	7	1.00E+01	0	1.30E+00	0	Repeated admissions	10
2010	8	1.00E+01	1	1.77E-01	0	Repeated admissions	10
2010	4	1.00E+01	0	7.51E-01	0	Repeated admissions	10

Admission year	Age (years)	Follow up time (years)	Sex (Females=1)	PDRLast	Outcome (Deceased=1)	Single/repeated admission	Diagnostic group
2010	1	9.91E+00	1	2.79E+00	0	Single admission	10
2010	0	9.90E+00	0	4.33E+00	0	Single admission	10
2010	0	9.90E+00	0	4.47E-01	0	Repeated admissions	10
2010	1	9.89E+00	0	1.22E+00	0	Single admission	10
2010	4	6.36E+00	1	1.48E+00	0	Single admission	10
2010	10	9.86E+00	1	7.51E-01	0	Repeated admissions	10
2010	1	9.84E+00	1	9.90E-01	0	Single admission	10
2010	4	9.83E+00	1	8.51E-01	0	Single admission	10
2010	9	8.84E-04	1	9.89E+01	1	Single admission	10
2010	0	9.81E+00	0	4.17E+00	0	Single admission	10
2010	14	9.79E+00	0	5.47E+00	0	Single admission	10
2010	0	6.35E+00	1	1.22E+01	1	Single admission	10
2010	0	9.77E+00	1	2.80E+00	0	Single admission	10
2010	6	9.77E+00	1	1.33E+01	0	Single admission	10
2010	1	9.76E+00	0	4.52E-01	0	Single admission	10
2010	7	9.76E+00	0	1.85E+00	0	Single admission	10
2010	7	5.97E+00	0	3.42E+00	1	Repeated admissions	10
2010	0	9.73E+00	1	1.91E+00	0	Single admission	10
2010	12	9.66E+00	1	1.38E+00	0	Repeated admissions	10
2010	2	9.64E+00	1	1.52E+00	0	Single admission	10
2010	2	3.20E+00	1	2.10E+00	1	Repeated admissions	10
2010	8	9.60E+00	1	8.05E-01	0	Single admission	10
2010	5	1.17E-01	1	8.05E-01	1	Single admission	10
2010	2	3.42E+00	1	8.77E-01	0	Single admission	10
2010	1	9.58E+00	0	1.98E+00	0	Single admission	10
2010	6	9.54E+00	1	1.06E+00	0	Single admission	10
2010	7	9.48E+00	1	8.51E-01	0	Single admission	10
2010	0	1.05E+01	1	1.10E+00	0	Single admission	11
2010	0	1.05E+01	0	2.12E+00	0	Single admission	11
2010	0	1.04E+01	0	4.54E+01	0	Single admission	11

Admission year	Age (years)	Follow up time (years)	Sex (Females=1)	PDRLast	Outcome (Deceased=1)	Single/repeated admission	Diagnostic group
2010	0	1.04E+01	0	2.80E+00	0	Single admission	11
2010	0	1.04E+01	1	5.69E-01	0	Single admission	11
2010	0	5.23E+00	0	7.51E-01	0	Repeated admissions	11
2010	0	1.04E+01	0	2.03E+00	0	Single admission	11
2010	0	1.04E+01	0	1.44E-01	0	Single admission	11
2010	3	1.04E+01	1	7.51E-01	0	Repeated admissions	11
2010	0	1.04E+01	0	5.69E-01	0	Single admission	11
2010	0	3.38E-02	0	1.62E+00	1	Single admission	11
2010	0	1.03E+01	1	8.62E-01	0	Single admission	11
2010	0	1.03E+01	0	3.37E+01	0	Repeated admissions	11
2010	0	5.02E-01	1	6.11E+01	1	Repeated admissions	11
2010	1	1.03E+01	0	2.69E-01	0	Single admission	11
2010	0	1.03E+01	1	5.69E-01	0	Single admission	11
2010	1	2.74E-04	0	1.78E+01	1	Single admission	11
2010	0	1.02E+01	1	3.99E+00	0	Single admission	11
2010	3	1.02E+01	1	1.19E+01	0	Repeated admissions	11
2010	0	1.02E+01	0	1.07E-01	0	Single admission	11
2010	0	1.02E+01	0	5.91E-01	0	Repeated admissions	11
2010	7	1.02E+01	0	8.60E-01	0	Single admission	11
2010	0	1.02E+01	0	4.79E-01	0	Single admission	11
2010	9	1.01E+01	1	1.63E-01	0	Single admission	11
2010	0	1.01E+01	0	9.24E-01	0	Single admission	11
2010	0	5.03E-02	1	3.91E+00	1	Single admission	11
2010	1	8.39E-01	1	7.08E-01	1	Repeated admissions	11
2010	9	1.01E+01	1	3.98E-01	0	Single admission	11
2010	0	1.01E+01	1	1.84E+00	0	Repeated admissions	11
2010	1	7.58E+00	0	2.80E+00	0	Repeated admissions	11
2010	0	1.01E+01	1	1.05E+00	0	Single admission	11
2010	0	6.98E-02	1	3.56E+01	1	Single admission	11
2010	0	1.00E+01	1	5.43E-01	0	Single admission	11

Admission year	Age (years)	Follow up time (years)	Sex (Females=1)	PDRLast	Outcome (Deceased=1)	Single/repeated admission	Diagnostic group
2010	2	1.00E+01	1	2.13E-01	0	Single admission	11
2010	6	5.80E+00	0	9.63E-01	1	Repeated admissions	11
2010	0	1.00E+01	0	3.99E+00	0	Single admission	11
2010	1	1.00E+01	0	2.13E-01	0	Single admission	11
2010	0	1.00E+01	0	6.77E+00	0	Single admission	11
2010	0	1.00E+01	0	9.29E+00	0	Repeated admissions	11
2010	6	1.00E+01	0	5.21E-01	0	Single admission	11
2010	0	9.16E-03	0	5.47E+00	1	Single admission	11
2010	0	9.96E+00	0	1.75E-01	0	Repeated admissions	11
2010	0	9.95E+00	0	4.77E+00	0	Single admission	11
2010	0	9.94E+00	1	3.76E+00	0	Single admission	11
2010	0	9.93E+00	0	6.88E-01	0	Repeated admissions	11
2010	0	9.90E+00	1	7.51E-01	0	Single admission	11
2010	0	9.88E+00	1	7.51E-01	0	Single admission	11
2010	0	9.86E+00	0	1.20E-01	0	Single admission	11
2010	0	9.86E+00	0	2.44E+00	0	Single admission	11
2010	0	9.85E+00	0	5.44E+00	0	Single admission	11
2010	0	9.83E+00	1	1.07E-01	0	Single admission	11
2010	12	9.83E+00	1	1.19E-01	0	Single admission	11
2010	0	9.80E+00	0	1.56E+00	0	Single admission	11
2010	0	9.78E+00	0	1.90E+00	0	Single admission	11
2010	4	9.78E+00	1	1.58E-01	0	Repeated admissions	11
2010	0	9.77E+00	0	5.69E-01	0	Single admission	11
2010	0	9.77E+00	0	5.69E-01	0	Single admission	11
2010	1	9.77E+00	1	2.84E-01	0	Single admission	11
2010	0	9.76E+00	1	6.23E-01	0	Single admission	11
2010	4	9.74E+00	1	1.56E-01	0	Repeated admissions	11
2010	0	3.90E-03	1	4.52E+01	1	Single admission	11
2010	0	9.72E+00	1	2.11E+00	0	Single admission	11
2010	0	9.70E+00	0	7.51E-01	0	Single admission	11

Admission year	Age (years)	Follow up time (years)	Sex (Females=1)	PDRLast	Outcome (Deceased=1)	Single/repeated admission	Diagnostic group
2010	0	3.35E-02	1	8.38E+00	1	Single admission	11
2010	0	9.69E+00	0	1.56E-01	0	Single admission	11
2010	13	9.69E+00	1	6.22E+00	0	Repeated admissions	11
2010	1	9.65E+00	0	1.85E+00	0	Repeated admissions	11
2010	0	1.22E-01	1	7.56E+01	1	Repeated admissions	11
2010	0	9.63E+00	1	2.43E-01	0	Single admission	11
2010	14	9.63E+00	1	1.75E-01	0	Single admission	11
2010	0	9.62E+00	1	4.14E-01	0	Single admission	11
2010	0	9.61E+00	1	6.31E-01	0	Single admission	11
2010	0	9.61E+00	1	9.45E-01	0	Single admission	11
2010	1	3.72E+00	0	7.51E-01	0	Repeated admissions	11
2010	0	9.61E+00	0	7.51E-01	0	Single admission	11
2010	0	9.59E+00	0	2.12E+00	0	Single admission	11
2010	0	9.59E+00	0	1.19E+00	0	Repeated admissions	11
2010	5	6.48E+00	1	3.51E+00	0	Repeated admissions	11
2010	3	9.57E+00	1	2.22E-02	0	Repeated admissions	11
2010	0	9.56E+00	0	1.75E-01	0	Repeated admissions	11
2010	1	9.56E+00	0	1.07E+00	0	Single admission	11
2010	0	9.54E+00	0	1.77E+00	0	Single admission	11
2010	0	9.54E+00	0	1.72E+00	0	Single admission	11
2010	0	9.54E+00	1	2.36E+00	0	Single admission	11
2010	5	9.52E+00	0	9.06E-01	0	Repeated admissions	11
2010	5	9.51E+00	0	2.08E-01	0	Repeated admissions	11
2010	2	1.05E+01	0	1.78E+01	0	Repeated admissions	12
2010	0	1.05E+01	0	7.51E-01	0	Repeated admissions	12
2010	2	1.05E+01	1	1.56E-01	0	Single admission	12
2010	4	1.04E+01	1	1.07E-01	0	Repeated admissions	12
2010	3	1.04E+01	1	3.07E+00	0	Repeated admissions	12
2010	0	1.04E+01	0	1.94E+00	0	Single admission	12
2010	0	1.04E+01	0	3.62E+01	0	Single admission	12

Admission year	Age (years)	Follow up time (years)	Sex (Females=1)	PDRLast	Outcome (Deceased=1)	Single/repeated admission	Diagnostic group
2010	8	1.04E+01	0	1.51E+01	0	Single admission	12
2010	0	1.04E+01	1	3.88E+00	0	Repeated admissions	12
2010	5	2.25E+00	1	6.02E+00	1	Repeated admissions	12
2010	0	1.04E+01	1	2.00E-01	0	Single admission	12
2010	2	1.04E+01	0	1.54E-01	0	Repeated admissions	12
2010	0	1.04E+01	0	8.77E+00	0	Single admission	12
2010	6	1.04E+01	1	8.27E-01	0	Single admission	12
2010	14	1.04E+01	1	3.88E+00	0	Single admission	12
2010	2	1.04E+01	1	8.27E-01	0	Single admission	12
2010	1	1.04E+01	1	5.91E-01	0	Single admission	12
2010	0	1.04E+01	1	5.91E-01	0	Repeated admissions	12
2010	0	1.04E+01	1	7.51E-01	0	Repeated admissions	12
2010	2	1.04E+01	1	3.88E+00	0	Single admission	12
2010	0	1.04E+01	0	7.02E+00	0	Single admission	12
2010	1	1.32E+00	1	3.64E+00	1	Repeated admissions	12
2010	2	1.04E+01	0	3.85E+01	0	Repeated admissions	12
2010	1	1.04E+01	1	1.32E+00	0	Single admission	12
2010	0	1.04E+01	0	5.91E-01	0	Repeated admissions	12
2010	2	1.04E+01	0	8.39E-01	0	Repeated admissions	12
2010	0	1.04E+01	1	6.45E-01	0	Repeated admissions	12
2010	0	1.04E+01	0	7.51E-01	0	Single admission	12
2010	0	1.03E+01	0	1.56E+01	0	Single admission	12
2010	2	1.03E+01	0	1.12E+01	0	Single admission	12
2010	0	1.03E+01	0	7.51E-01	0	Single admission	12
2010	0	1.03E+01	1	1.56E-01	0	Single admission	12
2010	9	1.03E+01	0	1.56E-01	0	Single admission	12
2010	10	1.03E+01	0	7.51E-01	0	Single admission	12
2010	5	1.03E+01	0	2.80E+00	0	Repeated admissions	12
2010	0	1.03E+01	1	1.56E-01	0	Single admission	12
2010	0	1.03E+01	1	4.43E-01	0	Single admission	12

Admission year	Age (years)	Follow up time (years)	Sex (Females=1)	PDRLast	Outcome (Deceased=1)	Single/repeated admission	Diagnostic group
2010	5	1.03E+01	1	3.12E+00	0	Single admission	12
2010	0	1.03E+01	0	1.56E-01	0	Repeated admissions	12
2010	0	1.03E+01	0	5.91E-01	0	Repeated admissions	12
2010	0	1.03E+01	1	8.27E-01	0	Single admission	12
2010	0	1.03E+01	0	3.88E+00	0	Single admission	12
2010	0	1.03E+01	0	1.56E-01	0	Single admission	12
2010	0	1.03E+01	1	3.07E+00	0	Single admission	12
2010	1	1.03E+01	1	2.31E+01	0	Single admission	12
2010	0	1.03E+01	1	1.02E+00	0	Single admission	12
2010	0	1.03E+01	1	3.95E+00	0	Single admission	12
2010	0	1.03E+01	0	5.82E-01	0	Single admission	12
2010	0	1.03E+01	0	5.91E-01	0	Single admission	12
2010	1	1.03E+01	0	3.88E+00	0	Single admission	12
2010	0	1.03E+01	0	7.81E-01	0	Single admission	12
2010	0	1.03E+01	0	1.63E-01	0	Single admission	12
2010	0	1.03E+01	0	8.27E-01	0	Single admission	12
2010	1	1.03E+01	0	8.27E-01	0	Repeated admissions	12
2010	0	1.03E+01	0	8.27E-01	0	Repeated admissions	12
2010	0	1.03E+01	1	7.65E-01	0	Repeated admissions	12
2010	0	1.03E+01	1	8.35E-01	0	Single admission	12
2010	0	1.03E+01	0	4.24E+00	0	Repeated admissions	12
2010	0	1.03E+01	0	3.03E+00	0	Single admission	12
2010	0	1.03E+01	0	5.91E-01	0	Repeated admissions	12
2010	2	1.03E+01	1	2.64E-01	0	Single admission	12
2010	0	1.56E+00	0	3.88E+00	0	Repeated admissions	12
2010	0	1.02E+01	0	1.56E-01	0	Repeated admissions	12
2010	0	1.02E+01	0	7.51E-01	0	Single admission	12
2010	1	1.02E+01	0	4.30E+00	0	Repeated admissions	12
2010	2	1.02E+01	0	6.21E+00	0	Single admission	12
2010	2	1.02E+01	0	4.49E+00	0	Single admission	12

Admission year	Age (years)	Follow up time (years)	Sex (Females=1)	PDRLast	Outcome (Deceased=1)	Single/repeated admission	Diagnostic group
2010	0	1.02E+01	0	3.88E+00	0	Single admission	12
2010	0	1.02E+01	0	3.07E+00	0	Repeated admissions	12
2010	0	1.02E+01	0	4.35E+01	0	Single admission	12
2010	0	1.02E+01	0	1.14E+01	0	Repeated admissions	12
2010	0	1.02E+01	1	1.56E-01	0	Single admission	12
2010	0	1.02E+01	0	3.88E+00	0	Single admission	12
2010	13	1.01E+01	0	7.51E-01	0	Repeated admissions	12
2010	13	6.30E-02	0	1.80E+01	1	Single admission	12
2010	2	1.01E+01	0	8.68E-01	0	Repeated admissions	12
2010	1	1.01E+01	0	8.27E-01	0	Single admission	12
2010	0	1.01E+01	0	9.26E-01	0	Repeated admissions	12
2010	1	1.01E+01	1	2.91E-01	0	Single admission	12
2010	0	1.01E+01	0	5.80E+00	0	Single admission	12
2010	1	1.01E+01	0	4.10E+00	0	Single admission	12
2010	1	1.00E+01	1	2.01E-01	0	Single admission	12
2010	0	1.00E+01	1	1.14E+00	0	Single admission	12
2010	1	1.00E+01	0	1.33E+01	0	Single admission	12
2010	1	1.00E+01	1	2.74E-01	0	Single admission	12
2010	2	1.00E+01	0	8.36E+00	0	Single admission	12
2010	1	1.00E+01	0	3.88E+00	0	Single admission	12
2010	11	4.96E+00	0	4.29E+00	1	Single admission	12
2010	0	9.85E+00	0	7.77E+01	0	Single admission	12
2010	1	9.83E+00	0	6.05E+00	0	Single admission	12
2010	18	2.92E+00	0	7.11E+00	1	Repeated admissions	12
2010	5	1.88E+00	1	1.47E-01	1	Repeated admissions	12
2010	4	9.78E+00	1	7.51E-01	0	Single admission	12
2010	2	9.77E+00	1	3.88E+00	0	Single admission	12
2010	0	9.76E+00	1	6.26E-01	0	Single admission	12
2010	0	5.20E+00	0	1.43E+00	0	Single admission	12
2010	1	9.72E+00	1	2.86E+00	0	Single admission	12

Admission year	Age (years)	Follow up time (years)	Sex (Females=1)	PDRLast	Outcome (Deceased=1)	Single/repeated admission	Diagnostic group
2010	13	9.72E+00	0	6.26E+00	0	Single admission	12
2010	1	5.37E+00	0	1.54E+00	0	Single admission	12
2010	0	9.65E+00	1	5.39E+00	0	Single admission	12
2010	0	9.64E+00	0	1.76E+00	0	Repeated admissions	12
2010	0	9.61E+00	0	1.07E-01	0	Single admission	12
2010	0	9.61E+00	1	6.18E+00	0	Single admission	12
2010	0	9.60E+00	0	2.24E+00	0	Repeated admissions	12
2010	1	9.59E+00	0	4.49E+00	0	Single admission	12
2010	2	1.44E+00	1	7.83E-01	0	Single admission	12
2010	1	9.56E+00	0	5.23E+00	0	Single admission	12
2010	0	9.54E+00	1	2.24E-01	0	Single admission	12
2010	5	9.54E+00	1	1.88E+01	0	Repeated admissions	12
2010	5	9.53E+00	0	5.74E+00	0	Repeated admissions	12
2010	2	9.52E+00	0	2.10E+00	0	Single admission	12
2010	0	9.52E+00	0	2.71E-01	0	Single admission	12
2010	1	5.86E-01	0	3.32E+01	1	Repeated admissions	12
2010	0	9.51E+00	0	9.98E-01	0	Single admission	12
2010	0	9.51E+00	1	5.91E-01	0	Single admission	12
2010	0	9.50E+00	0	1.65E+00	0	Single admission	12
2010	1	9.49E+00	0	9.09E-01	0	Single admission	12
2010	0	9.49E+00	0	9.99E-01	0	Single admission	12
2010	10	9.48E+00	1	9.90E-01	0	Single admission	12
2010	0	2.20E-01	0	1.47E+00	0	Repeated admissions	12
2011	0	9.47E+00	0	2.57E+00	0	Single admission	1
2011	1	1.03E+00	0	7.98E+00	1	Repeated admissions	1
2011	1	9.04E+00	1	1.02E+01	0	Single admission	1
2011	2	9.03E+00	1	3.67E-01	0	Repeated admissions	1
2011	10	8.97E+00	1	1.09E+01	0	Single admission	1
2011	0	8.82E+00	0	2.15E-01	0	Single admission	1
2011	1	8.73E+00	0	3.18E+00	0	Single admission	1

Admission year	Age (years)	Follow up time (years)	Sex (Females=1)	PDRLast	Outcome (Deceased=1)	Single/repeated admission	Diagnostic group
2011	10	8.59E+00	1	7.61E-01	0	Single admission	1
2011	0	9.41E+00	0	1.25E+00	0	Single admission	2
2011	0	9.38E+00	0	2.47E-01	0	Single admission	2
2011	5	9.35E+00	1	1.11E+00	0	Repeated admissions	2
2011	0	9.26E+00	1	2.71E-01	0	Repeated admissions	2
2011	0	9.20E+00	0	4.87E-01	0	Repeated admissions	2
2011	0	9.19E+00	1	1.22E+00	0	Single admission	2
2011	0	9.19E+00	0	1.30E+00	0	Single admission	2
2011	0	3.27E-02	0	6.52E+01	1	Single admission	2
2011	0	9.19E+00	0	1.30E+00	0	Single admission	2
2011	0	9.15E+00	1	4.39E+00	0	Single admission	2
2011	0	9.08E+00	0	3.14E+01	0	Single admission	2
2011	1	9.06E+00	0	7.83E-01	0	Single admission	2
2011	0	9.06E+00	1	7.51E-01	0	Single admission	2
2011	0	8.98E+00	0	2.19E+00	0	Single admission	2
2011	1	8.92E+00	1	2.16E+01	0	Single admission	2
2011	0	8.69E+00	0	9.13E+00	0	Single admission	2
2011	0	8.69E+00	0	5.22E+00	0	Single admission	2
2011	0	8.69E+00	1	7.09E+00	0	Single admission	2
2011	0	8.56E+00	0	3.88E+00	0	Single admission	2
2011	0	9.46E+00	1	1.48E+00	0	Single admission	3
2011	0	3.07E-03	0	4.72E+00	1	Single admission	3
2011	14	9.42E+00	1	3.48E-01	0	Single admission	3
2011	14	9.30E+00	1	1.62E+00	0	Single admission	3
2011	14	9.27E+00	0	9.77E-01	0	Repeated admissions	3
2011	5	9.27E+00	1	1.34E+00	0	Single admission	3
2011	2	9.27E+00	1	3.04E+00	0	Single admission	3
2011	0	9.25E+00	0	1.26E+01	0	Repeated admissions	3
2011	3	9.25E+00	0	4.11E-01	0	Repeated admissions	3
2011	0	9.24E+00	0	6.12E+00	0	Single admission	3

Admission year	Age (years)	Follow up time (years)	Sex (Females=1)	PDRLast	Outcome (Deceased=1)	Single/repeated admission	Diagnostic group
2011	0	9.22E+00	1	2.80E+00	0	Single admission	3
2011	0	8.86E+00	1	9.07E-01	0	Single admission	3
2011	0	8.85E+00	0	1.52E+00	0	Single admission	3
2011	0	5.81E-02	0	2.29E+01	1	Repeated admissions	3
2011	3	8.60E+00	1	5.20E+00	0	Single admission	3
2011	2	8.60E+00	0	3.15E+00	0	Repeated admissions	3
2011	0	8.56E+00	1	2.67E-01	0	Single admission	3
2011	1	8.51E+00	0	2.44E+01	0	Repeated admissions	3
2011	1	9.46E+00	1	2.29E+00	0	Single admission	4
2011	15	5.07E-02	0	2.80E+01	1	Single admission	4
2011	0	9.31E+00	1	6.32E+00	0	Single admission	4
2011	0	9.23E+00	1	7.51E-01	0	Single admission	4
2011	18	9.16E+00	0	2.33E+00	0	Single admission	4
2011	5	6.20E-04	0	9.39E+01	1	Single admission	4
2011	10	9.09E+00	1	2.49E+00	0	Single admission	4
2011	3	9.07E+00	0	7.08E+00	0	Single admission	4
2011	2	9.06E+00	1	1.56E+00	0	Single admission	4
2011	5	9.06E+00	0	1.05E+00	0	Repeated admissions	4
2011	1	9.03E+00	0	7.54E-01	0	Repeated admissions	4
2011	1	8.98E+00	1	8.51E-01	0	Single admission	4
2011	14	8.95E+00	1	1.04E+00	0	Single admission	4
2011	4	8.90E+00	1	4.53E+00	0	Single admission	4
2011	0	8.89E+00	0	1.08E+01	0	Single admission	4
2011	0	8.79E+00	1	1.12E+00	0	Repeated admissions	4
2011	0	8.77E+00	1	2.49E+00	0	Repeated admissions	4
2011	15	8.74E+00	0	1.87E+00	0	Single admission	4
2011	2	1.19E+00	0	3.18E+00	1	Repeated admissions	4
2011	1	8.69E+00	0	1.35E+00	0	Single admission	4
2011	1	8.64E+00	1	3.74E+01	0	Single admission	4
2011	7	8.53E+00	1	2.24E+01	0	Single admission	4

Admission year	Age (years)	Follow up time (years)	Sex (Females=1)	PDRLast	Outcome (Deceased=1)	Single/repeated admission	Diagnostic group
2011	3	2.22E+00	1	1.47E+01	1	Repeated admissions	5
2011	10	9.45E+00	0	4.92E+00	0	Repeated admissions	5
2011	1	4.64E-01	0	2.29E+01	1	Single admission	5
2011	9	1.04E+00	0	5.04E+01	1	Repeated admissions	5
2011	11	9.39E+00	1	7.34E-01	0	Single admission	5
2011	0	6.50E-03	0	4.65E+00	1	Single admission	5
2011	3	3.30E+00	1	7.96E-01	1	Single admission	5
2011	1	9.33E+00	0	2.99E+00	0	Single admission	5
2011	5	9.33E+00	0	3.29E-01	0	Repeated admissions	5
2011	14	9.30E+00	0	3.36E-01	0	Single admission	5
2011	0	9.25E+00	1	5.69E-01	0	Single admission	5
2011	1	9.25E+00	0	2.49E-01	0	Single admission	5
2011	8	9.20E+00	0	8.64E-01	0	Single admission	5
2011	0	9.17E+00	1	5.14E+00	0	Single admission	5
2011	3	9.08E+00	0	5.29E-01	0	Single admission	5
2011	3	8.99E+00	1	1.96E-01	0	Single admission	5
2011	0	8.94E+00	1	4.84E-01	0	Single admission	5
2011	15	8.85E+00	1	1.54E-01	0	Single admission	5
2011	9	8.83E+00	1	1.14E-01	0	Single admission	5
2011	9	5.09E-02	0	2.96E+00	1	Single admission	5
2011	1	8.78E+00	0	3.30E+00	0	Single admission	5
2011	6	8.74E+00	0	2.04E-01	0	Single admission	5
2011	5	8.69E+00	1	3.68E+00	0	Single admission	5
2011	9	8.49E+00	0	1.78E+01	0	Single admission	5
2011	3	9.40E+00	0	9.43E-01	0	Repeated admissions	6
2011	0	9.68E-02	1	7.51E-01	1	Repeated admissions	6
2011	0	9.31E+00	0	8.05E-01	0	Single admission	6
2011	0	7.45E-02	1	1.09E+00	1	Single admission	6
2011	11	2.74E-04	1	9.94E+01	1	Single admission	6
2011	0	9.28E+00	0	3.55E+01	0	Single admission	6

Admission year	Age (years)	Follow up time (years)	Sex (Females=1)	PDRLast	Outcome (Deceased=1)	Single/repeated admission	Diagnostic group
2011	1	9.26E+00	1	8.80E+01	0	Single admission	6
2011	1	9.22E+00	1	1.93E+01	0	Single admission	6
2011	0	9.19E+00	1	1.24E+01	0	Single admission	6
2011	1	9.16E+00	0	1.89E+00	0	Single admission	6
2011	1	9.14E+00	0	5.58E+01	0	Single admission	6
2011	1	9.09E+00	1	3.08E+01	0	Single admission	6
2011	0	9.04E+00	0	3.56E+01	0	Single admission	6
2011	0	9.01E+00	1	1.30E+01	0	Single admission	6
2011	0	9.00E+00	1	2.52E+00	0	Single admission	6
2011	1	8.96E+00	0	8.43E-01	0	Repeated admissions	6
2011	1	8.94E+00	0	1.77E+00	0	Single admission	6
2011	14	8.94E+00	0	6.55E+00	0	Repeated admissions	6
2011	1	2.22E-01	1	5.68E+00	1	Repeated admissions	6
2011	0	8.91E+00	0	1.10E+00	0	Single admission	6
2011	16	4.04E+00	1	4.53E-01	1	Repeated admissions	6
2011	2	8.89E+00	0	4.84E+00	0	Repeated admissions	6
2011	0	8.85E+00	1	4.86E+01	0	Single admission	6
2011	14	8.85E+00	1	1.67E+00	0	Repeated admissions	6
2011	0	8.84E+00	1	1.12E+01	0	Single admission	6
2011	14	8.84E+00	0	1.72E+00	0	Single admission	6
2011	16	8.82E+00	1	3.78E-01	0	Single admission	6
2011	0	8.72E+00	1	3.76E+00	0	Single admission	6
2011	16	8.66E+00	1	1.30E+00	0	Single admission	6
2011	0	8.66E+00	0	7.42E+01	0	Single admission	6
2011	6	8.62E+00	1	4.47E+00	0	Single admission	6
2011	0	8.61E+00	1	1.54E+00	0	Single admission	6
2011	1	8.57E+00	1	7.51E-01	0	Single admission	6
2011	0	6.05E-02	0	1.33E+01	1	Single admission	6
2011	9	8.50E+00	1	3.91E+00	0	Single admission	6
2011	0	8.50E+00	1	3.17E+00	0	Single admission	6

Admission year	Age (years)	Follow up time (years)	Sex (Females=1)	PDRLast	Outcome (Deceased=1)	Single/repeated admission	Diagnostic group
2011	0	8.49E+00	0	5.04E+00	0	Single admission	6
2011	8	8.49E+00	1	3.42E+00	0	Single admission	6
2011	4	8.48E+00	0	9.28E+00	0	Single admission	6
2011	0	9.46E+00	0	2.03E+00	0	Single admission	7
2011	0	9.43E+00	0	6.72E+00	0	Repeated admissions	7
2011	1	9.41E+00	1	1.59E+01	0	Single admission	7
2011	10	9.40E+00	0	1.13E+00	0	Single admission	7
2011	7	9.39E+00	1	6.44E-01	0	Single admission	7
2011	8	9.37E+00	0	9.93E-01	0	Single admission	7
2011	13	9.37E+00	1	9.75E-01	0	Single admission	7
2011	8	9.36E+00	0	1.06E+00	0	Single admission	7
2011	16	9.35E+00	0	1.24E+00	0	Single admission	7
2011	11	9.31E+00	0	3.90E+01	0	Single admission	7
2011	1	9.29E+00	0	1.21E+00	0	Single admission	7
2011	9	9.26E+00	0	1.56E+00	0	Single admission	7
2011	0	4.66E-02	0	1.13E+00	1	Single admission	7
2011	8	9.21E+00	0	5.42E-01	0	Single admission	7
2011	17	9.18E+00	1	2.06E+00	0	Repeated admissions	7
2011	11	9.17E+00	1	9.09E-01	0	Single admission	7
2011	0	9.10E+00	0	6.00E+00	0	Single admission	7
2011	0	9.10E+00	0	5.69E-01	0	Single admission	7
2011	0	9.07E+00	0	3.88E+00	0	Single admission	7
2011	1	1.20E+00	1	1.60E+00	1	Repeated admissions	7
2011	0	8.96E+00	0	1.23E+01	0	Single admission	7
2011	0	8.92E+00	0	1.34E+01	0	Single admission	7
2011	3	8.89E+00	1	8.62E-01	0	Single admission	7
2011	7	8.88E+00	1	1.39E+01	0	Single admission	7
2011	2	8.84E+00	0	7.60E-01	0	Single admission	7
2011	14	8.83E+00	0	4.63E+00	0	Single admission	7
2011	13	8.83E+00	1	8.39E-01	0	Single admission	7

Admission year	Age (years)	Follow up time (years)	Sex (Females=1)	PDRLast	Outcome (Deceased=1)	Single/repeated admission	Diagnostic group
2011	1	8.78E+00	1	1.10E+01	0	Single admission	7
2011	0	2.32E+00	0	1.73E+00	0	Single admission	7
2011	18	8.76E+00	1	9.90E-01	0	Single admission	7
2011	14	8.69E+00	0	6.69E+00	0	Single admission	7
2011	1	8.68E+00	0	9.14E+00	0	Single admission	7
2011	3	8.66E+00	1	3.95E+00	0	Single admission	7
2011	3	8.54E+00	1	2.48E+00	0	Single admission	7
2011	14	8.53E+00	0	4.93E+00	0	Single admission	7
2011	11	8.51E+00	0	1.49E+00	0	Single admission	7
2011	12	8.49E+00	1	5.94E+00	0	Single admission	7
2011	14	8.48E+00	0	3.56E+00	0	Single admission	7
2011	5	9.46E+00	1	3.88E+00	0	Single admission	8
2011	15	9.45E+00	0	1.44E+00	0	Single admission	8
2011	5	9.44E+00	0	7.23E+00	0	Single admission	8
2011	13	9.43E+00	0	8.95E-01	0	Single admission	8
2011	4	9.42E+00	0	8.62E-01	0	Single admission	8
2011	1	9.38E+00	0	4.41E-01	0	Single admission	8
2011	3	9.31E+00	1	5.21E+00	0	Single admission	8
2011	18	9.26E+00	0	9.90E-01	0	Single admission	8
2011	5	9.23E+00	0	4.36E+00	0	Repeated admissions	8
2011	17	9.22E+00	1	9.13E-01	0	Single admission	8
2011	8	9.19E+00	1	7.72E-01	0	Single admission	8
2011	0	9.17E+00	1	5.34E+00	0	Single admission	8
2011	13	9.16E+00	1	1.08E+00	0	Single admission	8
2011	13	9.14E+00	0	9.90E-01	0	Single admission	8
2011	15	9.12E+00	1	1.31E+00	0	Single admission	8
2011	3	9.12E+00	0	8.16E-01	0	Single admission	8
2011	18	9.11E+00	1	7.51E-01	0	Single admission	8
2011	1	9.09E+00	0	8.62E-01	0	Single admission	8
2011	12	9.08E+00	0	4.08E+00	0	Single admission	8

Admission year	Age (years)	Follow up time (years)	Sex (Females=1)	PDRLast	Outcome (Deceased=1)	Single/repeated admission	Diagnostic group
2011	10	9.07E+00	0	3.54E+00	0	Single admission	8
2011	15	9.03E+00	0	9.85E-01	0	Single admission	8
2011	2	9.03E+00	1	1.11E+00	0	Single admission	8
2011	5	9.02E+00	1	1.04E+00	0	Single admission	8
2011	8	9.02E+00	1	9.98E-01	0	Single admission	8
2011	13	8.40E+00	1	7.61E-01	0	Single admission	8
2011	2	9.01E+00	0	7.51E-01	0	Single admission	8
2011	8	9.00E+00	0	1.56E+00	0	Single admission	8
2011	17	8.99E+00	1	9.90E-01	0	Single admission	8
2011	0	5.06E-03	0	5.97E+01	1	Single admission	8
2011	14	8.98E+00	0	1.30E+00	0	Repeated admissions	8
2011	13	8.98E+00	0	3.43E+00	0	Single admission	8
2011	1	8.97E+00	0	3.90E+00	0	Single admission	8
2011	4	8.95E+00	0	1.53E+00	0	Single admission	8
2011	1	8.92E+00	0	7.00E+00	0	Single admission	8
2011	6	8.91E+00	0	9.11E-01	0	Single admission	8
2011	15	8.91E+00	0	9.90E-01	0	Single admission	8
2011	7	8.91E+00	1	4.21E+00	0	Single admission	8
2011	2	8.90E+00	0	1.02E+00	0	Single admission	8
2011	8	8.83E+00	0	2.23E-01	0	Repeated admissions	8
2011	13	8.82E+00	0	8.33E-01	0	Single admission	8
2011	9	8.82E+00	0	4.07E+00	0	Single admission	8
2011	6	8.78E+00	0	8.62E-01	0	Single admission	8
2011	7	8.78E+00	0	9.24E-01	0	Single admission	8
2011	0	8.76E+00	1	7.51E-01	0	Single admission	8
2011	2	8.74E+00	0	3.46E+01	0	Single admission	8
2011	1	8.74E+00	0	3.88E+00	0	Single admission	8
2011	6	8.72E+00	0	9.37E-01	0	Single admission	8
2011	13	8.72E+00	0	8.16E-01	0	Single admission	8
2011	0	8.71E+00	1	1.44E+00	0	Single admission	8

Admission year	Age (years)	Follow up time (years)	Sex (Females=1)	PDRLast	Outcome (Deceased=1)	Single/repeated admission	Diagnostic group
2011	15	8.71E+00	0	9.96E-01	0	Single admission	8
2011	6	8.70E+00	0	3.38E-01	0	Single admission	8
2011	12	8.69E+00	0	1.13E+00	0	Single admission	8
2011	1	8.69E+00	0	4.28E+00	0	Single admission	8
2011	1	8.69E+00	1	1.33E+01	0	Single admission	8
2011	1	8.67E+00	0	1.06E+00	0	Single admission	8
2011	15	8.66E+00	1	1.21E+00	0	Single admission	8
2011	10	8.65E+00	0	7.83E-01	0	Single admission	8
2011	18	8.65E+00	0	1.02E+00	0	Repeated admissions	8
2011	4	8.61E+00	1	9.77E-01	0	Single admission	8
2011	13	8.56E+00	1	1.24E+00	0	Single admission	8
2011	1	8.55E+00	1	2.80E+00	0	Single admission	8
2011	14	9.37E+00	0	8.96E-01	0	Single admission	9
2011	0	9.35E+00	0	1.32E-01	0	Repeated admissions	9
2011	14	9.33E+00	0	1.29E-01	0	Single admission	9
2011	8	9.21E+00	1	1.01E+00	0	Single admission	9
2011	5	9.19E+00	1	9.72E-01	0	Single admission	9
2011	0	8.45E+00	0	4.78E+00	0	Single admission	9
2011	0	9.15E+00	1	1.03E+01	0	Single admission	9
2011	13	9.15E+00	0	1.38E-01	0	Single admission	9
2011	0	9.15E+00	0	3.05E+01	0	Single admission	9
2011	13	9.14E+00	0	1.92E-01	0	Single admission	9
2011	0	9.14E+00	1	1.43E+00	0	Single admission	9
2011	0	9.14E+00	0	6.40E-01	0	Single admission	9
2011	0	9.13E+00	0	1.43E+00	0	Single admission	9
2011	12	9.12E+00	1	1.93E-01	0	Single admission	9
2011	11	9.10E+00	1	3.90E-01	0	Single admission	9
2011	13	9.08E+00	1	1.48E-01	0	Single admission	9
2011	0	9.07E+00	0	2.21E-01	0	Single admission	9
2011	0	9.06E+00	1	6.59E-01	0	Single admission	9

Admission year	Age (years)	Follow up time (years)	Sex (Females=1)	PDRLast	Outcome (Deceased=1)	Single/repeated admission	Diagnostic group
2011	0	9.06E+00	0	1.63E+00	0	Single admission	9
2011	1	9.04E+00	0	5.37E-01	0	Single admission	9
2011	1	9.02E+00	1	1.48E+01	0	Repeated admissions	9
2011	0	9.01E+00	0	5.82E-01	0	Single admission	9
2011	2	9.00E+00	1	7.23E+00	0	Repeated admissions	9
2011	2	8.99E+00	1	3.00E+00	0	Repeated admissions	9
2011	1	8.98E+00	0	5.69E-01	0	Single admission	9
2011	6	3.00E+00	1	9.39E-01	1	Single admission	9
2011	0	8.93E+00	0	2.33E-01	0	Single admission	9
2011	0	8.93E+00	0	1.77E-01	0	Single admission	9
2011	8	4.51E+00	1	5.84E-01	1	Single admission	9
2011	10	8.90E+00	0	9.33E-01	0	Single admission	9
2011	3	8.85E+00	1	9.07E+00	0	Repeated admissions	9
2011	11	4.49E+00	1	3.91E-01	1	Repeated admissions	9
2011	0	8.79E+00	0	2.00E-01	0	Single admission	9
2011	0	8.79E+00	1	1.23E+01	0	Single admission	9
2011	0	8.79E+00	0	4.05E-01	0	Single admission	9
2011	1	8.77E+00	1	1.34E-01	0	Single admission	9
2011	0	8.77E+00	1	7.51E-01	0	Single admission	9
2011	5	5.84E+00	0	7.51E-01	1	Repeated admissions	9
2011	0	8.76E+00	0	1.98E+00	0	Single admission	9
2011	18	8.75E+00	1	2.69E+00	0	Repeated admissions	9
2011	0	8.75E+00	0	3.36E-01	0	Single admission	9
2011	9	8.73E+00	0	1.43E+00	0	Repeated admissions	9
2011	11	5.65E+00	1	3.87E+00	1	Repeated admissions	9
2011	3	8.68E+00	0	3.52E+01	0	Repeated admissions	9
2011	16	8.68E+00	0	1.94E-01	0	Single admission	9
2011	0	8.67E+00	1	8.18E+00	0	Single admission	9
2011	0	8.66E+00	0	7.80E+00	0	Single admission	9
2011	1	8.64E+00	0	4.62E-01	0	Single admission	9

Admission year	Age (years)	Follow up time (years)	Sex (Females=1)	PDRLast	Outcome (Deceased=1)	Single/repeated admission	Diagnostic group
2011	8	8.64E+00	1	1.58E+00	0	Repeated admissions	9
2011	0	8.64E+00	1	2.18E-01	0	Single admission	9
2011	0	8.63E+00	0	2.56E+00	0	Repeated admissions	9
2011	1	8.63E+00	0	1.13E-01	0	Single admission	9
2011	13	8.60E+00	1	7.51E-01	0	Repeated admissions	9
2011	5	8.58E+00	0	1.34E-01	0	Single admission	9
2011	0	8.57E+00	0	2.68E-01	0	Single admission	9
2011	0	8.56E+00	1	2.03E+00	0	Single admission	9
2011	0	8.55E+00	1	2.13E+00	0	Single admission	9
2011	0	8.54E+00	0	1.79E+01	0	Repeated admissions	9
2011	1	8.53E+00	0	1.67E-01	0	Single admission	9
2011	0	8.50E+00	0	1.18E+01	0	Single admission	9
2011	5	9.47E+00	0	7.46E+00	0	Single admission	10
2011	11	9.44E+00	0	3.88E+00	0	Repeated admissions	10
2011	18	9.39E+00	0	7.51E-01	0	Repeated admissions	10
2011	3	9.38E+00	1	4.67E+00	0	Single admission	10
2011	1	9.33E+00	0	3.94E+01	0	Single admission	10
2011	7	9.32E+00	0	8.41E-01	0	Repeated admissions	10
2011	5	9.30E+00	0	5.19E+00	0	Single admission	10
2011	6	9.29E+00	0	7.51E-01	0	Single admission	10
2011	3	9.28E+00	0	9.83E-01	0	Single admission	10
2011	0	9.25E+00	0	7.52E+00	0	Single admission	10
2011	9	9.24E+00	1	3.17E-01	0	Repeated admissions	10
2011	8	9.23E+00	0	5.69E-01	0	Single admission	10
2011	9	9.23E+00	0	1.07E-01	0	Single admission	10
2011	1	2.48E-01	1	1.34E+01	1	Repeated admissions	10
2011	9	9.15E+00	0	1.52E+01	0	Single admission	10
2011	10	9.13E+00	1	9.11E-01	0	Single admission	10
2011	2	9.13E+00	0	6.37E+00	0	Single admission	10
2011	15	1.73E+00	0	4.47E+00	1	Single admission	10

Admission year	Age (years)	Follow up time (years)	Sex (Females=1)	PDRLast	Outcome (Deceased=1)	Single/repeated admission	Diagnostic group
2011	13	9.12E+00	0	7.83E-01	0	Single admission	10
2011	5	9.12E+00	1	9.24E-01	0	Repeated admissions	10
2011	5	9.11E+00	0	2.90E-01	0	Single admission	10
2011	1	9.11E+00	1	3.88E+00	0	Single admission	10
2011	10	9.10E+00	1	1.02E+00	0	Single admission	10
2011	6	9.07E+00	1	9.63E-01	0	Repeated admissions	10
2011	0	9.04E+00	0	3.88E+00	0	Single admission	10
2011	2	9.04E+00	0	4.41E-01	0	Single admission	10
2011	9	3.67E-01	1	7.51E-01	1	Repeated admissions	10
2011	3	9.01E+00	0	8.62E-01	0	Single admission	10
2011	13	1.68E+00	0	1.06E+00	1	Repeated admissions	10
2011	9	8.94E+00	1	7.51E-01	0	Repeated admissions	10
2011	14	8.92E+00	0	2.79E-01	0	Single admission	10
2011	12	8.85E+00	1	2.14E+00	0	Single admission	10
2011	2	8.85E+00	0	1.18E+00	0	Repeated admissions	10
2011	1	8.81E+00	1	9.79E-01	0	Single admission	10
2011	1	8.81E+00	0	9.90E-01	0	Repeated admissions	10
2011	6	8.81E+00	0	8.33E-01	0	Single admission	10
2011	6	4.63E+00	1	7.47E+01	1	Repeated admissions	10
2011	12	8.76E+00	1	7.51E-01	0	Single admission	10
2011	16	4.35E-01	1	3.91E+00	1	Repeated admissions	10
2011	3	8.71E+00	0	1.00E+00	0	Single admission	10
2011	1	8.70E+00	1	2.13E+01	0	Single admission	10
2011	2	8.67E+00	1	8.74E-01	0	Single admission	10
2011	0	8.65E+00	1	6.99E+00	0	Single admission	10
2011	8	8.65E+00	1	1.07E+00	0	Repeated admissions	10
2011	4	8.63E+00	1	4.96E+00	0	Single admission	10
2011	2	8.62E+00	1	4.75E+00	0	Single admission	10
2011	6	8.62E+00	0	2.85E+00	0	Single admission	10
2011	0	8.61E+00	1	3.88E+00	0	Repeated admissions	10

Admission year	Age (years)	Follow up time (years)	Sex (Females=1)	PDRLast	Outcome (Deceased=1)	Single/repeated admission	Diagnostic group
2011	11	8.57E+00	0	9.24E-01	0	Single admission	10
2011	4	2.24E+00	1	7.51E-01	1	Repeated admissions	10
2011	4	8.54E+00	0	7.51E-01	0	Single admission	10
2011	1	8.52E+00	1	7.51E-01	0	Single admission	10
2011	0	9.47E+00	0	8.57E-01	0	Single admission	11
2011	17	9.46E+00	0	1.29E+00	0	Single admission	11
2011	0	9.45E+00	1	1.41E+00	0	Repeated admissions	11
2011	0	9.44E+00	1	5.99E+00	0	Single admission	11
2011	3	9.43E+00	1	1.67E-01	0	Single admission	11
2011	1	9.43E+00	0	3.38E-01	0	Single admission	11
2011	0	9.43E+00	1	1.72E+00	0	Single admission	11
2011	1	9.42E+00	1	8.63E-01	0	Single admission	11
2011	1	9.42E+00	1	1.75E-01	0	Single admission	11
2011	0	9.42E+00	0	1.85E-01	0	Single admission	11
2011	0	9.42E+00	1	6.23E-01	0	Single admission	11
2011	7	4.88E+00	1	1.23E+00	1	Repeated admissions	11
2011	0	9.41E+00	0	3.42E-01	0	Repeated admissions	11
2011	0	9.39E+00	0	5.04E+00	0	Single admission	11
2011	0	9.37E+00	1	9.47E-01	0	Single admission	11
2011	4	9.33E+00	0	1.72E+00	0	Repeated admissions	11
2011	0	9.33E+00	1	7.74E-01	0	Single admission	11
2011	0	9.33E+00	0	1.30E+00	0	Single admission	11
2011	0	9.33E+00	0	9.85E-01	0	Single admission	11
2011	0	9.33E+00	0	1.50E+00	0	Single admission	11
2011	0	9.31E+00	0	5.22E+00	0	Single admission	11
2011	0	9.31E+00	0	2.57E+00	0	Repeated admissions	11
2011	0	9.30E+00	0	3.71E+01	0	Single admission	11
2011	0	9.26E+00	0	1.65E+00	0	Single admission	11
2011	0	9.24E+00	0	4.24E+00	0	Single admission	11
2011	5	9.23E+00	1	9.37E-01	0	Repeated admissions	11

Admission year	Age (years)	Follow up time (years)	Sex (Females=1)	PDRLast	Outcome (Deceased=1)	Single/repeated admission	Diagnostic group
2011	0	9.22E+00	1	7.89E+00	0	Single admission	11
2011	0	9.21E+00	0	5.56E-01	0	Repeated admissions	11
2011	1	9.19E+00	1	2.67E-01	0	Repeated admissions	11
2011	0	9.18E+00	0	4.52E-01	0	Single admission	11
2011	0	9.15E+00	0	8.41E+00	0	Repeated admissions	11
2011	1	9.14E+00	0	2.01E-01	0	Repeated admissions	11
2011	0	9.12E+00	0	3.88E+00	0	Single admission	11
2011	1	9.10E+00	0	1.81E+00	0	Repeated admissions	11
2011	0	9.08E+00	0	4.19E+00	0	Single admission	11
2011	0	7.39E+00	0	6.39E+00	0	Single admission	11
2011	0	9.04E+00	1	1.50E+00	0	Single admission	11
2011	0	9.04E+00	0	4.83E-01	0	Single admission	11
2011	1	9.04E+00	0	1.70E-01	0	Single admission	11
2011	0	9.01E+00	0	1.17E+00	0	Single admission	11
2011	0	9.01E+00	1	4.24E-01	0	Repeated admissions	11
2011	1	9.00E+00	0	2.71E-01	0	Repeated admissions	11
2011	0	8.93E+00	0	3.35E+00	0	Single admission	11
2011	0	8.91E+00	0	6.47E+00	0	Single admission	11
2011	0	8.90E+00	1	1.56E-01	0	Single admission	11
2011	0	8.88E+00	0	1.77E+00	0	Single admission	11
2011	0	2.74E-04	1	3.06E+01	1	Single admission	11
2011	0	8.86E+00	0	1.63E-01	0	Single admission	11
2011	0	8.82E+00	0	8.52E-01	0	Single admission	11
2011	4	7.19E+00	0	5.69E-01	1	Single admission	11
2011	0	8.81E+00	1	6.91E+00	0	Single admission	11
2011	0	8.81E+00	1	5.69E-01	0	Single admission	11
2011	0	8.80E+00	1	6.27E-01	0	Single admission	11
2011	0	1.30E+00	0	2.12E+00	1	Single admission	11
2011	1	8.78E+00	0	2.26E-01	0	Single admission	11
2011	0	3.61E-02	0	7.76E+00	1	Single admission	11

Admission year	Age (years)	Follow up time (years)	Sex (Females=1)	PDRLast	Outcome (Deceased=1)	Single/repeated admission	Diagnostic group
2011	2	8.75E+00	0	3.47E-01	0	Repeated admissions	11
2011	1	8.74E+00	1	5.23E-01	0	Repeated admissions	11
2011	2	1.79E+00	0	2.80E+00	1	Repeated admissions	11
2011	1	8.72E+00	1	4.32E-01	0	Single admission	11
2011	0	8.71E+00	0	3.05E+00	0	Single admission	11
2011	0	8.70E+00	0	6.93E+00	0	Single admission	11
2011	0	8.70E+00	1	3.22E+00	0	Single admission	11
2011	0	8.68E+00	0	9.66E+00	0	Single admission	11
2011	0	8.66E+00	1	1.26E+00	0	Repeated admissions	11
2011	1	8.66E+00	1	2.54E-01	0	Single admission	11
2011	0	8.62E+00	1	2.71E-01	0	Repeated admissions	11
2011	0	8.62E+00	1	1.34E+00	0	Single admission	11
2011	1	8.62E+00	1	2.18E-01	0	Single admission	11
2011	1	8.61E+00	0	4.41E-01	0	Single admission	11
2011	0	8.59E+00	0	9.51E+00	0	Single admission	11
2011	1	8.59E+00	1	1.74E-01	0	Repeated admissions	11
2011	0	8.56E+00	0	2.21E+00	0	Repeated admissions	11
2011	0	8.56E+00	1	2.44E-01	0	Single admission	11
2011	0	8.54E+00	0	8.37E+00	0	Single admission	11
2011	0	8.54E+00	0	3.88E+00	0	Repeated admissions	11
2011	0	8.52E+00	1	5.91E-01	0	Repeated admissions	11
2011	0	8.51E+00	1	1.33E+01	0	Single admission	11
2011	0	8.49E+00	1	1.33E+01	0	Single admission	11
2011	0	9.46E+00	0	1.56E-01	0	Single admission	12
2011	0	9.44E+00	0	1.42E+00	0	Single admission	12
2011	0	5.81E-02	0	3.54E-01	1	Single admission	12
2011	0	9.40E+00	1	7.51E-01	0	Single admission	12
2011	2	9.39E+00	0	7.99E+00	0	Single admission	12
2011	10	9.39E+00	0	6.34E+00	0	Repeated admissions	12
2011	9	9.37E+00	0	3.43E-01	0	Single admission	12

Admission year	Age (years)	Follow up time (years)	Sex (Females=1)	PDRLast	Outcome (Deceased=1)	Single/repeated admission	Diagnostic group
2011	2	9.37E+00	1	9.90E-01	0	Single admission	12
2011	0	9.36E+00	0	2.06E-01	0	Single admission	12
2011	3	9.36E+00	0	3.43E-01	0	Single admission	12
2011	0	9.35E+00	0	1.36E+00	0	Single admission	12
2011	1	8.60E+00	0	5.49E+00	0	Single admission	12
2011	0	9.34E+00	1	1.77E+00	0	Single admission	12
2011	0	9.34E+00	1	1.08E+00	0	Single admission	12
2011	2	9.34E+00	0	1.14E+00	0	Single admission	12
2011	0	9.34E+00	0	2.00E+00	0	Single admission	12
2011	0	9.34E+00	0	6.03E+00	0	Single admission	12
2011	0	9.33E+00	1	6.16E-01	0	Single admission	12
2011	0	9.33E+00	0	1.15E+01	0	Single admission	12
2011	0	9.32E+00	1	3.88E+00	0	Single admission	12
2011	0	9.32E+00	0	1.39E+00	0	Single admission	12
2011	0	9.31E+00	0	5.21E+00	0	Single admission	12
2011	0	9.31E+00	0	1.14E+00	0	Single admission	12
2011	6	9.31E+00	0	6.62E+00	0	Single admission	12
2011	0	9.31E+00	1	7.51E-01	0	Single admission	12
2011	1	9.30E+00	0	5.18E+01	0	Single admission	12
2011	0	9.29E+00	0	2.52E-01	0	Single admission	12
2011	0	9.29E+00	0	1.92E-01	0	Single admission	12
2011	0	9.29E+00	0	2.65E+01	0	Single admission	12
2011	0	9.28E+00	0	7.51E-01	0	Single admission	12
2011	3	9.28E+00	1	1.70E+00	0	Repeated admissions	12
2011	0	9.28E+00	0	1.50E+00	0	Single admission	12
2011	0	9.28E+00	0	3.49E+00	0	Single admission	12
2011	0	9.28E+00	0	4.86E-01	0	Repeated admissions	12
2011	6	9.28E+00	1	6.47E+00	0	Repeated admissions	12
2011	0	9.28E+00	0	2.80E+00	0	Single admission	12
2011	0	9.27E+00	1	1.05E+01	0	Single admission	12

Admission year	Age (years)	Follow up time (years)	Sex (Females=1)	PDRLast	Outcome (Deceased=1)	Single/repeated admission	Diagnostic group
2011	1	9.27E+00	0	2.63E-01	0	Single admission	12
2011	0	9.27E+00	0	9.90E+00	0	Single admission	12
2011	0	9.27E+00	0	2.25E+00	0	Single admission	12
2011	0	9.27E+00	1	2.37E+00	0	Single admission	12
2011	1	9.27E+00	0	1.23E+00	0	Single admission	12
2011	1	9.26E+00	1	4.04E+00	0	Single admission	12
2011	14	9.25E+00	1	4.78E+01	0	Single admission	12
2011	2	9.24E+00	0	2.11E+00	0	Repeated admissions	12
2011	0	9.24E+00	1	3.07E+00	0	Single admission	12
2011	0	9.24E+00	1	3.07E+00	0	Single admission	12
2011	0	9.23E+00	0	9.07E-01	0	Repeated admissions	12
2011	2	9.23E+00	0	4.73E+00	0	Repeated admissions	12
2011	0	9.22E+00	1	4.36E+00	0	Single admission	12
2011	0	9.22E+00	0	1.83E+00	0	Single admission	12
2011	0	9.20E+00	0	1.22E+00	0	Single admission	12
2011	17	9.20E+00	0	9.01E+00	0	Single admission	12
2011	0	9.17E+00	0	8.05E-01	0	Single admission	12
2011	0	9.16E+00	0	2.80E+00	0	Single admission	12
2011	1	9.12E+00	0	3.88E+00	0	Single admission	12
2011	0	9.12E+00	1	2.92E-01	0	Single admission	12
2011	11	9.11E+00	0	2.71E-01	0	Single admission	12
2011	11	7.23E-02	1	3.38E+00	1	Repeated admissions	12
2011	1	9.08E+00	0	4.41E+00	0	Single admission	12
2011	1	9.07E+00	0	1.19E+00	0	Single admission	12
2011	11	6.39E+00	0	4.66E+00	1	Repeated admissions	12
2011	1	9.05E+00	0	4.19E+00	0	Repeated admissions	12
2011	1	9.04E+00	0	8.62E-01	0	Single admission	12
2011	6	9.02E+00	1	5.97E+00	0	Repeated admissions	12
2011	0	9.02E+00	0	1.43E+00	0	Single admission	12
2011	1	9.01E+00	0	7.61E-01	0	Single admission	12

Admission year	Age (years)	Follow up time (years)	Sex (Females=1)	PDRLast	Outcome (Deceased=1)	Single/repeated admission	Diagnostic group
2011	2	8.98E+00	0	9.48E-01	0	Single admission	12
2011	0	8.92E+00	0	1.13E+01	0	Single admission	12
2011	2	8.87E+00	0	3.54E+01	0	Single admission	12
2011	11	8.83E+00	0	9.30E-01	0	Repeated admissions	12
2011	0	2.71E-01	1	1.15E+01	1	Single admission	12
2011	0	8.82E+00	1	1.02E+00	0	Single admission	12
2011	9	8.78E+00	1	9.56E-01	0	Single admission	12
2011	2	8.77E+00	0	1.54E+00	0	Single admission	12
2011	1	8.76E+00	1	1.62E+00	0	Repeated admissions	12
2011	4	8.76E+00	0	1.07E-01	0	Repeated admissions	12
2011	16	8.72E+00	1	7.51E-01	0	Single admission	12
2011	0	8.69E+00	1	1.33E+01	0	Single admission	12
2011	1	8.68E+00	0	3.88E+00	0	Single admission	12
2011	0	8.67E+00	0	7.83E+00	0	Single admission	12
2011	3	1.75E+00	0	2.28E+01	1	Single admission	12
2011	2	8.65E+00	0	5.76E+00	0	Single admission	12
2011	6	8.64E+00	1	7.61E-01	0	Single admission	12
2011	17	8.62E+00	0	4.97E+00	0	Repeated admissions	12
2011	0	8.62E+00	0	2.20E+01	0	Single admission	12
2011	1	3.37E+00	1	7.51E-01	0	Single admission	12
2011	16	8.60E+00	1	6.88E+00	0	Repeated admissions	12
2011	14	8.60E+00	0	7.72E-01	0	Single admission	12
2011	3	8.60E+00	0	5.51E-01	0	Single admission	12
2011	0	8.59E+00	0	7.66E+00	0	Single admission	12
2011	3	8.59E+00	0	2.32E+00	0	Single admission	12
2011	1	8.59E+00	0	1.71E+00	0	Single admission	12
2011	0	8.58E+00	0	6.19E+00	0	Single admission	12
2011	0	8.56E+00	1	1.94E+00	0	Single admission	12
2011	1	8.56E+00	1	1.73E+01	0	Single admission	12
2011	4	8.14E+00	0	2.98E-01	1	Repeated admissions	12

Admission year	Age (years)	Follow up time (years)	Sex (Females=1)	PDRLast	Outcome (Deceased=1)	Single/repeated admission	Diagnostic group
2011	15	1.86E-02	0	7.74E+00	1	Single admission	12
2011	1	8.56E+00	1	1.02E+00	0	Repeated admissions	12
2011	2	8.55E+00	0	4.29E+00	0	Single admission	12
2011	0	8.48E+00	0	1.72E+01	0	Single admission	12
2011	1	8.48E+00	1	7.51E-01	0	Single admission	12
2012	6	8.29E+00	1	2.44E+00	0	Single admission	1
2012	0	1.54E-01	1	1.07E+00	1	Single admission	1
2012	1	7.52E+00	1	5.72E-01	0	Repeated admissions	1
2012	0	8.44E+00	0	7.51E-01	0	Single admission	2
2012	0	6.09E-02	0	1.78E+01	1	Single admission	2
2012	0	8.35E+00	0	2.13E+00	0	Single admission	2
2012	0	8.23E+00	0	1.32E+00	0	Single admission	2
2012	0	2.17E-01	0	3.08E+00	1	Single admission	2
2012	4	8.03E+00	0	1.79E+00	0	Repeated admissions	2
2012	0	7.93E+00	1	4.20E+01	0	Single admission	2
2012	0	7.87E+00	1	4.48E-01	0	Single admission	2
2012	0	7.86E+00	0	7.51E-01	0	Single admission	2
2012	0	7.63E+00	0	2.80E+00	0	Repeated admissions	2
2012	0	7.59E+00	1	3.88E+00	0	Repeated admissions	2
2012	0	7.55E+00	0	7.36E-01	0	Single admission	2
2012	0	7.55E+00	1	9.92E-01	0	Repeated admissions	2
2012	0	7.54E+00	0	2.11E+00	0	Single admission	2
2012	14	7.51E+00	1	1.46E-01	0	Repeated admissions	2
2012	0	7.51E+00	1	1.55E+00	0	Single admission	2
2012	0	8.40E+00	1	1.76E+00	0	Single admission	3
2012	14	8.39E+00	0	8.78E-01	0	Single admission	3
2012	1	8.34E+00	0	4.68E+00	0	Single admission	3
2012	15	8.29E+00	0	9.37E-01	0	Repeated admissions	3
2012	11	8.25E+00	0	5.83E+00	0	Repeated admissions	3
2012	10	1.05E+00	0	5.60E+00	1	Repeated admissions	3

Admission year	Age (years)	Follow up time (years)	Sex (Females=1)	PDRLast	Outcome (Deceased=1)	Single/repeated admission	Diagnostic group
2012	1	8.16E+00	0	1.77E-01	0	Repeated admissions	3
2012	0	8.13E+00	1	3.16E-01	0	Repeated admissions	3
2012	3	8.09E+00	0	1.72E+00	0	Single admission	3
2012	0	8.03E+00	1	8.47E-01	0	Single admission	3
2012	0	7.99E+00	0	2.19E+00	0	Repeated admissions	3
2012	3	7.97E+00	1	1.97E+00	0	Repeated admissions	3
2012	8	7.95E+00	1	3.73E+01	0	Single admission	3
2012	11	7.90E+00	0	1.29E-01	0	Repeated admissions	3
2012	0	7.89E+00	1	6.48E+00	0	Single admission	3
2012	0	7.88E+00	0	2.23E-01	0	Single admission	3
2012	0	7.83E+00	0	6.23E-01	0	Single admission	3
2012	1	7.80E+00	1	7.51E-01	0	Single admission	3
2012	5	7.79E+00	1	1.42E-01	0	Single admission	3
2012	1	7.79E+00	0	7.51E-01	0	Single admission	3
2012	0	7.74E+00	0	1.17E+00	0	Single admission	3
2012	15	7.72E+00	0	1.23E-01	0	Single admission	3
2012	6	7.72E+00	1	1.88E+00	0	Single admission	3
2012	0	7.72E+00	0	2.47E+00	0	Single admission	3
2012	3	4.93E-01	1	7.36E+00	1	Single admission	3
2012	0	7.64E+00	0	2.71E-01	0	Single admission	3
2012	7	7.60E+00	0	9.43E-01	0	Single admission	3
2012	0	7.57E+00	0	2.11E-01	0	Repeated admissions	3
2012	0	7.48E+00	0	1.53E+00	0	Single admission	3
2012	10	8.47E+00	0	2.35E+01	0	Repeated admissions	4
2012	0	8.32E+00	0	1.63E+00	0	Single admission	4
2012	2	8.25E+00	0	2.18E+00	0	Single admission	4
2012	2	8.23E+00	1	2.03E+00	0	Single admission	4
2012	1	8.21E+00	0	4.89E+00	0	Single admission	4
2012	0	8.20E+00	0	7.51E-01	0	Single admission	4
2012	0	8.00E+00	1	5.64E+00	0	Repeated admissions	4

Admission year	Age (years)	Follow up time (years)	Sex (Females=1)	PDRLast	Outcome (Deceased=1)	Single/repeated admission	Diagnostic group
2012	0	7.94E+00	1	2.32E+00	0	Single admission	4
2012	0	7.94E+00	1	2.10E+00	0	Single admission	4
2012	14	7.92E+00	1	2.28E+00	0	Single admission	4
2012	0	7.88E+00	0	3.88E+00	0	Repeated admissions	4
2012	1	7.85E+00	1	8.62E-01	0	Single admission	4
2012	0	7.84E+00	0	1.30E+00	0	Single admission	4
2012	0	7.83E+00	0	7.51E-01	0	Single admission	4
2012	14	7.82E+00	0	1.20E+00	0	Single admission	4
2012	9	7.74E+00	0	1.33E+00	0	Repeated admissions	4
2012	0	7.74E+00	0	1.52E+00	0	Single admission	4
2012	0	7.72E+00	0	6.52E+01	0	Single admission	4
2012	16	7.69E+00	1	2.10E+00	0	Single admission	4
2012	3	7.69E+00	0	5.97E+00	0	Single admission	4
2012	14	7.62E+00	1	1.03E+00	0	Single admission	4
2012	3	7.61E+00	0	1.54E+00	0	Single admission	4
2012	0	7.57E+00	1	1.11E+00	0	Repeated admissions	4
2012	0	7.56E+00	1	1.34E+00	0	Single admission	4
2012	1	7.50E+00	1	3.47E-01	0	Single admission	4
2012	3	1.43E+00	1	1.85E+01	0	Single admission	5
2012	0	8.38E+00	0	1.32E-01	0	Single admission	5
2012	5	8.37E+00	0	1.51E+00	0	Repeated admissions	5
2012	1	8.31E+00	0	1.33E+01	0	Single admission	5
2012	2	8.31E+00	1	6.21E+00	0	Single admission	5
2012	0	2.63E+00	0	1.23E+00	1	Repeated admissions	5
2012	13	8.11E+00	1	1.72E-01	0	Single admission	5
2012	7	1.33E+00	1	1.72E-01	1	Single admission	5
2012	3	7.91E+00	0	5.60E-01	0	Single admission	5
2012	5	7.47E-01	1	1.67E+00	1	Single admission	5
2012	13	6.90E+00	1	2.79E-01	1	Single admission	5
2012	12	7.69E+00	1	4.78E-01	0	Single admission	5

Admission year	Age (years)	Follow up time (years)	Sex (Females=1)	PDRLast	Outcome (Deceased=1)	Single/repeated admission	Diagnostic group
2012	3	2.80E+00	0	1.54E+00	1	Single admission	5
2012	3	7.62E+00	0	7.74E-01	0	Single admission	5
2012	8	2.46E+00	1	3.13E-01	1	Single admission	5
2012	1	7.61E+00	1	1.12E+00	0	Single admission	5
2012	14	7.58E+00	1	1.40E-01	0	Single admission	5
2012	3	8.03E-01	0	1.20E+00	1	Repeated admissions	5
2012	7	7.57E+00	1	5.27E-01	0	Repeated admissions	5
2012	13	5.94E+00	0	2.22E-01	1	Single admission	5
2012	13	7.54E+00	0	2.62E+00	0	Single admission	5
2012	6	7.53E+00	1	7.52E-01	0	Single admission	5
2012	0	8.26E+00	1	1.45E+01	0	Single admission	6
2012	0	8.23E+00	0	7.00E+00	0	Single admission	6
2012	0	8.21E+00	0	4.38E+01	0	Repeated admissions	6
2012	13	1.46E+00	1	1.97E+00	1	Repeated admissions	6
2012	0	8.11E+00	1	1.40E+00	0	Single admission	6
2012	0	6.82E-03	1	9.38E+01	1	Single admission	6
2012	2	8.05E+00	1	1.07E-01	0	Repeated admissions	6
2012	0	1.98E-02	0	1.06E+00	1	Single admission	6
2012	0	8.00E+00	0	4.95E+00	0	Repeated admissions	6
2012	15	1.48E-02	0	5.56E+01	1	Single admission	6
2012	2	7.94E+00	0	4.60E-01	0	Single admission	6
2012	0	7.91E+00	1	1.49E+01	0	Single admission	6
2012	0	7.86E+00	0	7.14E+01	0	Single admission	6
2012	6	7.86E+00	0	6.08E+00	0	Single admission	6
2012	0	7.84E+00	1	2.01E+01	0	Single admission	6
2012	13	7.78E+00	1	1.55E+00	0	Single admission	6
2012	15	7.75E+00	0	1.45E+00	0	Single admission	6
2012	14	7.74E+00	1	1.29E-01	0	Repeated admissions	6
2012	10	7.70E+00	0	7.64E+00	0	Repeated admissions	6
2012	0	7.68E+00	1	6.16E-01	0	Single admission	6

Admission year	Age (years)	Follow up time (years)	Sex (Females=1)	PDRLast	Outcome (Deceased=1)	Single/repeated admission	Diagnostic group
2012	8	7.62E+00	1	1.30E+00	0	Single admission	6
2012	0	1.25E-01	0	1.30E+00	1	Repeated admissions	6
2012	1	7.52E+00	1	1.02E+00	0	Single admission	6
2012	13	8.45E+00	0	7.38E-01	0	Single admission	7
2012	2	8.36E+00	0	9.38E+00	0	Single admission	7
2012	18	8.26E+00	0	2.51E+00	0	Single admission	7
2012	4	4.72E-01	0	1.71E+01	1	Repeated admissions	7
2012	2	8.24E+00	0	1.35E+01	0	Single admission	7
2012	0	8.22E+00	0	1.78E+00	0	Single admission	7
2012	16	8.18E+00	0	1.54E+00	0	Single admission	7
2012	0	7.50E-01	0	7.86E+00	0	Single admission	7
2012	10	8.10E+00	1	8.49E+00	0	Single admission	7
2012	2	8.09E+00	1	1.63E+00	0	Single admission	7
2012	17	8.09E+00	1	3.10E+00	0	Single admission	7
2012	1	8.08E+00	0	8.70E-01	0	Single admission	7
2012	11	8.07E+00	1	4.80E+00	0	Single admission	7
2012	12	8.02E+00	0	4.95E+00	0	Single admission	7
2012	8	8.01E+00	0	2.75E+00	0	Single admission	7
2012	5	7.99E+00	1	7.65E-01	0	Single admission	7
2012	1	6.50E-01	1	5.71E+00	1	Repeated admissions	7
2012	3	7.91E+00	0	1.80E-01	0	Repeated admissions	7
2012	1	7.91E+00	1	1.56E+01	0	Single admission	7
2012	0	7.91E+00	0	1.02E+01	0	Single admission	7
2012	4	3.67E-02	0	6.99E-01	1	Single admission	7
2012	13	7.73E+00	0	1.09E+01	0	Single admission	7
2012	3	7.71E+00	0	1.00E+00	0	Single admission	7
2012	1	7.71E+00	1	9.07E+00	0	Single admission	7
2012	15	7.70E+00	0	1.07E+00	0	Single admission	7
2012	5	7.69E+00	1	8.65E-01	0	Single admission	7
2012	15	7.64E+00	1	1.45E+00	0	Repeated admissions	7

Admission year	Age (years)	Follow up time (years)	Sex (Females=1)	PDRLast	Outcome (Deceased=1)	Single/repeated admission	Diagnostic group
2012	14	7.63E+00	0	7.05E+00	0	Repeated admissions	7
2012	2	7.60E+00	0	7.30E+00	0	Repeated admissions	7
2012	3	7.59E+00	0	5.52E+00	0	Single admission	7
2012	5	7.58E+00	0	2.64E-01	0	Single admission	7
2012	18	7.54E+00	0	4.36E+00	0	Single admission	7
2012	14	7.52E+00	1	3.75E+00	0	Single admission	7
2012	10	7.52E+00	1	1.73E+00	0	Single admission	7
2012	9	7.49E+00	0	1.34E+01	0	Single admission	7
2012	3	7.49E+00	1	1.53E+00	0	Single admission	7
2012	1	8.44E+00	1	6.23E-01	0	Single admission	8
2012	2	8.44E+00	0	4.23E-01	0	Single admission	8
2012	6	8.42E+00	0	9.90E-01	0	Single admission	8
2012	1	8.41E+00	0	3.88E+00	0	Single admission	8
2012	1	8.40E+00	1	2.90E-01	0	Repeated admissions	8
2012	2	8.40E+00	0	1.95E+00	0	Single admission	8
2012	0	8.39E+00	0	3.88E+00	0	Single admission	8
2012	5	8.39E+00	0	2.80E+00	0	Single admission	8
2012	15	8.31E+00	0	2.80E+00	0	Single admission	8
2012	9	4.23E+00	0	2.73E+01	1	Single admission	8
2012	0	8.29E+00	0	6.35E-01	0	Single admission	8
2012	15	8.22E+00	1	3.88E+00	0	Single admission	8
2012	14	8.21E+00	0	8.27E-01	0	Single admission	8
2012	1	8.21E+00	0	1.25E+00	0	Single admission	8
2012	11	8.16E+00	0	2.99E+00	0	Single admission	8
2012	14	8.15E+00	1	7.51E-01	0	Single admission	8
2012	11	8.14E+00	0	4.53E+00	0	Single admission	8
2012	3	8.10E+00	1	1.06E+00	0	Single admission	8
2012	15	8.08E+00	0	6.06E+00	0	Single admission	8
2012	5	8.08E+00	0	9.24E-01	0	Single admission	8
2012	6	8.07E+00	1	9.90E-01	0	Single admission	8

Admission year	Age (years)	Follow up time (years)	Sex (Females=1)	PDRLast	Outcome (Deceased=1)	Single/repeated admission	Diagnostic group
2012	2	8.01E+00	0	1.23E+00	0	Single admission	8
2012	13	8.01E+00	1	7.59E-01	0	Single admission	8
2012	7	7.97E+00	0	7.91E-01	0	Single admission	8
2012	1	7.97E+00	0	1.43E+00	0	Single admission	8
2012	8	7.97E+00	1	9.24E-01	0	Single admission	8
2012	2	7.96E+00	1	1.40E+00	0	Single admission	8
2012	2	7.96E+00	0	1.06E+00	0	Single admission	8
2012	2	7.96E+00	1	1.93E+00	0	Single admission	8
2012	2	7.90E+00	1	4.59E+00	0	Single admission	8
2012	0	6.91E-03	0	9.80E+01	1	Single admission	8
2012	5	7.88E+00	0	8.62E-01	0	Single admission	8
2012	16	7.88E+00	0	9.50E-01	0	Single admission	8
2012	16	7.83E+00	1	2.20E-01	0	Repeated admissions	8
2012	6	7.79E+00	0	1.15E+00	0	Single admission	8
2012	13	7.78E+00	1	7.51E-01	0	Repeated admissions	8
2012	6	7.78E+00	0	9.63E-01	0	Single admission	8
2012	1	3.42E+00	0	1.25E+00	0	Single admission	8
2012	6	7.74E+00	1	1.17E+00	0	Single admission	8
2012	9	7.69E+00	0	1.15E+00	0	Single admission	8
2012	7	7.69E+00	0	7.22E+00	0	Single admission	8
2012	12	7.67E+00	1	1.05E+00	0	Single admission	8
2012	8	7.66E+00	0	7.51E-01	0	Single admission	8
2012	5	7.65E+00	0	9.90E-01	0	Single admission	8
2012	1	7.65E+00	1	6.66E+00	0	Single admission	8
2012	13	7.63E+00	1	9.09E-01	0	Single admission	8
2012	17	7.63E+00	0	1.37E+00	0	Single admission	8
2012	11	7.59E+00	0	3.00E+00	0	Single admission	8
2012	12	7.55E+00	0	8.74E-01	0	Single admission	8
2012	2	7.48E+00	0	1.32E+00	0	Single admission	8
2012	1	2.33E+00	0	5.74E-01	1	Single admission	9

Admission year	Age (years)	Follow up time (years)	Sex (Females=1)	PDRLast	Outcome (Deceased=1)	Single/repeated admission	Diagnostic group
2012	1	8.44E+00	0	1.07E-01	0	Single admission	9
2012	0	8.39E+00	1	1.88E+00	0	Single admission	9
2012	0	6.42E+00	0	7.51E-01	1	Single admission	9
2012	9	8.39E+00	0	7.83E-01	0	Single admission	9
2012	2	8.38E+00	0	1.73E-01	0	Repeated admissions	9
2012	15	8.37E+00	1	1.63E-01	0	Single admission	9
2012	5	8.37E+00	0	1.08E+00	0	Single admission	9
2012	0	8.36E+00	0	2.70E+00	0	Single admission	9
2012	8	8.35E+00	0	1.61E-01	0	Single admission	9
2012	6	2.79E+00	0	3.52E+00	1	Repeated admissions	9
2012	8	8.32E+00	0	4.46E-01	0	Single admission	9
2012	11	8.29E+00	1	2.68E-01	0	Single admission	9
2012	0	8.28E+00	0	6.08E-01	0	Single admission	9
2012	8	8.27E+00	1	3.34E-01	0	Single admission	9
2012	1	8.27E+00	0	1.87E-01	0	Single admission	9
2012	10	8.25E+00	0	1.74E-01	0	Single admission	9
2012	0	8.24E+00	0	1.72E-01	0	Single admission	9
2012	14	8.20E+00	1	1.26E+01	0	Single admission	9
2012	13	8.14E+00	1	1.07E-01	0	Single admission	9
2012	15	8.12E+00	0	1.43E+00	0	Single admission	9
2012	11	8.09E+00	1	1.69E-01	0	Single admission	9
2012	0	8.07E+00	0	1.43E+00	0	Single admission	9
2012	1	8.07E+00	0	2.19E-01	0	Single admission	9
2012	0	8.07E+00	0	2.12E+01	0	Repeated admissions	9
2012	2	8.06E+00	0	5.25E-01	0	Single admission	9
2012	2	8.05E+00	0	3.83E-01	0	Single admission	9
2012	4	7.56E+00	0	5.74E-01	1	Single admission	9
2012	2	8.03E+00	1	7.38E-01	0	Repeated admissions	9
2012	1	9.16E-01	0	4.55E+01	1	Repeated admissions	9
2012	11	7.97E+00	1	2.08E-01	0	Single admission	9

Admission year	Age (years)	Follow up time (years)	Sex (Females=1)	PDRLast	Outcome (Deceased=1)	Single/repeated admission	Diagnostic group
2012	1	6.99E-01	1	1.97E+01	1	Repeated admissions	9
2012	3	7.95E+00	1	1.09E-01	0	Single admission	9
2012	13	7.93E+00	1	1.51E-01	0	Single admission	9
2012	0	7.92E+00	0	2.71E-01	0	Single admission	9
2012	9	7.91E+00	1	1.25E-01	0	Single admission	9
2012	9	7.89E+00	0	5.01E-01	0	Single admission	9
2012	3	7.84E+00	0	2.71E-01	0	Single admission	9
2012	0	7.84E+00	0	5.90E-01	0	Single admission	9
2012	10	7.83E+00	1	6.75E-01	0	Single admission	9
2012	0	7.82E+00	0	5.72E-01	0	Single admission	9
2012	5	7.81E+00	0	1.82E+00	0	Repeated admissions	9
2012	11	7.80E+00	0	5.74E-01	0	Single admission	9
2012	14	7.80E+00	1	2.03E-01	0	Single admission	9
2012	12	7.76E+00	1	2.08E-01	0	Single admission	9
2012	0	7.75E+00	0	1.07E-01	0	Single admission	9
2012	7	7.74E+00	0	1.07E-01	0	Single admission	9
2012	15	7.72E+00	1	4.67E+00	0	Repeated admissions	9
2012	0	7.72E+00	1	5.28E+00	0	Single admission	9
2012	0	8.82E-02	0	2.87E+01	1	Repeated admissions	9
2012	9	7.68E+00	1	1.68E-01	0	Single admission	9
2012	14	7.67E+00	1	1.32E-01	0	Single admission	9
2012	13	7.67E+00	1	1.11E+00	0	Single admission	9
2012	8	7.66E+00	1	1.70E+00	0	Single admission	9
2012	0	7.65E+00	0	1.90E-01	0	Single admission	9
2012	0	7.64E+00	0	4.72E-01	0	Single admission	9
2012	13	7.64E+00	1	4.80E-01	0	Single admission	9
2012	12	7.62E+00	0	5.89E-01	0	Repeated admissions	9
2012	0	7.61E+00	1	1.82E-01	0	Single admission	9
2012	7	7.60E+00	0	2.94E-01	0	Single admission	9
2012	1	7.60E+00	0	5.22E-01	0	Single admission	9

Admission year	Age (years)	Follow up time (years)	Sex (Females=1)	PDRLast	Outcome (Deceased=1)	Single/repeated admission	Diagnostic group
2012	10	7.55E+00	0	1.05E+00	0	Single admission	9
2012	1	7.53E+00	1	6.61E-01	0	Repeated admissions	9
2012	12	7.53E+00	1	2.82E-01	0	Single admission	9
2012	0	7.52E+00	1	1.93E-01	0	Single admission	9
2012	0	7.52E+00	1	1.07E-01	0	Single admission	9
2012	13	7.52E+00	0	1.43E+00	0	Single admission	9
2012	6	7.51E+00	0	5.22E+00	0	Single admission	9
2012	14	7.49E+00	0	3.48E-01	0	Single admission	9
2012	9	7.49E+00	0	2.71E-01	0	Single admission	9
2012	4	7.48E+00	0	7.51E-01	0	Single admission	9
2012	1	8.47E+00	1	1.43E+00	0	Single admission	10
2012	3	8.46E+00	1	1.14E+00	0	Single admission	10
2012	16	8.45E+00	1	5.43E+01	0	Single admission	10
2012	4	8.44E+00	0	3.39E+00	0	Single admission	10
2012	1	8.41E+00	0	9.24E-01	0	Single admission	10
2012	0	8.39E+00	1	1.16E+01	0	Repeated admissions	10
2012	0	8.38E+00	1	7.51E-01	0	Single admission	10
2012	3	8.37E+00	0	3.88E+00	0	Single admission	10
2012	2	8.34E+00	0	3.88E+00	0	Single admission	10
2012	8	1.76E+00	0	7.75E+00	1	Repeated admissions	10
2012	0	8.33E+00	1	6.95E+00	0	Single admission	10
2012	8	8.32E+00	1	1.33E+00	0	Single admission	10
2012	1	8.30E+00	1	6.40E+00	0	Single admission	10
2012	7	8.27E+00	1	8.27E-01	0	Repeated admissions	10
2012	1	8.27E+00	0	4.33E+00	0	Single admission	10
2012	9	8.26E+00	0	1.35E+01	0	Single admission	10
2012	2	8.23E+00	1	2.80E+00	0	Single admission	10
2012	15	8.18E+00	0	9.82E-01	0	Repeated admissions	10
2012	5	8.17E+00	1	1.33E+00	0	Single admission	10
2012	9	8.16E+00	1	1.96E+00	0	Repeated admissions	10

Admission year	Age (years)	Follow up time (years)	Sex (Females=1)	PDRLast	Outcome (Deceased=1)	Single/repeated admission	Diagnostic group
2012	1	4.86E-01	1	1.21E+00	1	Single admission	10
2012	17	8.12E+00	0	7.51E-01	0	Repeated admissions	10
2012	1	8.10E+00	0	2.41E+00	0	Single admission	10
2012	0	4.68E-01	1	1.14E+00	1	Single admission	10
2012	5	8.06E+00	1	1.05E+00	0	Single admission	10
2012	6	8.05E+00	0	8.99E-01	0	Repeated admissions	10
2012	0	8.03E+00	0	5.91E-01	0	Repeated admissions	10
2012	3	8.00E+00	1	1.77E+00	0	Single admission	10
2012	11	7.96E+00	1	4.72E-01	0	Repeated admissions	10
2012	4	3.67E+00	0	1.43E+00	1	Repeated admissions	10
2012	2	7.87E+00	0	2.40E-02	0	Single admission	10
2012	0	6.93E-02	1	6.47E+00	1	Repeated admissions	10
2012	0	7.77E+00	0	1.74E+00	0	Single admission	10
2012	1	7.77E+00	1	2.15E-01	0	Repeated admissions	10
2012	2	7.75E+00	1	4.02E+00	0	Single admission	10
2012	17	7.66E+00	0	1.10E+00	0	Single admission	10
2012	9	7.62E+00	0	8.99E-01	0	Single admission	10
2012	6	7.62E+00	1	1.18E+00	0	Single admission	10
2012	14	7.58E+00	1	1.65E-01	0	Repeated admissions	10
2012	3	7.57E+00	0	7.74E+00	0	Single admission	10
2012	1	3.62E-01	1	1.32E+00	1	Single admission	10
2012	11	7.52E+00	0	2.71E-01	0	Single admission	10
2012	11	2.16E-02	0	6.64E+00	1	Single admission	10
2012	11	7.51E+00	0	8.16E-01	0	Single admission	10
2012	6	7.50E+00	0	9.97E-01	0	Single admission	10
2012	3	7.50E+00	1	7.63E+00	0	Single admission	10
2012	6	7.49E+00	0	8.05E-01	0	Single admission	10
2012	0	8.45E+00	0	1.22E+00	0	Single admission	11
2012	0	8.40E+00	0	5.69E-01	0	Repeated admissions	11
2012	5	8.39E+00	1	2.02E-01	0	Single admission	11

Admission year	Age (years)	Follow up time (years)	Sex (Females=1)	PDRLast	Outcome (Deceased=1)	Single/repeated admission	Diagnostic group
2012	0	8.35E+00	1	3.15E+00	0	Single admission	11
2012	0	8.34E+00	1	1.71E+00	0	Repeated admissions	11
2012	0	8.33E+00	1	2.76E+00	0	Single admission	11
2012	2	8.33E+00	0	9.34E-01	0	Repeated admissions	11
2012	0	8.31E+00	0	5.22E+00	0	Single admission	11
2012	0	8.30E+00	0	1.25E+00	0	Single admission	11
2012	1	8.24E+00	0	1.07E-01	0	Single admission	11
2012	0	8.22E+00	0	4.97E+00	0	Single admission	11
2012	0	8.20E+00	1	2.07E+01	0	Single admission	11
2012	12	8.19E+00	1	2.83E+00	0	Single admission	11
2012	0	6.72E-02	1	1.50E+01	1	Single admission	11
2012	0	8.16E+00	0	1.10E+01	0	Single admission	11
2012	0	2.17E-02	0	1.08E+01	1	Single admission	11
2012	0	8.14E+00	0	5.83E+00	0	Single admission	11
2012	0	8.13E+00	0	2.71E-01	0	Repeated admissions	11
2012	0	6.34E+00	1	3.88E+00	0	Repeated admissions	11
2012	1	8.12E+00	0	1.70E-01	0	Single admission	11
2012	0	8.12E+00	0	3.55E+00	0	Single admission	11
2012	0	8.07E+00	0	1.43E+00	0	Single admission	11
2012	0	8.07E+00	1	9.45E-01	0	Single admission	11
2012	0	8.06E+00	0	9.77E-01	0	Single admission	11
2012	0	8.06E+00	1	7.51E-01	0	Single admission	11
2012	0	8.05E+00	1	6.30E-01	0	Repeated admissions	11
2012	0	8.03E+00	1	6.30E+00	0	Single admission	11
2012	0	8.01E+00	0	1.22E+00	0	Single admission	11
2012	0	8.01E+00	1	3.20E-01	0	Single admission	11
2012	0	8.00E+00	0	2.34E+00	0	Single admission	11
2012	1	7.94E+00	1	9.02E-01	0	Single admission	11
2012	0	4.53E-01	1	5.18E+00	1	Single admission	11
2012	0	1.15E+00	0	2.71E-01	1	Repeated admissions	11

Admission year	Age (years)	Follow up time (years)	Sex (Females=1)	PDRLast	Outcome (Deceased=1)	Single/repeated admission	Diagnostic group
2012	3	7.92E+00	1	7.59E+00	0	Repeated admissions	11
2012	0	7.88E+00	0	2.66E+01	0	Single admission	11
2012	0	7.88E+00	1	2.57E+01	0	Single admission	11
2012	0	7.84E+00	0	1.36E+00	0	Single admission	11
2012	0	7.80E+00	1	4.47E-01	0	Single admission	11
2012	0	7.79E+00	0	8.39E-01	0	Single admission	11
2012	0	7.78E+00	0	4.02E-01	0	Repeated admissions	11
2012	10	7.75E+00	1	1.49E-01	0	Single admission	11
2012	0	7.74E+00	0	3.85E+00	0	Single admission	11
2012	0	7.74E+00	0	4.55E-01	0	Repeated admissions	11
2012	0	7.71E+00	1	2.62E+01	0	Single admission	11
2012	0	1.40E-01	1	5.58E+00	1	Repeated admissions	11
2012	0	7.68E+00	0	2.98E-01	0	Single admission	11
2012	0	7.66E+00	1	7.52E+01	0	Single admission	11
2012	0	3.46E-01	0	3.61E+00	1	Repeated admissions	11
2012	0	7.60E+00	1	2.38E-01	0	Single admission	11
2012	3	7.58E+00	0	8.27E-01	0	Repeated admissions	11
2012	0	7.57E+00	0	2.98E+00	0	Single admission	11
2012	3	7.57E+00	1	7.51E-01	0	Single admission	11
2012	0	7.57E+00	0	7.51E-01	0	Repeated admissions	11
2012	17	7.56E+00	1	2.12E+00	0	Repeated admissions	11
2012	1	6.17E-01	0	3.91E+00	1	Repeated admissions	11
2012	0	7.54E+00	0	1.46E+00	0	Single admission	11
2012	0	7.51E+00	0	9.26E-01	0	Repeated admissions	11
2012	2	8.47E+00	0	7.83E-01	0	Single admission	12
2012	4	8.47E+00	1	1.53E+00	0	Single admission	12
2012	1	8.47E+00	1	9.23E-01	0	Single admission	12
2012	3	8.47E+00	0	8.07E+00	0	Single admission	12
2012	0	8.47E+00	1	3.43E+00	0	Single admission	12
2012	0	8.46E+00	1	3.07E+00	0	Single admission	12

Admission year	Age (years)	Follow up time (years)	Sex (Females=1)	PDRLast	Outcome (Deceased=1)	Single/repeated admission	Diagnostic group
2012	3	8.45E+00	0	7.36E+00	0	Single admission	12
2012	1	8.44E+00	1	1.65E-01	0	Single admission	12
2012	1	1.25E+00	1	1.27E+00	1	Repeated admissions	12
2012	0	8.41E+00	1	1.48E+01	0	Single admission	12
2012	3	8.40E+00	1	1.42E+00	0	Single admission	12
2012	0	8.39E+00	0	1.02E+00	0	Single admission	12
2012	2	8.39E+00	1	3.88E+00	0	Single admission	12
2012	2	8.39E+00	1	8.27E-01	0	Single admission	12
2012	0	8.38E+00	1	3.88E+00	0	Single admission	12
2012	12	8.38E+00	1	8.85E-01	0	Single admission	12
2012	2	8.38E+00	0	8.49E+00	0	Single admission	12
2012	2	8.38E+00	0	1.76E+00	0	Single admission	12
2012	3	8.38E+00	1	1.67E-01	0	Single admission	12
2012	0	8.38E+00	0	2.94E-01	0	Single admission	12
2012	2	8.37E+00	0	1.14E+00	0	Single admission	12
2012	3	8.37E+00	0	8.19E+00	0	Single admission	12
2012	1	8.37E+00	0	3.83E-01	0	Single admission	12
2012	0	8.37E+00	1	1.02E+00	0	Single admission	12
2012	1	8.36E+00	1	1.03E+00	0	Single admission	12
2012	0	8.35E+00	0	5.91E+00	0	Single admission	12
2012	0	8.35E+00	0	3.88E+00	0	Single admission	12
2012	1	6.85E-03	1	3.02E+00	1	Single admission	12
2012	0	3.13E-01	1	9.88E+01	1	Repeated admissions	12
2012	0	8.35E+00	1	3.88E+00	0	Single admission	12
2012	14	5.89E+00	1	2.33E+01	1	Repeated admissions	12
2012	0	8.34E+00	1	1.12E+00	0	Single admission	12
2012	5	8.34E+00	1	1.56E+00	0	Single admission	12
2012	0	8.32E+00	0	2.57E+01	0	Single admission	12
2012	17	8.32E+00	1	5.69E-01	0	Single admission	12
2012	0	8.31E+00	0	2.19E+00	0	Single admission	12

Admission year	Age (years)	Follow up time (years)	Sex (Females=1)	PDRLast	Outcome (Deceased=1)	Single/repeated admission	Diagnostic group
2012	0	1.16E-01	0	2.57E+01	1	Single admission	12
2012	4	8.31E+00	1	1.87E+00	0	Single admission	12
2012	17	1.22E-01	1	3.99E+00	1	Repeated admissions	12
2012	0	8.29E+00	1	3.19E+00	0	Single admission	12
2012	0	8.29E+00	1	1.28E+00	0	Single admission	12
2012	1	8.27E+00	1	7.83E-01	0	Single admission	12
2012	2	8.27E+00	1	8.62E-01	0	Single admission	12
2012	0	8.27E+00	1	3.07E+00	0	Repeated admissions	12
2012	0	8.27E+00	1	1.54E+00	0	Repeated admissions	12
2012	1	8.27E+00	0	4.30E+00	0	Single admission	12
2012	0	8.25E+00	1	1.11E+00	0	Single admission	12
2012	2	8.24E+00	1	8.88E-01	0	Single admission	12
2012	0	8.23E+00	1	2.29E+00	0	Single admission	12
2012	0	8.23E+00	0	1.40E+00	0	Single admission	12
2012	4	8.22E+00	1	5.49E+00	0	Single admission	12
2012	0	8.21E+00	0	7.71E+00	0	Single admission	12
2012	14	8.21E+00	1	1.03E+00	0	Single admission	12
2012	6	8.21E+00	0	3.68E-01	0	Repeated admissions	12
2012	4	1.40E+00	0	1.85E+00	1	Repeated admissions	12
2012	0	8.21E+00	1	3.88E+00	0	Single admission	12
2012	0	8.20E+00	1	1.14E+00	0	Single admission	12
2012	0	8.20E+00	1	1.21E+00	0	Single admission	12
2012	0	8.20E+00	0	2.11E-01	0	Single admission	12
2012	0	8.20E+00	0	1.34E+00	0	Single admission	12
2012	3	8.19E+00	1	1.40E+00	0	Repeated admissions	12
2012	1	8.18E+00	0	1.91E+01	0	Single admission	12
2012	0	8.18E+00	1	4.41E+00	0	Single admission	12
2012	17	8.18E+00	0	4.66E-01	0	Single admission	12
2012	0	8.18E+00	1	3.88E+00	0	Single admission	12
2012	3	8.17E+00	0	7.00E+00	0	Single admission	12

Admission year	Age (years)	Follow up time (years)	Sex (Females=1)	PDRLast	Outcome (Deceased=1)	Single/repeated admission	Diagnostic group
2012	10	8.16E+00	0	1.02E+00	0	Repeated admissions	12
2012	1	8.14E+00	0	3.91E+00	0	Repeated admissions	12
2012	2	8.11E+00	0	2.34E+00	0	Repeated admissions	12
2012	0	5.80E+00	0	1.52E+00	0	Single admission	12
2012	4	7.21E+00	0	9.10E+00	1	Single admission	12
2012	1	8.10E+00	0	3.28E+00	0	Single admission	12
2012	1	2.16E-01	1	3.22E+00	1	Repeated admissions	12
2012	2	1.60E+00	1	1.06E+00	0	Single admission	12
2012	2	8.06E+00	1	7.37E+00	0	Single admission	12
2012	0	8.02E+00	0	1.48E+00	0	Single admission	12
2012	8	8.02E+00	0	6.39E+00	0	Single admission	12
2012	1	5.04E-02	1	8.27E+01	1	Single admission	12
2012	3	7.98E+00	1	1.11E+01	0	Single admission	12
2012	3	7.97E+00	0	8.30E+00	0	Repeated admissions	12
2012	11	7.91E+00	0	3.56E+00	0	Single admission	12
2012	0	6.14E-02	0	3.27E+00	1	Single admission	12
2012	0	7.88E+00	1	2.80E+00	0	Single admission	12
2012	0	2.85E-01	0	2.84E+00	1	Repeated admissions	12
2012	0	7.83E+00	1	8.91E-01	0	Repeated admissions	12
2012	0	7.83E+00	0	2.84E+00	0	Single admission	12
2012	6	7.81E+00	0	2.05E-01	0	Repeated admissions	12
2012	1	7.78E+00	0	1.16E+00	0	Single admission	12
2012	4	7.76E+00	0	9.40E-01	0	Single admission	12
2012	5	7.76E+00	1	1.23E+00	0	Single admission	12
2012	1	7.72E+00	1	5.14E+00	0	Single admission	12
2012	1	7.70E+00	0	3.42E-01	0	Repeated admissions	12
2012	1	7.69E+00	1	4.16E+00	0	Single admission	12
2012	0	7.63E+00	1	6.32E+00	0	Single admission	12
2012	1	7.63E+00	0	2.23E+00	0	Single admission	12
2012	0	7.61E+00	1	2.46E+00	0	Single admission	12

Admission year	Age (years)	Follow up time (years)	Sex (Females=1)	PDRLast	Outcome (Deceased=1)	Single/repeated admission	Diagnostic group
2012	0	7.60E+00	1	1.13E+00	0	Single admission	12
2012	18	7.59E+00	0	1.14E+00	0	Single admission	12
2012	0	2.88E+00	1	9.35E+00	0	Single admission	12
2012	2	7.58E+00	1	4.11E-01	0	Repeated admissions	12
2012	2	7.58E+00	0	1.07E+00	0	Repeated admissions	12
2012	10	7.57E+00	0	2.03E+00	0	Single admission	12
2012	0	7.57E+00	0	1.63E-01	0	Single admission	12
2012	1	7.56E+00	0	7.51E-01	0	Single admission	12
2012	1	7.56E+00	0	2.14E-01	0	Repeated admissions	12
2012	0	7.53E+00	0	4.04E+00	0	Single admission	12
2012	0	7.52E+00	1	1.67E+00	0	Single admission	12
2012	0	7.51E+00	1	4.73E-01	0	Repeated admissions	12
2012	1	7.51E+00	0	4.83E+00	0	Repeated admissions	12
2012	1	7.50E+00	0	3.93E+00	0	Single admission	12
2012	1	7.49E+00	0	9.66E-01	0	Single admission	12
2012	0	7.49E+00	1	5.91E-01	0	Single admission	12
2012	1	7.49E+00	0	6.29E+00	0	Single admission	12
2012	0	7.48E+00	1	2.81E-01	0	Single admission	12
2012	1	7.48E+00	1	3.88E+00	0	Single admission	12
2013	1	7.43E+00	1	8.05E-01	0	Single admission	1
2013	15	7.30E+00	0	2.97E+00	0	Single admission	1
2013	0	6.89E+00	0	1.41E+00	0	Single admission	1
2013	6	6.73E+00	0	4.29E+00	0	Single admission	1
2013	1	6.71E+00	0	1.90E-01	0	Single admission	1
2013	1	6.64E+00	1	1.15E+00	0	Single admission	1
2013	1	7.46E+00	0	5.72E+00	0	Repeated admissions	2
2013	0	7.30E+00	1	1.02E+00	0	Single admission	2
2013	2	7.20E+00	1	4.52E-01	0	Repeated admissions	2
2013	0	7.19E+00	0	1.78E+01	0	Single admission	2
2013	2	7.17E+00	1	1.67E-01	0	Repeated admissions	2

Admission year	Age (years)	Follow up time (years)	Sex (Females=1)	PDRLast	Outcome (Deceased=1)	Single/repeated admission	Diagnostic group
2013	1	7.14E+00	1	4.08E+00	0	Repeated admissions	2
2013	0	7.10E+00	1	2.98E-01	0	Single admission	2
2013	0	7.09E+00	0	1.54E+00	0	Single admission	2
2013	0	7.03E+00	1	2.77E+00	0	Single admission	2
2013	0	6.86E+00	1	9.53E+00	0	Single admission	2
2013	0	6.85E+00	0	6.22E+00	0	Single admission	2
2013	0	6.82E+00	0	1.29E+00	0	Single admission	2
2013	0	6.77E+00	1	9.64E+00	0	Single admission	2
2013	0	6.75E+00	1	4.36E+00	0	Single admission	2
2013	0	6.72E+00	0	1.32E+00	0	Repeated admissions	2
2013	0	6.67E+00	0	1.30E+00	0	Single admission	2
2013	0	6.60E+00	1	6.48E+00	0	Single admission	2
2013	0	6.57E+00	1	1.23E+01	0	Single admission	2
2013	0	6.53E+00	1	1.15E+00	0	Single admission	2
2013	0	6.48E+00	1	7.62E+00	0	Single admission	2
2013	0	7.37E+00	0	1.67E-01	0	Repeated admissions	3
2013	0	7.27E+00	0	2.04E-01	0	Single admission	3
2013	1	7.21E+00	0	1.34E+00	0	Single admission	3
2013	0	3.25E+00	1	2.94E+00	0	Single admission	3
2013	0	7.03E+00	0	3.75E+00	0	Single admission	3
2013	0	6.99E+00	1	5.35E-01	0	Single admission	3
2013	2	6.97E+00	0	2.97E-02	0	Repeated admissions	3
2013	0	6.94E+00	1	2.70E+00	0	Single admission	3
2013	0	6.93E+00	0	2.71E-01	0	Single admission	3
2013	2	6.82E+00	1	4.30E-01	0	Repeated admissions	3
2013	1	6.81E+00	1	6.00E-01	0	Single admission	3
2013	10	6.80E+00	1	1.92E-01	0	Single admission	3
2013	6	6.65E+00	0	7.63E+00	0	Single admission	3
2013	4	6.62E+00	0	4.81E+00	0	Single admission	3
2013	5	6.54E+00	0	1.14E+00	0	Repeated admissions	3

Admission year	Age (years)	Follow up time (years)	Sex (Females=1)	PDRLast	Outcome (Deceased=1)	Single/repeated admission	Diagnostic group
2013	7	8.06E-03	1	7.99E-01	1	Single admission	3
2013	11	6.51E+00	1	5.03E+00	0	Repeated admissions	3
2013	0	6.51E+00	0	1.58E+01	0	Single admission	3
2013	4	7.40E+00	1	4.89E+00	0	Single admission	4
2013	4	7.39E+00	1	1.00E+01	0	Single admission	4
2013	14	7.38E+00	0	6.08E+00	0	Single admission	4
2013	0	7.35E+00	0	2.00E+00	0	Single admission	4
2013	2	7.33E+00	0	6.70E+00	0	Single admission	4
2013	2	7.31E+00	0	1.56E-01	0	Repeated admissions	4
2013	4	7.29E+00	1	1.00E+01	0	Single admission	4
2013	1	7.25E+00	0	3.72E+00	0	Single admission	4
2013	1	1.80E-03	1	1.66E+01	1	Single admission	4
2013	0	7.20E+00	1	3.89E+01	0	Single admission	4
2013	7	7.20E+00	0	1.31E+00	0	Single admission	4
2013	0	7.19E+00	0	9.55E+00	0	Single admission	4
2013	5	7.11E+00	1	4.25E+00	0	Single admission	4
2013	0	7.06E+00	0	9.47E+00	0	Single admission	4
2013	12	7.06E+00	1	5.07E+00	0	Single admission	4
2013	0	7.00E+00	0	9.83E-01	0	Single admission	4
2013	0	6.97E+00	0	1.20E+00	0	Single admission	4
2013	0	6.97E+00	1	1.48E+00	0	Single admission	4
2013	10	6.97E+00	1	8.66E+00	0	Single admission	4
2013	0	6.95E+00	1	3.88E+00	0	Single admission	4
2013	0	6.91E+00	1	1.22E+00	0	Single admission	4
2013	0	6.72E+00	0	7.51E-01	0	Single admission	4
2013	0	6.71E+00	0	1.80E+00	0	Single admission	4
2013	10	6.61E+00	1	1.50E+00	0	Single admission	4
2013	2	6.57E+00	1	1.13E+00	0	Single admission	4
2013	0	6.56E+00	1	8.13E-01	0	Repeated admissions	4
2013	0	6.49E+00	0	1.70E+00	0	Single admission	4

Admission year	Age (years)	Follow up time (years)	Sex (Females=1)	PDRLast	Outcome (Deceased=1)	Single/repeated admission	Diagnostic group
2013	4	7.43E+00	1	7.51E-01	0	Single admission	5
2013	4	7.32E+00	0	1.30E-01	0	Repeated admissions	5
2013	3	7.29E+00	0	2.00E+01	0	Single admission	5
2013	12	7.26E+00	0	3.43E-01	0	Single admission	5
2013	2	7.16E+00	0	1.73E-01	0	Single admission	5
2013	1	7.09E+00	0	1.33E+00	0	Single admission	5
2013	5	6.98E+00	1	5.11E+00	0	Single admission	5
2013	5	6.95E+00	1	2.86E-01	0	Single admission	5
2013	15	6.93E+00	0	3.97E+00	0	Single admission	5
2013	11	6.91E+00	1	1.57E-01	0	Single admission	5
2013	3	6.90E+00	0	4.64E+00	0	Single admission	5
2013	1	6.80E+00	1	4.22E-01	0	Single admission	5
2013	4	6.79E+00	1	1.48E-01	0	Single admission	5
2013	1	6.77E+00	0	2.08E+00	0	Repeated admissions	5
2013	17	6.70E+00	1	7.02E+00	0	Single admission	5
2013	3	3.15E-01	1	3.41E-01	1	Single admission	5
2013	2	6.66E+00	0	1.63E-01	0	Single admission	5
2013	9	6.66E+00	1	7.51E-01	0	Single admission	5
2013	2	6.61E+00	1	3.20E-01	0	Repeated admissions	5
2013	2	6.55E+00	0	1.00E+00	0	Single admission	5
2013	0	6.53E+00	0	2.23E+00	0	Single admission	5
2013	0	7.27E-02	0	9.76E+01	1	Single admission	6
2013	12	7.45E+00	0	5.18E-01	0	Single admission	6
2013	15	7.43E+00	1	4.18E+00	0	Single admission	6
2013	9	7.41E+00	0	6.34E+00	0	Single admission	6
2013	6	7.38E+00	0	4.35E+00	0	Single admission	6
2013	0	7.37E+00	1	7.39E+00	0	Single admission	6
2013	2	7.30E+00	1	2.22E-02	0	Repeated admissions	6
2013	0	5.02E-03	0	7.52E+01	1	Single admission	6
2013	5	7.25E+00	1	1.08E+01	0	Single admission	6

Admission year	Age (years)	Follow up time (years)	Sex (Females=1)	PDRLast	Outcome (Deceased=1)	Single/repeated admission	Diagnostic group
2013	0	7.21E+00	0	1.76E+00	0	Single admission	6
2013	12	7.15E+00	1	9.59E+00	0	Single admission	6
2013	0	7.13E+00	0	5.11E+00	0	Single admission	6
2013	4	4.40E+00	0	5.82E+00	1	Repeated admissions	6
2013	0	7.01E+00	0	1.77E+00	0	Single admission	6
2013	0	2.56E-02	1	3.26E+01	1	Single admission	6
2013	0	6.94E+00	1	7.09E+00	0	Single admission	6
2013	0	6.91E+00	1	7.51E-01	0	Single admission	6
2013	1	6.89E+00	0	4.40E-01	0	Single admission	6
2013	0	6.87E+00	1	3.88E+00	0	Single admission	6
2013	0	6.11E+00	1	2.00E+00	0	Single admission	6
2013	17	6.82E+00	0	4.90E+00	0	Single admission	6
2013	1	6.79E+00	0	2.24E+00	0	Repeated admissions	6
2013	2	3.06E-02	0	3.92E+01	1	Single admission	6
2013	0	6.78E+00	0	5.04E+00	0	Single admission	6
2013	0	6.67E+00	0	1.34E+00	0	Single admission	6
2013	9	5.35E-01	0	1.22E+00	1	Repeated admissions	6
2013	7	6.62E+00	1	4.78E+00	0	Single admission	6
2013	3	6.61E+00	0	2.78E+00	0	Repeated admissions	6
2013	3	6.60E+00	1	8.99E-01	0	Single admission	6
2013	0	3.48E-01	1	5.93E+00	1	Repeated admissions	6
2013	0	8.51E-03	0	6.24E+01	1	Single admission	6
2013	2	6.51E+00	1	2.33E+01	0	Repeated admissions	6
2013	10	6.50E+00	1	1.20E+00	0	Single admission	6
2013	18	7.46E+00	0	1.04E+01	0	Single admission	7
2013	8	7.41E+00	0	1.22E+00	0	Single admission	7
2013	6	7.38E+00	0	7.59E-01	0	Single admission	7
2013	11	7.32E+00	0	1.40E+00	0	Single admission	7
2013	16	7.28E+00	0	1.39E+00	0	Single admission	7
2013	14	7.26E+00	0	2.09E+00	0	Single admission	7

Admission year	Age (years)	Follow up time (years)	Sex (Females=1)	PDRLast	Outcome (Deceased=1)	Single/repeated admission	Diagnostic group
2013	10	7.24E+00	1	1.66E+00	0	Single admission	7
2013	1	7.21E+00	0	9.02E+00	0	Single admission	7
2013	0	7.18E+00	1	8.87E+01	0	Single admission	7
2013	12	7.12E+00	1	1.03E+01	0	Single admission	7
2013	15	7.11E+00	0	5.65E-01	0	Single admission	7
2013	5	7.06E+00	1	1.34E+00	0	Single admission	7
2013	0	7.05E+00	0	4.33E+00	0	Single admission	7
2013	14	7.04E+00	0	1.17E+01	0	Single admission	7
2013	2	7.03E+00	1	5.03E+00	0	Single admission	7
2013	1	7.02E+00	0	2.89E-01	0	Single admission	7
2013	0	4.77E-01	0	3.10E+00	1	Repeated admissions	7
2013	0	6.94E+00	1	1.37E+00	0	Single admission	7
2013	2	6.93E+00	0	9.76E-01	0	Single admission	7
2013	3	6.92E+00	0	7.84E-01	0	Single admission	7
2013	1	6.90E+00	1	3.10E+00	0	Single admission	7
2013	12	6.89E+00	1	1.35E+00	0	Single admission	7
2013	17	6.88E+00	1	1.42E+00	0	Repeated admissions	7
2013	9	6.87E+00	0	1.25E+00	0	Single admission	7
2013	1	6.86E+00	1	4.08E+00	0	Single admission	7
2013	13	6.79E+00	0	7.40E+00	0	Single admission	7
2013	17	6.77E+00	0	1.47E-01	0	Single admission	7
2013	16	6.75E+00	1	2.17E+00	0	Single admission	7
2013	15	6.74E+00	0	4.59E+00	0	Repeated admissions	7
2013	17	6.73E+00	1	5.14E+00	0	Single admission	7
2013	1	6.68E+00	0	6.71E-01	0	Single admission	7
2013	13	6.68E+00	0	2.30E+00	0	Single admission	7
2013	16	6.66E+00	1	2.82E+00	0	Single admission	7
2013	2	6.60E+00	0	2.33E+00	0	Single admission	7
2013	10	6.55E+00	0	8.96E-01	0	Single admission	7
2013	16	6.55E+00	1	5.42E-01	0	Single admission	7

Admission year	Age (years)	Follow up time (years)	Sex (Females=1)	PDRLast	Outcome (Deceased=1)	Single/repeated admission	Diagnostic group
2013	6	6.53E+00	1	1.94E+00	0	Single admission	7
2013	8	6.48E+00	1	4.39E+00	0	Single admission	7
2013	6	7.45E+00	0	8.62E-01	0	Single admission	8
2013	9	7.44E+00	1	3.37E+00	0	Single admission	8
2013	12	7.44E+00	0	7.51E-01	0	Single admission	8
2013	3	7.31E+00	0	7.51E-01	0	Single admission	8
2013	2	3.69E+00	0	1.40E+00	0	Single admission	8
2013	1	7.30E+00	0	1.12E+00	0	Single admission	8
2013	14	7.28E+00	0	7.51E-01	0	Single admission	8
2013	12	7.27E+00	1	5.60E-01	0	Single admission	8
2013	0	7.25E+00	1	6.62E+00	0	Single admission	8
2013	12	7.24E+00	1	8.62E-01	0	Single admission	8
2013	0	7.22E+00	1	3.11E+00	0	Single admission	8
2013	17	7.17E+00	0	1.98E+01	0	Single admission	8
2013	3	5.49E-03	1	4.75E+01	1	Single admission	8
2013	7	7.13E+00	1	2.19E+00	0	Single admission	8
2013	0	7.07E+00	0	7.51E-01	0	Single admission	8
2013	18	7.04E+00	1	2.66E+00	0	Single admission	8
2013	5	7.04E+00	0	3.28E+00	0	Single admission	8
2013	7	7.04E+00	0	7.51E-01	0	Single admission	8
2013	17	7.04E+00	1	7.61E-01	0	Single admission	8
2013	10	7.03E+00	0	7.51E-01	0	Single admission	8
2013	1	7.03E+00	0	7.51E-01	0	Single admission	8
2013	9	7.02E+00	1	9.24E-01	0	Single admission	8
2013	2	7.01E+00	0	7.51E-01	0	Single admission	8
2013	5	7.00E+00	1	1.71E+00	0	Single admission	8
2013	15	6.99E+00	1	9.90E-01	0	Single admission	8
2013	0	2.85E+00	1	1.87E+00	1	Repeated admissions	8
2013	1	6.97E+00	0	1.31E+00	0	Single admission	8
2013	6	6.97E+00	1	1.03E+00	0	Single admission	8

Admission year	Age (years)	Follow up time (years)	Sex (Females=1)	PDRLast	Outcome (Deceased=1)	Single/repeated admission	Diagnostic group
2013	5	6.95E+00	0	2.90E-01	0	Single admission	8
2013	16	6.94E+00	1	3.88E+00	0	Single admission	8
2013	5	6.94E+00	0	3.86E-01	0	Single admission	8
2013	13	6.92E+00	0	7.51E-01	0	Single admission	8
2013	14	6.92E+00	1	9.56E-01	0	Single admission	8
2013	1	6.91E+00	1	7.51E-01	0	Single admission	8
2013	3	6.91E+00	1	1.48E+00	0	Single admission	8
2013	4	6.90E+00	0	1.65E+00	0	Single admission	8
2013	17	6.88E+00	1	9.74E-01	0	Single admission	8
2013	14	6.87E+00	0	9.90E-01	0	Single admission	8
2013	1	6.85E+00	1	1.82E+00	0	Single admission	8
2013	7	6.83E+00	0	8.21E-01	0	Single admission	8
2013	12	6.83E+00	0	3.89E+00	0	Single admission	8
2013	13	6.83E+00	0	1.38E+00	0	Single admission	8
2013	7	6.82E+00	0	7.72E-01	0	Single admission	8
2013	17	6.81E+00	1	8.05E-01	0	Repeated admissions	8
2013	4	6.80E+00	0	9.77E-01	0	Single admission	8
2013	10	6.79E+00	0	3.18E+00	0	Single admission	8
2013	13	6.78E+00	1	1.04E+00	0	Single admission	8
2013	16	6.78E+00	1	7.72E-01	0	Single admission	8
2013	6	6.73E+00	0	4.74E+00	0	Single admission	8
2013	7	6.69E+00	1	1.29E+00	0	Single admission	8
2013	0	9.22E-03	0	8.82E+01	1	Single admission	8
2013	14	6.67E+00	0	7.94E-01	0	Single admission	8
2013	0	6.67E+00	0	9.33E-01	0	Repeated admissions	8
2013	17	6.66E+00	1	1.20E+00	0	Single admission	8
2013	1	6.64E+00	0	1.98E+00	0	Single admission	8
2013	14	6.63E+00	0	8.62E-01	0	Single admission	8
2013	0	6.61E+00	0	1.45E+00	0	Single admission	8
2013	4	6.58E+00	0	1.60E+00	0	Repeated admissions	8

Admission year	Age (years)	Follow up time (years)	Sex (Females=1)	PDRLast	Outcome (Deceased=1)	Single/repeated admission	Diagnostic group
2013	14	6.57E+00	1	1.90E+00	0	Single admission	8
2013	18	6.57E+00	1	9.90E-01	0	Single admission	8
2013	2	6.55E+00	0	4.73E+00	0	Single admission	8
2013	12	6.49E+00	0	3.86E+00	0	Single admission	8
2013	12	7.43E+00	1	2.17E-01	0	Single admission	9
2013	0	7.33E+00	1	9.01E-01	0	Single admission	9
2013	17	4.84E+00	0	1.43E-01	1	Single admission	9
2013	9	7.27E+00	0	7.24E-01	0	Single admission	9
2013	0	7.26E+00	1	7.60E-01	0	Single admission	9
2013	5	7.25E+00	0	2.12E+00	0	Repeated admissions	9
2013	7	7.25E+00	1	1.64E+01	0	Repeated admissions	9
2013	0	7.25E+00	0	1.58E-01	0	Single admission	9
2013	0	7.21E+00	0	1.70E+00	0	Single admission	9
2013	0	7.20E+00	0	1.17E+00	0	Single admission	9
2013	0	7.20E+00	0	1.56E-01	0	Repeated admissions	9
2013	6	7.13E+00	1	1.48E-01	0	Single admission	9
2013	10	7.11E+00	1	4.05E-01	0	Repeated admissions	9
2013	7	7.11E+00	0	2.43E-01	0	Single admission	9
2013	10	7.11E+00	1	1.87E-01	0	Single admission	9
2013	13	7.10E+00	0	1.34E-01	0	Single admission	9
2013	5	7.10E+00	0	2.99E-01	0	Single admission	9
2013	3	7.09E+00	1	2.30E-01	0	Single admission	9
2013	12	8.22E-01	0	1.03E+00	1	Repeated admissions	9
2013	10	7.06E+00	0	6.62E-01	0	Single admission	9
2013	0	7.06E+00	0	1.43E+00	0	Single admission	9
2013	16	7.02E+00	0	2.71E-01	0	Single admission	9
2013	0	7.02E+00	0	4.05E-01	0	Single admission	9
2013	1	6.95E+00	1	9.50E-01	0	Single admission	9
2013	0	6.95E+00	0	2.15E-01	0	Single admission	9
2013	1	6.91E+00	0	2.97E-01	0	Single admission	9

Admission year	Age (years)	Follow up time (years)	Sex (Females=1)	PDRLast	Outcome (Deceased=1)	Single/repeated admission	Diagnostic group
2013	3	6.88E+00	0	5.90E-01	0	Repeated admissions	9
2013	0	6.87E+00	0	2.47E-01	0	Single admission	9
2013	16	6.78E+00	1	1.48E+00	0	Repeated admissions	9
2013	14	6.76E+00	1	2.40E-01	0	Single admission	9
2013	0	6.75E+00	1	4.59E-01	0	Single admission	9
2013	4	6.74E+00	0	2.09E-01	0	Single admission	9
2013	12	6.68E+00	1	1.78E+00	0	Single admission	9
2013	3	6.67E+00	0	1.31E+00	0	Repeated admissions	9
2013	0	6.65E+00	0	8.22E-01	0	Single admission	9
2013	2	6.61E+00	0	1.23E+00	0	Single admission	9
2013	0	6.60E+00	0	1.59E+00	0	Single admission	9
2013	14	1.38E+00	1	1.68E-01	1	Repeated admissions	9
2013	0	6.59E+00	0	2.71E-01	0	Single admission	9
2013	0	6.57E+00	0	4.52E-01	0	Single admission	9
2013	12	6.57E+00	1	1.07E-01	0	Single admission	9
2013	6	9.13E-01	1	1.90E-01	1	Repeated admissions	9
2013	11	6.54E+00	0	1.57E-01	0	Single admission	9
2013	1	6.51E+00	1	3.34E-01	0	Single admission	9
2013	5	7.46E+00	1	1.32E+00	0	Single admission	10
2013	1	7.44E+00	1	1.72E+00	0	Single admission	10
2013	0	7.44E+00	0	1.31E+01	0	Single admission	10
2013	1	7.42E+00	1	7.51E-01	0	Single admission	10
2013	2	7.41E+00	0	2.18E+00	0	Single admission	10
2013	6	7.41E+00	0	4.08E+00	0	Single admission	10
2013	0	7.40E+00	0	3.66E+00	0	Repeated admissions	10
2013	3	2.94E+00	0	2.45E+00	1	Repeated admissions	10
2013	1	7.31E+00	1	1.51E+00	0	Single admission	10
2013	0	3.84E-02	0	7.51E-01	1	Single admission	10
2013	18	7.29E+00	1	3.89E+00	0	Repeated admissions	10
2013	0	1.19E+00	1	1.08E+00	1	Repeated admissions	10

Admission year	Age (years)	Follow up time (years)	Sex (Females=1)	PDRLast	Outcome (Deceased=1)	Single/repeated admission	Diagnostic group
2013	2	7.27E+00	0	7.51E-01	0	Single admission	10
2013	0	7.24E+00	1	9.90E-01	0	Single admission	10
2013	1	9.05E-01	0	2.99E+00	1	Single admission	10
2013	8	6.79E+00	0	7.51E-01	1	Repeated admissions	10
2013	1	7.21E+00	1	1.40E+00	0	Single admission	10
2013	11	7.12E+00	0	7.51E-01	0	Single admission	10
2013	8	7.10E+00	1	4.57E+00	0	Single admission	10
2013	12	6.25E-01	0	3.21E-01	1	Repeated admissions	10
2013	3	7.06E+00	0	1.13E+00	0	Single admission	10
2013	2	7.05E+00	1	1.66E+00	0	Single admission	10
2013	0	7.04E+00	1	7.51E-01	0	Single admission	10
2013	6	6.98E+00	1	9.90E-01	0	Repeated admissions	10
2013	3	2.73E+00	0	3.26E+01	1	Single admission	10
2013	8	6.94E+00	0	1.02E+00	0	Single admission	10
2013	13	6.90E+00	0	3.65E+00	0	Single admission	10
2013	4	6.88E+00	1	1.06E+00	0	Single admission	10
2013	1	6.87E+00	0	1.08E+00	0	Single admission	10
2013	2	6.83E+00	1	9.11E-01	0	Single admission	10
2013	5	6.77E+00	1	6.73E+00	0	Single admission	10
2013	3	6.77E+00	0	7.51E-01	0	Repeated admissions	10
2013	4	6.73E+00	1	2.13E+00	0	Single admission	10
2013	3	6.72E+00	0	1.14E+00	0	Single admission	10
2013	8	6.71E+00	0	4.41E-01	0	Single admission	10
2013	14	6.70E+00	1	2.22E+00	0	Repeated admissions	10
2013	2	6.69E+00	1	2.02E+00	0	Single admission	10
2013	2	6.68E+00	0	1.85E+00	0	Repeated admissions	10
2013	0	6.66E+00	0	2.80E+00	0	Single admission	10
2013	3	6.65E+00	0	1.31E+00	0	Single admission	10
2013	2	6.63E+00	1	5.87E+00	0	Single admission	10
2013	10	6.60E+00	1	1.49E-01	0	Single admission	10

Admission year	Age (years)	Follow up time (years)	Sex (Females=1)	PDRLast	Outcome (Deceased=1)	Single/repeated admission	Diagnostic group
2013	2	6.60E+00	1	4.13E+00	0	Single admission	10
2013	4	6.59E+00	1	9.94E-01	0	Single admission	10
2013	1	6.59E+00	1	1.07E-01	0	Single admission	10
2013	13	6.58E+00	1	5.51E-02	0	Repeated admissions	10
2013	11	6.55E+00	1	1.41E+00	0	Single admission	10
2013	0	6.54E+00	1	7.51E-01	0	Single admission	10
2013	8	6.53E+00	1	3.60E+00	0	Single admission	10
2013	1	6.51E+00	0	2.60E+00	0	Single admission	10
2013	14	1.40E-01	0	1.74E+01	1	Repeated admissions	10
2013	2	6.48E+00	1	4.30E+00	0	Single admission	10
2013	0	7.46E+00	1	1.74E+00	0	Single admission	11
2013	0	7.45E+00	1	2.79E+01	0	Single admission	11
2013	0	7.38E+00	0	1.74E+00	0	Single admission	11
2013	14	7.33E+00	1	5.85E-01	0	Single admission	11
2013	2	7.32E+00	0	7.18E-01	0	Single admission	11
2013	0	7.32E+00	0	1.05E+00	0	Single admission	11
2013	0	7.30E+00	0	1.26E+00	0	Single admission	11
2013	0	7.30E+00	0	5.76E+00	0	Single admission	11
2013	2	7.29E+00	1	1.33E+00	0	Single admission	11
2013	0	7.28E+00	0	5.84E+00	0	Single admission	11
2013	0	1.68E-01	1	1.23E+01	1	Repeated admissions	11
2013	16	7.26E+00	1	2.57E-01	0	Single admission	11
2013	0	7.22E+00	0	1.44E+00	0	Repeated admissions	11
2013	0	7.21E+00	1	5.39E+00	0	Repeated admissions	11
2013	0	7.20E+00	0	1.24E+00	0	Single admission	11
2013	0	7.18E+00	0	7.51E-01	0	Single admission	11
2013	0	7.18E+00	1	5.66E+00	0	Single admission	11
2013	0	7.13E+00	1	3.81E+00	0	Single admission	11
2013	0	7.11E+00	0	4.22E+01	0	Single admission	11
2013	0	7.09E+00	0	4.72E-01	0	Repeated admissions	11

Admission year	Age (years)	Follow up time (years)	Sex (Females=1)	PDRLast	Outcome (Deceased=1)	Single/repeated admission	Diagnostic group
2013	0	7.08E+00	0	1.07E-01	0	Repeated admissions	11
2013	0	7.07E+00	0	1.08E+00	0	Single admission	11
2013	0	7.07E+00	0	2.82E+00	0	Single admission	11
2013	0	7.06E+00	0	1.81E+00	0	Single admission	11
2013	0	7.01E+00	0	2.52E+00	0	Single admission	11
2013	3	7.01E+00	0	1.58E+00	0	Repeated admissions	11
2013	0	7.01E+00	0	6.19E+00	0	Repeated admissions	11
2013	0	7.00E+00	0	1.30E+01	0	Single admission	11
2013	0	6.99E+00	0	2.16E+00	0	Single admission	11
2013	0	6.91E+00	1	2.98E+00	0	Repeated admissions	11
2013	0	6.89E+00	0	3.58E-01	0	Single admission	11
2013	0	6.89E+00	0	5.69E-01	0	Single admission	11
2013	0	6.88E+00	0	9.86E+00	0	Single admission	11
2013	0	6.86E+00	0	7.94E+00	0	Single admission	11
2013	0	6.84E+00	1	1.52E-01	0	Repeated admissions	11
2013	0	6.84E+00	1	1.64E+00	0	Repeated admissions	11
2013	0	6.83E+00	1	2.98E-01	0	Single admission	11
2013	0	1.13E+00	0	1.06E+00	0	Single admission	11
2013	0	6.81E+00	0	1.29E+00	0	Single admission	11
2013	0	6.81E+00	1	3.10E+00	0	Repeated admissions	11
2013	6	6.79E+00	1	2.10E+00	0	Single admission	11
2013	0	6.78E+00	1	1.57E+00	0	Single admission	11
2013	0	6.78E+00	0	2.98E-01	0	Single admission	11
2013	0	6.76E+00	0	6.30E+00	0	Repeated admissions	11
2013	0	9.08E-01	1	6.01E-01	1	Single admission	11
2013	0	6.72E+00	0	7.05E-01	0	Single admission	11
2013	1	6.68E+00	0	1.39E+00	0	Single admission	11
2013	0	6.67E+00	1	3.52E-01	0	Single admission	11
2013	0	5.96E+00	1	7.28E+00	0	Single admission	11
2013	0	6.64E+00	0	9.11E-01	0	Repeated admissions	11

Admission year	Age (years)	Follow up time (years)	Sex (Females=1)	PDRLast	Outcome (Deceased=1)	Single/repeated admission	Diagnostic group
2013	0	6.62E+00	0	1.46E+00	0	Single admission	11
2013	0	6.61E+00	1	4.16E+00	0	Single admission	11
2013	0	6.57E+00	1	2.50E-01	0	Single admission	11
2013	0	6.55E+00	0	2.73E+00	0	Single admission	11
2013	1	6.53E+00	0	7.59E-01	0	Repeated admissions	11
2013	0	6.51E+00	1	8.29E-01	0	Repeated admissions	11
2013	3	7.47E+00	1	7.51E-01	0	Single admission	12
2013	0	7.47E+00	1	1.02E+00	0	Repeated admissions	12
2013	0	7.47E+00	0	1.12E+01	0	Single admission	12
2013	0	7.47E+00	0	8.93E-01	0	Single admission	12
2013	1	7.47E+00	1	4.31E+00	0	Single admission	12
2013	0	7.46E+00	0	1.76E+00	0	Single admission	12
2013	0	7.46E+00	1	5.43E+00	0	Single admission	12
2013	0	7.46E+00	0	1.22E+00	0	Single admission	12
2013	2	7.45E+00	0	2.69E+00	0	Single admission	12
2013	4	7.43E+00	1	1.16E+01	0	Repeated admissions	12
2013	0	7.42E+00	1	5.69E+00	0	Repeated admissions	12
2013	2	7.42E+00	0	1.57E+00	0	Single admission	12
2013	1	7.41E+00	0	1.26E+00	0	Single admission	12
2013	2	7.41E+00	1	7.94E+00	0	Single admission	12
2013	1	7.41E+00	0	2.62E-01	0	Single admission	12
2013	0	7.41E+00	1	4.21E-01	0	Single admission	12
2013	0	7.40E+00	0	1.42E+00	0	Single admission	12
2013	0	2.74E-03	1	9.59E+01	1	Repeated admissions	12
2013	0	7.40E+00	1	6.51E-01	0	Single admission	12
2013	0	7.40E+00	0	1.03E+00	0	Single admission	12
2013	0	7.40E+00	0	4.23E+00	0	Single admission	12
2013	0	7.39E+00	1	2.07E+00	0	Single admission	12
2013	12	7.39E+00	0	8.62E-01	0	Single admission	12
2013	0	7.39E+00	0	8.65E+00	0	Repeated admissions	12

Admission year	Age (years)	Follow up time (years)	Sex (Females=1)	PDRLast	Outcome (Deceased=1)	Single/repeated admission	Diagnostic group
2013	0	7.38E+00	1	2.44E-01	0	Single admission	12
2013	0	7.38E+00	1	2.47E-01	0	Single admission	12
2013	0	7.38E+00	0	1.71E+00	0	Single admission	12
2013	0	7.38E+00	1	1.56E-01	0	Single admission	12
2013	0	7.38E+00	1	6.48E+00	0	Repeated admissions	12
2013	0	7.38E+00	1	9.89E-01	0	Single admission	12
2013	0	7.37E+00	1	2.07E+01	0	Single admission	12
2013	0	7.37E+00	1	9.17E-01	0	Single admission	12
2013	0	4.41E-02	1	9.32E+01	1	Repeated admissions	12
2013	0	7.36E+00	0	2.20E+00	0	Single admission	12
2013	0	7.36E+00	1	3.58E-01	0	Single admission	12
2013	0	7.36E+00	0	1.90E+00	0	Single admission	12
2013	0	7.36E+00	1	1.88E+00	0	Single admission	12
2013	0	7.35E+00	1	1.13E+00	0	Single admission	12
2013	1	7.35E+00	0	3.14E-01	0	Single admission	12
2013	1	7.35E+00	1	9.85E-01	0	Repeated admissions	12
2013	0	7.35E+00	1	9.90E-01	0	Single admission	12
2013	4	7.34E+00	1	5.92E+00	0	Single admission	12
2013	0	7.34E+00	1	8.82E-01	0	Single admission	12
2013	0	7.34E+00	0	2.91E+00	0	Single admission	12
2013	3	7.34E+00	1	1.69E+00	0	Repeated admissions	12
2013	1	7.34E+00	0	8.53E-01	0	Single admission	12
2013	0	7.34E+00	1	8.27E-01	0	Single admission	12
2013	3	7.33E+00	1	1.83E+00	0	Repeated admissions	12
2013	0	7.33E+00	0	2.44E+00	0	Single admission	12
2013	3	7.33E+00	1	1.16E+00	0	Single admission	12
2013	0	7.32E+00	1	3.90E+00	0	Single admission	12
2013	12	7.32E+00	0	7.51E-01	0	Repeated admissions	12
2013	5	7.31E+00	0	1.56E-01	0	Single admission	12
2013	3	7.31E+00	0	2.22E-01	0	Single admission	12

Admission year	Age (years)	Follow up time (years)	Sex (Females=1)	PDRLast	Outcome (Deceased=1)	Single/repeated admission	Diagnostic group
2013	2	7.31E+00	0	1.26E+00	0	Single admission	12
2013	1	7.29E+00	0	8.64E-01	0	Single admission	12
2013	1	7.28E+00	1	2.23E+00	0	Repeated admissions	12
2013	0	7.27E+00	1	3.88E+00	0	Single admission	12
2013	0	7.26E+00	0	7.17E+00	0	Repeated admissions	12
2013	5	7.25E+00	0	3.23E-01	0	Single admission	12
2013	2	7.25E+00	1	1.84E-01	0	Single admission	12
2013	8	7.24E+00	1	2.80E+00	0	Repeated admissions	12
2013	5	7.24E+00	0	6.25E+00	0	Repeated admissions	12
2013	1	5.08E+00	0	8.49E+00	0	Single admission	12
2013	0	7.23E+00	0	1.45E+00	0	Single admission	12
2013	0	7.23E+00	0	3.55E-01	0	Repeated admissions	12
2013	0	7.23E+00	1	1.56E-01	0	Single admission	12
2013	1	7.22E+00	0	8.39E-01	0	Single admission	12
2013	1	7.21E+00	1	3.88E+00	0	Single admission	12
2013	11	7.18E+00	1	3.88E+00	0	Single admission	12
2013	2	7.16E+00	0	1.56E-01	0	Single admission	12
2013	0	7.16E+00	0	2.80E+00	0	Single admission	12
2013	2	7.16E+00	1	1.39E+00	0	Single admission	12
2013	12	7.12E+00	1	1.98E+00	0	Repeated admissions	12
2013	1	7.10E+00	1	6.04E+01	0	Single admission	12
2013	0	7.09E+00	1	1.07E-01	0	Repeated admissions	12
2013	2	7.09E+00	1	4.71E-01	0	Single admission	12
2013	0	7.09E+00	0	2.71E-01	0	Single admission	12
2013	0	7.05E+00	0	5.43E+00	0	Single admission	12
2013	0	7.04E+00	0	6.86E+00	0	Single admission	12
2013	1	7.03E+00	0	5.22E+00	0	Repeated admissions	12
2013	1	6.99E+00	1	4.77E+00	0	Single admission	12
2013	1	7.02E-01	0	8.51E-01	1	Repeated admissions	12
2013	0	2.10E-03	1	9.91E+01	1	Single admission	12

Admission year	Age (years)	Follow up time (years)	Sex (Females=1)	PDRLast	Outcome (Deceased=1)	Single/repeated admission	Diagnostic group
2013	0	6.94E+00	1	5.91E-01	0	Single admission	12
2013	0	6.94E+00	0	8.30E+00	0	Repeated admissions	12
2013	0	6.89E+00	0	1.69E-01	0	Single admission	12
2013	0	6.89E+00	1	1.82E+00	0	Single admission	12
2013	3	6.85E+00	0	3.83E-01	0	Repeated admissions	12
2013	1	6.84E+00	0	2.20E-01	0	Repeated admissions	12
2013	1	5.60E-02	1	9.07E+00	1	Single admission	12
2013	0	1.09E-01	0	3.38E+00	1	Repeated admissions	12
2013	3	6.82E+00	0	1.02E+00	0	Single admission	12
2013	12	6.82E+00	0	1.07E-01	0	Single admission	12
2013	3	6.79E+00	1	1.12E+00	0	Single admission	12
2013	0	6.03E-03	1	5.22E+01	1	Single admission	12
2013	1	6.76E+00	1	2.83E+00	0	Single admission	12
2013	2	6.74E+00	0	2.75E-01	0	Repeated admissions	12
2013	0	6.73E+00	0	2.10E+00	0	Single admission	12
2013	2	6.71E+00	0	5.61E-02	0	Single admission	12
2013	2	2.74E-04	1	1.62E+01	1	Single admission	12
2013	3	6.71E+00	1	5.60E+00	0	Single admission	12
2013	3	6.70E+00	0	2.30E-01	0	Single admission	12
2013	0	6.66E+00	0	2.58E-01	0	Single admission	12
2013	5	6.66E+00	0	8.62E-01	0	Single admission	12
2013	1	6.64E+00	0	2.19E-01	0	Single admission	12
2013	8	6.64E+00	1	7.51E-01	0	Single admission	12
2013	7	6.63E+00	1	1.06E+00	0	Single admission	12
2013	2	6.61E+00	0	2.80E+00	0	Repeated admissions	12
2013	2	6.61E+00	0	1.36E-01	0	Repeated admissions	12
2013	3	6.59E+00	0	2.36E+00	0	Repeated admissions	12
2013	13	6.59E+00	0	1.35E+00	0	Single admission	12
2013	2	1.42E+00	0	3.65E+01	1	Repeated admissions	12
2013	0	6.59E+00	0	7.51E-01	0	Single admission	12

Admission year	Age (years)	Follow up time (years)	Sex (Females=1)	PDRLast	Outcome (Deceased=1)	Single/repeated admission	Diagnostic group
2013	2	6.58E+00	0	1.88E-01	0	Single admission	12
2013	0	6.57E+00	0	6.48E+00	0	Single admission	12
2013	0	6.55E+00	0	1.02E+01	0	Single admission	12
2013	1	6.55E+00	1	4.42E+00	0	Repeated admissions	12
2013	1	6.54E+00	1	7.51E-01	0	Single admission	12
2013	0	6.54E+00	1	8.87E-01	0	Single admission	12
2013	0	6.53E+00	0	3.05E+00	0	Repeated admissions	12
2013	0	6.53E+00	1	1.34E+00	0	Single admission	12
2013	0	6.52E+00	1	1.00E+00	0	Repeated admissions	12
2013	1	6.52E+00	0	2.44E+00	0	Single admission	12
2013	0	6.51E+00	0	4.47E+00	0	Single admission	12
2014	6	6.37E+00	1	1.22E+00	0	Single admission	1
2014	0	6.31E+00	0	1.16E+00	0	Repeated admissions	1
2014	0	1.95E-01	0	9.76E+00	1	Single admission	1
2014	0	6.22E+00	0	3.94E-01	0	Single admission	1
2014	7	5.95E+00	1	1.43E+00	0	Single admission	1
2014	1	5.78E+00	1	1.74E+00	0	Single admission	1
2014	9	5.67E+00	1	7.08E-01	0	Single admission	1
2014	0	5.66E+00	1	1.39E+01	0	Single admission	1
2014	1	5.64E+00	0	3.21E-01	0	Single admission	1
2014	0	5.62E+00	1	1.50E+00	0	Single admission	1
2014	16	5.53E+00	1	2.28E+00	0	Single admission	1
2014	0	6.25E+00	0	1.60E+00	0	Single admission	2
2014	0	2.11E+00	1	6.23E+00	1	Single admission	2
2014	0	6.18E+00	0	4.02E-01	0	Single admission	2
2014	0	6.14E+00	1	5.71E+00	0	Single admission	2
2014	0	6.12E+00	0	3.32E+01	0	Single admission	2
2014	0	6.12E+00	0	1.46E+00	0	Single admission	2
2014	0	6.08E+00	0	1.33E+00	0	Single admission	2
2014	0	6.07E+00	0	1.19E+00	0	Single admission	2

Admission year	Age (years)	Follow up time (years)	Sex (Females=1)	PDRLast	Outcome (Deceased=1)	Single/repeated admission	Diagnostic group
2014	0	6.07E+00	0	8.70E-01	0	Repeated admissions	2
2014	0	6.05E+00	0	6.07E+00	0	Single admission	2
2014	0	6.01E+00	1	2.00E+00	0	Repeated admissions	2
2014	0	5.99E+00	0	2.80E+00	0	Single admission	2
2014	0	5.96E+00	1	7.51E-01	0	Single admission	2
2014	0	5.96E+00	1	2.80E+00	0	Single admission	2
2014	0	5.93E+00	1	1.42E+00	0	Single admission	2
2014	0	5.90E+00	0	9.36E+00	0	Single admission	2
2014	0	5.69E+00	1	7.51E-01	0	Single admission	2
2014	0	5.64E+00	0	3.36E+00	0	Single admission	2
2014	0	5.64E+00	0	5.87E+00	0	Single admission	2
2014	0	6.43E+00	0	3.75E+00	0	Single admission	3
2014	6	6.39E+00	0	1.22E+00	0	Single admission	3
2014	4	6.36E+00	1	5.11E+00	0	Single admission	3
2014	13	6.36E+00	0	1.13E+00	0	Single admission	3
2014	12	6.21E+00	1	1.30E-01	0	Repeated admissions	3
2014	0	6.19E+00	1	7.22E+00	0	Single admission	3
2014	3	6.16E+00	0	1.45E+00	0	Single admission	3
2014	2	6.16E+00	1	1.20E+00	0	Single admission	3
2014	0	6.12E+00	1	1.07E-01	0	Single admission	3
2014	0	6.06E+00	0	5.37E+00	0	Single admission	3
2014	0	5.96E+00	0	5.04E+00	0	Single admission	3
2014	10	5.79E+00	0	8.87E-01	0	Single admission	3
2014	1	5.78E+00	0	8.26E-01	0	Repeated admissions	3
2014	0	5.74E+00	0	1.89E+00	0	Single admission	3
2014	1	1.11E-02	1	3.56E+01	1	Single admission	3
2014	0	5.67E+00	0	1.50E-01	0	Single admission	3
2014	0	6.46E+00	0	2.62E+00	0	Single admission	4
2014	1	6.42E+00	1	1.08E+01	0	Single admission	4
2014	1	6.40E+00	0	2.26E+00	0	Single admission	4

Admission year	Age (years)	Follow up time (years)	Sex (Females=1)	PDRLast	Outcome (Deceased=1)	Single/repeated admission	Diagnostic group
2014	0	6.39E+00	0	7.15E+00	0	Single admission	4
2014	1	6.38E+00	1	1.23E+00	0	Single admission	4
2014	2	6.31E+00	1	1.32E+00	0	Single admission	4
2014	18	6.31E+00	0	3.87E+00	0	Single admission	4
2014	0	6.30E+00	1	2.30E+01	0	Single admission	4
2014	0	6.23E+00	1	1.13E+00	0	Single admission	4
2014	6	6.22E+00	1	7.51E-01	0	Single admission	4
2014	0	6.21E+00	0	1.79E+00	0	Single admission	4
2014	1	6.13E+00	0	1.96E+00	0	Single admission	4
2014	0	6.13E+00	1	7.75E-01	0	Single admission	4
2014	0	6.10E+00	0	1.45E+00	0	Single admission	4
2014	0	6.06E+00	0	7.51E-01	0	Single admission	4
2014	0	5.89E+00	0	1.67E-01	0	Single admission	4
2014	5	5.82E+00	1	1.06E+00	0	Single admission	4
2014	0	5.80E+00	0	1.36E+00	0	Single admission	4
2014	1	5.78E+00	1	7.51E-01	0	Single admission	4
2014	0	5.67E+00	0	7.51E-01	0	Single admission	4
2014	12	5.65E+00	0	3.20E+00	0	Single admission	4
2014	0	5.59E+00	0	7.87E+00	0	Single admission	4
2014	0	5.50E+00	1	5.61E+00	0	Single admission	4
2014	6	5.49E+00	1	4.29E+00	0	Single admission	4
2014	0	5.48E+00	0	1.99E+00	0	Single admission	4
2014	4	6.39E+00	1	1.06E+00	0	Single admission	5
2014	8	6.38E+00	0	4.97E+00	0	Repeated admissions	5
2014	4	5.72E+00	0	1.50E+01	0	Single admission	5
2014	0	6.24E+00	0	9.48E+00	0	Single admission	5
2014	2	6.22E+00	0	1.56E-01	0	Repeated admissions	5
2014	4	6.17E+00	1	5.52E-01	0	Single admission	5
2014	10	6.16E+00	1	1.32E+00	0	Single admission	5
2014	1	3.17E+00	1	8.69E-01	1	Single admission	5

Admission year	Age (years)	Follow up time (years)	Sex (Females=1)	PDRLast	Outcome (Deceased=1)	Single/repeated admission	Diagnostic group
2014	1	9.66E-01	0	5.05E-01	1	Repeated admissions	5
2014	1	4.58E-01	0	3.06E+01	1	Repeated admissions	5
2014	2	6.04E+00	0	1.38E+00	0	Single admission	5
2014	7	6.02E+00	1	1.56E+00	0	Single admission	5
2014	3	5.99E+00	1	4.10E+00	0	Single admission	5
2014	15	1.94E+00	1	8.41E-01	1	Single admission	5
2014	2	5.90E+00	0	2.21E+00	0	Single admission	5
2014	2	5.86E+00	1	1.08E+00	0	Repeated admissions	5
2014	3	5.81E+00	0	1.52E-01	0	Repeated admissions	5
2014	9	5.81E+00	0	1.23E-01	0	Single admission	5
2014	11	5.80E+00	0	1.19E-01	0	Single admission	5
2014	5	1.87E-01	1	7.51E-01	1	Single admission	5
2014	5	5.77E+00	0	7.51E-01	0	Repeated admissions	5
2014	13	5.77E+00	0	1.54E-01	0	Single admission	5
2014	6	5.73E+00	1	1.54E-01	0	Single admission	5
2014	7	5.72E+00	0	9.24E-01	0	Single admission	5
2014	1	5.68E+00	0	1.80E+00	0	Single admission	5
2014	7	5.55E+00	1	1.93E-01	0	Single admission	5
2014	1	5.50E+00	1	1.20E+00	0	Single admission	5
2014	2	6.43E+00	1	1.52E+00	0	Repeated admissions	6
2014	3	1.28E+00	0	5.98E+00	1	Single admission	6
2014	3	6.37E+00	1	6.42E-01	0	Single admission	6
2014	0	6.35E+00	0	1.30E+00	0	Repeated admissions	6
2014	0	6.24E+00	1	2.05E+00	0	Single admission	6
2014	9	6.22E+00	0	1.24E+00	0	Single admission	6
2014	0	6.21E+00	0	2.10E+00	0	Single admission	6
2014	17	6.13E+00	0	4.33E+00	0	Single admission	6
2014	15	6.13E+00	0	6.62E+00	0	Single admission	6
2014	2	6.50E-04	0	8.63E+01	1	Single admission	6
2014	0	6.07E+00	0	1.11E+00	0	Single admission	6

Admission year	Age (years)	Follow up time (years)	Sex (Females=1)	PDRLast	Outcome (Deceased=1)	Single/repeated admission	Diagnostic group
2014	0	6.02E+00	0	9.89E-01	0	Single admission	6
2014	11	6.00E+00	1	6.61E+00	0	Single admission	6
2014	0	5.99E+00	0	2.71E-01	0	Repeated admissions	6
2014	11	5.98E+00	0	9.50E-01	0	Single admission	6
2014	1	5.92E+00	1	6.41E+00	0	Single admission	6
2014	15	5.90E+00	1	9.80E+01	0	Single admission	6
2014	0	5.90E+00	0	8.27E-01	0	Single admission	6
2014	2	5.81E+00	1	1.22E+00	0	Single admission	6
2014	16	5.81E+00	0	7.94E-01	0	Single admission	6
2014	0	5.80E+00	1	1.87E+00	0	Repeated admissions	6
2014	0	5.80E+00	0	1.23E+00	0	Single admission	6
2014	5	5.78E+00	0	1.83E+00	0	Single admission	6
2014	1	2.97E-01	0	5.31E+00	1	Repeated admissions	6
2014	0	5.69E+00	1	3.86E+00	0	Single admission	6
2014	0	5.69E+00	0	1.10E+00	0	Single admission	6
2014	0	5.66E+00	0	3.00E+00	0	Single admission	6
2014	0	5.65E+00	0	7.05E+00	0	Single admission	6
2014	3	5.64E+00	0	1.03E+00	0	Single admission	6
2014	0	5.62E+00	1	4.63E+01	0	Single admission	6
2014	0	5.60E+00	1	3.06E+01	0	Single admission	6
2014	1	1.02E+00	1	5.74E+00	1	Single admission	6
2014	16	5.54E+00	1	2.09E-01	0	Repeated admissions	6
2014	2	2.02E-02	1	9.97E+01	1	Single admission	6
2014	14	6.46E+00	0	2.63E+00	0	Single admission	7
2014	1	6.45E+00	0	6.43E+00	0	Single admission	7
2014	1	6.44E+00	0	5.32E+00	0	Single admission	7
2014	2	6.43E+00	1	1.18E+00	0	Single admission	7
2014	1	6.43E+00	1	1.14E+00	0	Single admission	7
2014	15	6.41E+00	0	1.68E+00	0	Single admission	7
2014	11	6.40E+00	1	2.63E-01	0	Single admission	7

Admission year	Age (years)	Follow up time (years)	Sex (Females=1)	PDRLast	Outcome (Deceased=1)	Single/repeated admission	Diagnostic group
2014	13	6.37E+00	0	3.69E-01	0	Single admission	7
2014	5	6.36E+00	0	2.16E+00	0	Single admission	7
2014	0	6.33E+00	1	9.16E+00	0	Single admission	7
2014	8	6.29E+00	1	3.18E+00	0	Single admission	7
2014	1	6.29E+00	1	5.50E+00	0	Single admission	7
2014	2	2.48E+00	0	9.44E+00	1	Single admission	7
2014	17	6.26E+00	0	3.18E+00	0	Single admission	7
2014	1	6.23E+00	1	2.09E+01	0	Repeated admissions	7
2014	17	6.21E+00	0	5.97E+00	0	Single admission	7
2014	1	6.21E+00	0	2.10E+00	0	Single admission	7
2014	0	6.20E+00	0	3.63E+00	0	Single admission	7
2014	13	6.17E+00	1	1.95E+00	0	Single admission	7
2014	12	6.13E+00	0	8.13E+00	0	Single admission	7
2014	11	6.13E+00	0	7.50E+00	0	Single admission	7
2014	11	6.10E+00	0	1.25E+00	0	Single admission	7
2014	9	1.74E+00	1	4.03E+00	1	Repeated admissions	7
2014	4	6.02E+00	1	2.01E+00	0	Single admission	7
2014	0	6.00E+00	1	7.51E-01	0	Single admission	7
2014	2	5.98E+00	0	2.84E+00	0	Repeated admissions	7
2014	14	5.93E+00	0	3.72E+00	0	Single admission	7
2014	10	5.93E+00	1	2.60E+00	0	Single admission	7
2014	6	2.33E-01	0	6.75E+00	1	Single admission	7
2014	1	5.91E+00	0	7.51E-01	0	Single admission	7
2014	5	5.88E+00	1	4.40E-01	0	Single admission	7
2014	1	5.87E+00	0	1.77E+00	0	Single admission	7
2014	11	5.86E+00	1	4.92E+00	0	Repeated admissions	7
2014	5	5.86E+00	1	4.29E+00	0	Single admission	7
2014	10	5.84E+00	1	1.31E+00	0	Single admission	7
2014	14	5.84E+00	0	2.31E+00	0	Single admission	7
2014	1	5.82E+00	0	9.08E-01	0	Single admission	7

Admission year	Age (years)	Follow up time (years)	Sex (Females=1)	PDRLast	Outcome (Deceased=1)	Single/repeated admission	Diagnostic group
2014	14	5.81E+00	1	9.08E-01	0	Single admission	7
2014	7	5.78E+00	0	1.07E+00	0	Single admission	7
2014	9	5.71E+00	1	1.12E+00	0	Single admission	7
2014	11	5.69E+00	1	4.88E-01	0	Single admission	7
2014	18	5.66E+00	1	4.60E-01	0	Single admission	7
2014	4	1.10E-02	1	5.12E+00	1	Repeated admissions	7
2014	10	5.65E+00	1	1.07E+00	0	Single admission	7
2014	2	5.63E+00	1	5.93E-01	0	Single admission	7
2014	0	5.62E+00	1	9.23E-01	0	Single admission	7
2014	18	5.61E+00	0	7.26E-01	0	Single admission	7
2014	13	5.61E+00	1	7.38E-01	0	Single admission	7
2014	0	5.54E+00	0	6.27E+00	0	Single admission	7
2014	16	5.53E+00	1	1.04E+00	0	Single admission	7
2014	9	5.52E+00	0	1.31E+00	0	Single admission	7
2014	14	5.52E+00	0	6.18E-01	0	Single admission	7
2014	10	5.50E+00	1	6.66E-01	0	Single admission	7
2014	12	6.44E+00	0	1.70E+00	0	Single admission	8
2014	4	6.44E+00	1	1.73E+00	0	Single admission	8
2014	7	6.43E+00	0	5.70E+00	0	Single admission	8
2014	12	6.42E+00	1	8.51E-01	0	Single admission	8
2014	10	6.41E+00	0	7.51E-01	0	Single admission	8
2014	15	6.40E+00	1	2.03E+00	0	Single admission	8
2014	3	6.36E+00	0	9.52E-01	0	Single admission	8
2014	15	6.36E+00	0	8.39E-01	0	Single admission	8
2014	9	6.32E+00	0	3.13E-01	0	Single admission	8
2014	12	6.32E+00	0	8.62E-01	0	Single admission	8
2014	4	5.32E+00	0	9.92E-01	0	Single admission	8
2014	2	6.29E+00	0	7.51E-01	0	Single admission	8
2014	1	6.27E+00	0	7.51E-01	0	Single admission	8
2014	10	6.26E+00	0	4.37E+00	0	Single admission	8

Admission year	Age (years)	Follow up time (years)	Sex (Females=1)	PDRLast	Outcome (Deceased=1)	Single/repeated admission	Diagnostic group
2014	13	6.25E+00	0	1.42E+00	0	Single admission	8
2014	15	6.24E+00	0	1.48E+00	0	Single admission	8
2014	10	6.22E+00	1	8.05E-01	0	Single admission	8
2014	5	6.21E+00	0	1.15E+00	0	Single admission	8
2014	13	6.18E+00	0	4.15E+00	0	Single admission	8
2014	9	6.17E+00	0	4.64E+00	0	Single admission	8
2014	3	6.16E+00	0	8.99E-01	0	Single admission	8
2014	15	6.15E+00	1	9.77E-01	0	Single admission	8
2014	14	6.12E+00	0	1.08E+00	0	Single admission	8
2014	3	6.10E+00	1	2.10E+00	0	Single admission	8
2014	4	6.10E+00	0	3.51E+00	0	Single admission	8
2014	15	6.08E+00	1	1.12E+00	0	Single admission	8
2014	2	6.08E+00	1	1.20E+00	0	Single admission	8
2014	0	6.07E+00	0	6.04E+00	0	Single admission	8
2014	13	6.07E+00	1	9.86E-01	0	Single admission	8
2014	14	6.04E+00	0	9.72E-01	0	Single admission	8
2014	11	6.01E+00	1	1.00E+00	0	Single admission	8
2014	16	6.01E+00	1	1.04E+00	0	Single admission	8
2014	0	6.00E+00	1	7.51E-01	0	Single admission	8
2014	1	5.99E+00	0	2.01E+00	0	Single admission	8
2014	12	5.99E+00	0	1.06E+00	0	Single admission	8
2014	6	3.10E-03	1	8.91E+01	1	Single admission	8
2014	11	5.97E+00	1	7.51E-01	0	Single admission	8
2014	6	5.97E+00	0	3.09E+00	0	Single admission	8
2014	2	5.96E+00	0	1.55E+01	0	Single admission	8
2014	7	5.92E+00	1	7.51E-01	0	Single admission	8
2014	17	5.92E+00	1	7.61E-01	0	Single admission	8
2014	12	5.91E+00	0	9.50E-01	0	Single admission	8
2014	7	5.90E+00	0	7.51E-01	0	Single admission	8
2014	0	5.88E+00	0	7.51E-01	0	Single admission	8

Admission year	Age (years)	Follow up time (years)	Sex (Females=1)	PDRLast	Outcome (Deceased=1)	Single/repeated admission	Diagnostic group
2014	10	5.86E+00	0	2.29E+00	0	Single admission	8
2014	1	5.86E+00	0	8.96E+00	0	Single admission	8
2014	14	5.86E+00	0	8.87E-01	0	Single admission	8
2014	17	5.86E+00	0	1.35E+00	0	Single admission	8
2014	2	5.86E+00	1	7.78E+00	0	Single admission	8
2014	6	5.85E+00	1	9.37E-01	0	Single admission	8
2014	3	5.84E+00	0	1.32E+00	0	Single admission	8
2014	14	5.81E+00	1	1.20E+00	0	Single admission	8
2014	17	5.75E+00	1	1.80E+00	0	Single admission	8
2014	11	5.74E+00	1	1.09E-01	0	Repeated admissions	8
2014	15	5.74E+00	0	1.11E+00	0	Single admission	8
2014	15	5.71E+00	0	2.01E+00	0	Single admission	8
2014	1	5.70E+00	0	7.51E-01	0	Single admission	8
2014	3	5.70E+00	0	2.24E+00	0	Single admission	8
2014	9	5.68E+00	0	7.51E-01	0	Single admission	8
2014	15	5.61E+00	1	3.58E-01	0	Single admission	8
2014	16	5.56E+00	1	1.28E+00	0	Single admission	8
2014	17	5.56E+00	1	1.22E+00	0	Single admission	8
2014	14	5.48E+00	1	4.24E+00	0	Single admission	8
2014	10	6.45E+00	1	1.07E-01	0	Single admission	9
2014	2	6.41E+00	0	4.41E-01	0	Single admission	9
2014	11	6.40E+00	1	1.93E-01	0	Single admission	9
2014	6	6.34E+00	1	8.30E+00	0	Single admission	9
2014	2	8.80E-01	0	3.11E-01	1	Single admission	9
2014	14	6.32E+00	1	4.40E-01	0	Single admission	9
2014	10	6.30E+00	1	3.61E-01	0	Single admission	9
2014	14	6.26E+00	1	2.04E+00	0	Single admission	9
2014	0	6.25E+00	0	8.88E-01	0	Single admission	9
2014	13	6.22E+00	1	1.18E+00	0	Single admission	9
2014	12	6.21E+00	1	1.97E+00	0	Repeated admissions	9

Admission year	Age (years)	Follow up time (years)	Sex (Females=1)	PDRLast	Outcome (Deceased=1)	Single/repeated admission	Diagnostic group
2014	13	6.20E+00	0	9.63E-01	0	Single admission	9
2014	10	6.19E+00	1	1.77E-01	0	Single admission	9
2014	9	6.74E-01	1	1.42E-01	1	Single admission	9
2014	7	7.35E-01	0	1.23E-01	1	Repeated admissions	9
2014	1	6.15E+00	0	1.39E+00	0	Single admission	9
2014	12	6.14E+00	1	1.83E+01	0	Single admission	9
2014	2	9.43E-01	0	1.56E-01	1	Repeated admissions	9
2014	4	6.11E+00	0	1.07E-01	0	Single admission	9
2014	14	6.10E+00	0	4.66E-01	0	Single admission	9
2014	12	6.09E+00	1	2.06E-01	0	Single admission	9
2014	0	6.09E+00	0	1.87E-01	0	Repeated admissions	9
2014	12	6.07E+00	1	1.42E-01	0	Single admission	9
2014	3	6.05E+00	1	8.59E-01	0	Single admission	9
2014	1	6.02E+00	0	3.16E-01	0	Single admission	9
2014	14	6.01E+00	1	2.71E-01	0	Single admission	9
2014	0	6.01E+00	0	5.21E-01	0	Single admission	9
2014	0	5.99E+00	0	3.89E-01	0	Single admission	9
2014	0	5.99E+00	0	5.06E-01	0	Single admission	9
2014	0	5.96E+00	1	2.12E-01	0	Single admission	9
2014	0	5.95E+00	1	2.71E-01	0	Single admission	9
2014	0	5.94E+00	0	7.51E-01	0	Single admission	9
2014	7	5.88E+00	0	1.46E-01	0	Single admission	9
2014	4	5.84E+00	0	4.49E-01	0	Single admission	9
2014	10	5.82E+00	0	1.72E+00	0	Single admission	9
2014	14	5.80E+00	0	7.69E-01	0	Single admission	9
2014	4	5.75E+00	0	5.30E-01	0	Single admission	9
2014	6	5.74E+00	0	1.23E+00	0	Single admission	9
2014	10	2.51E+00	0	1.77E+00	1	Repeated admissions	9
2014	3	5.73E+00	0	3.68E-01	0	Repeated admissions	9
2014	0	5.72E+00	1	6.21E-01	0	Repeated admissions	9

Admission year	Age (years)	Follow up time (years)	Sex (Females=1)	PDRLast	Outcome (Deceased=1)	Single/repeated admission	Diagnostic group
2014	5	5.69E+00	1	1.59E-01	0	Single admission	9
2014	0	5.69E+00	1	2.93E+00	0	Single admission	9
2014	3	5.69E+00	0	1.61E-01	0	Single admission	9
2014	0	5.67E+00	0	8.50E-01	0	Single admission	9
2014	14	5.63E+00	0	2.71E-01	0	Single admission	9
2014	4	5.62E+00	1	1.70E-01	0	Single admission	9
2014	14	5.62E+00	1	1.54E-01	0	Single admission	9
2014	3	5.61E+00	0	2.71E-01	0	Single admission	9
2014	6	5.59E+00	0	1.07E-01	0	Single admission	9
2014	3	5.59E+00	0	1.65E-01	0	Single admission	9
2014	2	5.59E+00	0	7.51E-01	0	Repeated admissions	9
2014	0	5.57E+00	0	1.07E-01	0	Single admission	9
2014	0	5.55E+00	1	2.12E+00	0	Single admission	9
2014	1	4.64E+00	0	1.72E-01	0	Single admission	9
2014	7	5.53E+00	1	1.46E-01	0	Single admission	9
2014	0	5.50E+00	0	3.68E-01	0	Single admission	9
2014	3	6.47E+00	1	1.65E+00	0	Single admission	10
2014	0	2.35E+00	0	1.40E+00	1	Single admission	10
2014	15	6.37E+00	0	9.89E-01	0	Repeated admissions	10
2014	10	5.80E+00	1	7.80E+00	1	Repeated admissions	10
2014	0	6.36E+00	0	4.52E+00	0	Single admission	10
2014	3	6.32E+00	1	5.79E+00	0	Single admission	10
2014	2	6.30E+00	0	1.08E+00	0	Repeated admissions	10
2014	2	6.28E+00	0	2.86E-01	0	Single admission	10
2014	0	2.60E-01	1	1.33E+00	1	Repeated admissions	10
2014	2	6.25E+00	0	1.77E+00	0	Single admission	10
2014	16	6.25E+00	0	1.89E-01	0	Single admission	10
2014	0	6.24E+00	0	2.31E-01	0	Repeated admissions	10
2014	10	6.23E+00	0	4.11E+00	0	Single admission	10
2014	11	6.22E+00	0	3.44E+00	0	Single admission	10

Admission year	Age (years)	Follow up time (years)	Sex (Females=1)	PDRLast	Outcome (Deceased=1)	Single/repeated admission	Diagnostic group
2014	0	6.21E+00	0	7.51E-01	0	Single admission	10
2014	1	6.12E+00	0	7.51E-01	0	Single admission	10
2014	5	6.11E+00	1	1.05E+00	0	Single admission	10
2014	0	6.10E+00	1	1.30E+00	0	Single admission	10
2014	3	6.09E+00	1	1.02E+00	0	Single admission	10
2014	1	6.05E+00	0	1.15E-01	0	Repeated admissions	10
2014	1	6.03E+00	1	6.33E+00	0	Single admission	10
2014	0	6.00E+00	1	7.51E-01	0	Single admission	10
2014	1	5.90E+00	0	1.17E+00	0	Repeated admissions	10
2014	0	5.89E+00	0	7.67E+00	0	Single admission	10
2014	0	5.88E+00	0	2.15E+00	0	Single admission	10
2014	1	5.87E+00	0	2.25E+00	0	Single admission	10
2014	2	5.83E+00	0	9.63E-01	0	Single admission	10
2014	13	5.82E+00	0	3.82E+00	0	Single admission	10
2014	2	5.80E+00	1	4.59E+00	0	Repeated admissions	10
2014	2	5.78E+00	1	4.13E+00	0	Repeated admissions	10
2014	1	1.04E+00	0	7.24E+00	1	Repeated admissions	10
2014	2	5.72E+00	0	1.31E+00	0	Repeated admissions	10
2014	1	4.58E-01	1	9.24E-01	1	Single admission	10
2014	2	5.70E+00	0	9.90E-01	0	Single admission	10
2014	3	5.69E+00	0	1.02E+00	0	Single admission	10
2014	1	5.69E+00	1	5.68E+00	0	Single admission	10
2014	3	5.67E+00	0	1.00E+00	0	Repeated admissions	10
2014	6	5.67E+00	1	4.04E+00	0	Repeated admissions	10
2014	1	5.67E+00	0	7.51E-01	0	Single admission	10
2014	7	5.66E+00	0	1.02E+00	0	Single admission	10
2014	3	5.65E+00	0	3.66E+00	0	Repeated admissions	10
2014	6	5.56E+00	1	9.24E-01	0	Repeated admissions	10
2014	2	7.07E-01	0	4.68E+00	0	Single admission	10
2014	4	5.54E+00	0	1.18E+00	0	Single admission	10

Admission year	Age (years)	Follow up time (years)	Sex (Females=1)	PDRLast	Outcome (Deceased=1)	Single/repeated admission	Diagnostic group
2014	16	5.52E+00	0	9.77E-01	0	Repeated admissions	10
2014	3	5.52E+00	1	9.87E-01	0	Single admission	10
2014	5	5.50E+00	0	7.51E-01	0	Repeated admissions	10
2014	8	5.50E+00	1	1.29E+00	0	Repeated admissions	10
2014	0	5.48E+00	1	1.58E+00	0	Single admission	10
2014	0	6.46E+00	0	3.14E+01	0	Single admission	11
2014	0	6.46E+00	0	1.58E+00	0	Single admission	11
2014	0	6.45E+00	0	2.05E+00	0	Single admission	11
2014	0	6.44E+00	1	3.87E+00	0	Single admission	11
2014	0	6.43E+00	0	7.51E-01	0	Single admission	11
2014	0	6.43E+00	0	3.92E+00	0	Repeated admissions	11
2014	12	6.41E+00	1	8.36E-01	0	Single admission	11
2014	3	6.41E+00	0	8.95E-01	0	Repeated admissions	11
2014	0	6.40E+00	0	1.42E+00	0	Single admission	11
2014	0	6.38E+00	1	1.24E+01	0	Single admission	11
2014	0	6.37E+00	0	1.30E+01	0	Single admission	11
2014	5	6.37E+00	1	1.25E-01	0	Single admission	11
2014	0	6.35E+00	1	2.73E+00	0	Single admission	11
2014	0	6.34E+00	0	1.42E+01	0	Single admission	11
2014	2	1.63E+00	1	2.86E+00	1	Repeated admissions	11
2014	0	6.30E+00	1	6.95E+00	0	Single admission	11
2014	2	6.27E+00	1	3.27E-01	0	Repeated admissions	11
2014	0	2.72E-01	1	6.91E+00	1	Repeated admissions	11
2014	0	6.25E+00	1	1.67E+00	0	Single admission	11
2014	0	6.23E+00	0	2.13E+00	0	Single admission	11
2014	0	6.23E+00	0	1.45E+00	0	Single admission	11
2014	0	6.21E+00	0	2.20E+00	0	Single admission	11
2014	0	6.18E+00	1	2.96E+00	0	Single admission	11
2014	0	6.17E+00	1	2.56E+00	0	Single admission	11
2014	0	6.15E+00	1	5.93E-01	0	Repeated admissions	11

Admission year	Age (years)	Follow up time (years)	Sex (Females=1)	PDRLast	Outcome (Deceased=1)	Single/repeated admission	Diagnostic group
2014	10	6.15E+00	1	1.58E-01	0	Single admission	11
2014	0	6.14E+00	1	1.32E+00	0	Single admission	11
2014	0	6.12E+00	0	2.98E-01	0	Single admission	11
2014	0	6.10E+00	0	7.51E-01	0	Single admission	11
2014	0	5.84E+00	0	2.11E+00	0	Single admission	11
2014	0	6.07E+00	0	8.18E+00	0	Single admission	11
2014	0	6.03E+00	0	1.44E+00	0	Single admission	11
2014	0	6.00E+00	0	8.85E+00	0	Single admission	11
2014	0	6.00E+00	1	3.91E+00	0	Repeated admissions	11
2014	2	5.99E+00	0	2.73E-01	0	Repeated admissions	11
2014	0	5.98E+00	1	7.51E-01	0	Single admission	11
2014	0	5.97E+00	1	7.60E+00	0	Single admission	11
2014	0	5.94E+00	0	1.14E+00	0	Repeated admissions	11
2014	1	5.92E+00	1	2.29E+00	0	Repeated admissions	11
2014	0	5.90E+00	0	2.98E-01	0	Single admission	11
2014	0	5.89E+00	0	1.80E+01	0	Single admission	11
2014	0	5.88E+00	0	5.34E+00	0	Single admission	11
2014	0	5.87E+00	0	1.64E+00	0	Single admission	11
2014	0	5.86E+00	1	9.77E-01	0	Single admission	11
2014	0	5.84E+00	0	9.32E-01	0	Single admission	11
2014	0	5.84E+00	0	1.67E+00	0	Repeated admissions	11
2014	0	5.84E+00	0	2.07E+00	0	Single admission	11
2014	0	5.82E+00	0	8.70E+00	0	Single admission	11
2014	0	5.79E+00	1	8.96E+00	0	Repeated admissions	11
2014	0	5.75E+00	1	1.59E+00	0	Single admission	11
2014	0	5.69E+00	0	1.22E+00	0	Single admission	11
2014	0	5.66E+00	0	5.03E+00	0	Single admission	11
2014	0	5.63E+00	0	5.53E+00	0	Repeated admissions	11
2014	0	5.62E+00	1	7.51E-01	0	Single admission	11
2014	1	1.61E+00	1	2.51E+00	1	Repeated admissions	11

Admission year	Age (years)	Follow up time (years)	Sex (Females=1)	PDRLast	Outcome (Deceased=1)	Single/repeated admission	Diagnostic group
2014	0	5.61E+00	0	3.30E+00	0	Single admission	11
2014	0	5.60E+00	0	3.92E+00	0	Single admission	11
2014	8	5.58E+00	0	8.39E-01	0	Single admission	11
2014	0	5.57E+00	0	7.12E-01	0	Single admission	11
2014	1	4.78E-01	1	9.59E+01	1	Repeated admissions	11
2014	0	5.57E+00	0	7.51E-01	0	Repeated admissions	11
2014	0	5.56E+00	1	7.09E+00	0	Single admission	11
2014	0	5.55E+00	0	1.64E+00	0	Single admission	11
2014	0	5.54E+00	0	9.90E-01	0	Repeated admissions	11
2014	0	5.53E+00	0	4.35E+00	0	Single admission	11
2014	1	5.09E+00	0	6.67E+00	0	Repeated admissions	11
2014	0	5.52E+00	0	3.89E+00	0	Single admission	11
2014	0	5.49E+00	0	7.51E-01	0	Repeated admissions	11
2014	18	6.45E+00	1	7.94E-01	0	Single admission	12
2014	1	6.45E+00	1	1.78E+00	0	Single admission	12
2014	0	6.45E+00	0	3.23E-01	0	Single admission	12
2014	0	6.44E+00	1	7.37E-01	0	Repeated admissions	12
2014	0	6.44E+00	1	6.90E-01	0	Single admission	12
2014	1	1.94E+00	1	2.46E+00	0	Repeated admissions	12
2014	0	1.92E-01	0	1.84E+00	1	Repeated admissions	12
2014	2	6.42E+00	0	3.18E-01	0	Repeated admissions	12
2014	0	6.42E+00	0	1.72E+00	0	Single admission	12
2014	0	6.40E+00	0	1.48E+00	0	Single admission	12
2014	0	6.39E+00	0	2.12E+01	0	Single admission	12
2014	1	6.38E+00	1	7.51E-01	0	Single admission	12
2014	0	6.38E+00	0	7.51E-01	0	Single admission	12
2014	0	6.36E+00	0	9.32E-01	0	Single admission	12
2014	8	6.35E+00	1	1.21E+01	0	Single admission	12
2014	2	6.34E+00	0	2.86E-01	0	Single admission	12
2014	1	6.34E+00	0	1.00E+00	0	Single admission	12

Admission year	Age (years)	Follow up time (years)	Sex (Females=1)	PDRLast	Outcome (Deceased=1)	Single/repeated admission	Diagnostic group
2014	7	6.34E+00	1	1.30E+00	0	Single admission	12
2014	0	6.34E+00	0	1.56E-01	0	Single admission	12
2014	0	6.32E+00	1	1.10E+00	0	Single admission	12
2014	0	6.31E+00	1	8.82E-01	0	Single admission	12
2014	0	6.30E+00	0	7.26E-01	0	Single admission	12
2014	0	6.30E+00	1	1.08E+00	0	Single admission	12
2014	11	6.29E+00	1	9.62E-01	0	Single admission	12
2014	0	6.28E+00	0	2.75E-01	0	Single admission	12
2014	0	6.28E+00	1	1.35E+00	0	Single admission	12
2014	11	6.28E+00	0	7.51E-01	0	Single admission	12
2014	3	6.26E+00	1	4.29E-01	0	Single admission	12
2014	1	6.26E+00	0	1.23E+00	0	Single admission	12
2014	0	6.25E+00	1	2.79E+00	0	Single admission	12
2014	0	6.25E+00	1	6.85E-01	0	Single admission	12
2014	0	6.25E+00	0	1.56E-01	0	Single admission	12
2014	1	4.96E+00	1	3.85E-01	0	Single admission	12
2014	1	6.24E+00	0	7.90E-01	0	Repeated admissions	12
2014	0	6.23E+00	0	1.18E+00	0	Single admission	12
2014	2	6.22E+00	1	2.03E-01	0	Single admission	12
2014	0	6.22E+00	0	7.51E-01	0	Single admission	12
2014	0	6.22E+00	0	1.56E-01	0	Single admission	12
2014	0	6.20E+00	0	6.21E+00	0	Repeated admissions	12
2014	0	6.20E+00	1	2.91E-01	0	Single admission	12
2014	2	6.19E+00	1	1.56E-01	0	Single admission	12
2014	0	6.19E+00	1	1.56E-01	0	Single admission	12
2014	0	6.19E+00	1	1.56E-01	0	Single admission	12
2014	0	6.17E+00	0	1.47E+00	0	Single admission	12
2014	0	6.17E+00	1	7.51E-01	0	Single admission	12
2014	6	6.16E+00	0	2.81E-01	0	Single admission	12
2014	14	6.16E+00	0	1.99E-01	0	Single admission	12

Admission year	Age (years)	Follow up time (years)	Sex (Females=1)	PDRLast	Outcome (Deceased=1)	Single/repeated admission	Diagnostic group
2014	1	6.15E+00	1	1.52E+00	0	Single admission	12
2014	0	6.09E+00	1	2.55E-01	0	Single admission	12
2014	0	6.09E+00	0	1.90E+00	0	Single admission	12
2014	1	6.09E+00	0	3.12E-01	0	Single admission	12
2014	0	6.09E+00	0	2.80E+00	0	Single admission	12
2014	0	6.08E+00	0	2.65E+00	0	Single admission	12
2014	1	6.08E+00	0	4.85E+00	0	Repeated admissions	12
2014	0	6.08E+00	0	7.51E-01	0	Repeated admissions	12
2014	0	6.07E+00	1	1.47E+00	0	Single admission	12
2014	5	6.05E+00	1	1.65E+01	0	Single admission	12
2014	0	6.05E+00	1	1.56E-01	0	Single admission	12
2014	0	6.04E+00	0	7.39E+00	0	Single admission	12
2014	12	6.04E+00	1	1.10E+01	0	Single admission	12
2014	2	6.01E+00	1	1.54E+00	0	Single admission	12
2014	0	5.99E+00	0	2.86E-01	0	Repeated admissions	12
2014	13	5.99E+00	1	3.39E+00	0	Single admission	12
2014	0	5.96E+00	0	2.43E+00	0	Single admission	12
2014	0	5.96E+00	1	1.92E-01	0	Repeated admissions	12
2014	0	5.95E+00	0	1.18E+00	0	Single admission	12
2014	0	5.95E+00	0	3.48E-01	0	Single admission	12
2014	1	5.92E+00	0	2.98E-01	0	Single admission	12
2014	1	5.87E+00	0	6.78E+00	0	Single admission	12
2014	1	5.86E+00	0	1.64E-01	0	Single admission	12
2014	0	5.82E+00	0	6.65E+00	0	Single admission	12
2014	0	5.82E+00	0	2.70E+00	0	Repeated admissions	12
2014	1	5.81E+00	0	3.72E+00	0	Single admission	12
2014	1	5.80E+00	1	9.46E-01	0	Single admission	12
2014	0	5.80E+00	0	2.06E+00	0	Single admission	12
2014	10	5.80E+00	1	2.82E+00	0	Repeated admissions	12
2014	0	5.73E+00	0	2.80E+00	0	Single admission	12

Admission year	Age (years)	Follow up time (years)	Sex (Females=1)	PDRLast	Outcome (Deceased=1)	Single/repeated admission	Diagnostic group
2014	0	5.70E+00	1	1.99E+00	0	Single admission	12
2014	0	5.68E+00	1	3.78E+00	0	Single admission	12
2014	1	9.14E-03	0	8.89E+00	1	Single admission	12
2014	0	5.66E+00	1	2.98E-01	0	Single admission	12
2014	9	3.29E+00	1	6.53E-01	1	Repeated admissions	12
2014	0	5.65E+00	1	5.11E+00	0	Single admission	12
2014	0	5.65E+00	0	9.99E-01	0	Single admission	12
2014	0	5.63E+00	1	2.56E+00	0	Repeated admissions	12
2014	9	5.61E+00	1	5.38E-01	0	Single admission	12
2014	1	5.61E+00	1	5.65E+00	0	Repeated admissions	12
2014	0	5.61E+00	0	3.84E+00	0	Repeated admissions	12
2014	3	5.60E+00	0	7.51E-01	0	Single admission	12
2014	1	5.58E+00	1	2.91E-01	0	Single admission	12
2014	16	5.58E+00	0	3.93E+00	0	Single admission	12
2014	2	5.57E+00	0	1.44E+00	0	Repeated admissions	12
2014	0	5.56E+00	1	9.65E-01	0	Single admission	12
2014	0	5.56E+00	0	1.13E+00	0	Single admission	12
2014	0	5.54E+00	0	9.99E-01	0	Single admission	12
2014	0	5.53E+00	0	3.62E-01	0	Single admission	12
2014	0	5.53E+00	0	5.81E-01	0	Single admission	12
2014	2	5.53E+00	1	1.03E+00	0	Single admission	12
2014	5	1.28E+00	1	6.30E+01	1	Repeated admissions	12
2014	0	5.52E+00	0	1.60E+00	0	Single admission	12
2014	0	5.51E+00	0	2.23E+00	0	Single admission	12
2014	0	5.51E+00	0	1.22E+00	0	Single admission	12
2014	0	5.51E+00	1	1.28E+00	0	Single admission	12
2014	1	5.51E+00	0	5.91E-01	0	Single admission	12
2014	0	5.50E+00	0	7.51E-01	0	Single admission	12
2014	2	5.49E+00	1	6.69E+00	0	Single admission	12
2014	3	5.49E+00	1	2.07E-01	0	Single admission	12

Admission year	Age (years)	Follow up time (years)	Sex (Females=1)	PDRLast	Outcome (Deceased=1)	Single/repeated admission	Diagnostic group
2014	0	5.49E+00	1	8.93E-01	0	Single admission	12
2014	0	5.49E+00	0	2.72E-01	0	Single admission	12
2014	0	5.48E+00	1	1.67E-01	0	Repeated admissions	12
2014	0	5.48E+00	1	2.88E-01	0	Single admission	12
2014	0	1.12E-01	0	3.61E+00	1	Single admission	12
2014	0	6.32E+00	1	7.51E-01	0	Single admission	13
2014	0	5.98E+00	0	1.58E+00	0	Repeated admissions	13
2014	0	5.60E+00	0	1.72E+00	0	Single admission	13
2015	11	5.34E+00	0	1.14E+00	0	Single admission	1
2015	0	8.56E-04	0	3.24E+01	1	Single admission	1
2015	0	5.17E+00	0	1.71E+00	0	Single admission	1
2015	5	5.06E+00	1	1.22E+00	0	Single admission	1
2015	0	7.67E-02	0	1.00E+01	1	Single admission	1
2015	0	1.85E-01	1	9.90E-01	1	Repeated admissions	1
2015	1	4.70E+00	1	8.91E-01	0	Repeated admissions	1
2015	4	4.59E+00	0	1.77E+00	0	Single admission	1
2015	0	5.35E+00	0	1.84E+00	0	Single admission	2
2015	0	5.34E+00	0	1.11E+01	0	Single admission	2
2015	0	5.33E+00	0	7.51E-01	0	Single admission	2
2015	0	5.33E+00	1	7.51E-01	0	Single admission	2
2015	0	5.33E+00	0	1.65E+00	0	Single admission	2
2015	0	5.32E+00	1	7.42E+00	0	Single admission	2
2015	1	5.31E+00	0	7.14E+00	0	Repeated admissions	2
2015	0	5.29E+00	0	7.27E+00	0	Single admission	2
2015	0	5.19E+00	0	7.51E-01	0	Single admission	2
2015	0	5.12E+00	1	1.11E+00	0	Single admission	2
2015	1	5.10E+00	0	3.25E-01	0	Repeated admissions	2
2015	0	5.09E+00	1	1.35E+00	0	Single admission	2
2015	0	5.05E+00	0	1.02E+00	0	Single admission	2
2015	0	4.86E+00	0	1.30E+00	0	Single admission	2

Admission year	Age (years)	Follow up time (years)	Sex (Females=1)	PDRLast	Outcome (Deceased=1)	Single/repeated admission	Diagnostic group
2015	0	4.86E+00	0	9.51E+00	0	Single admission	2
2015	0	4.80E+00	0	2.59E+00	0	Single admission	2
2015	0	4.80E+00	1	1.86E+00	0	Single admission	2
2015	0	4.80E+00	1	8.46E+00	0	Single admission	2
2015	1	5.20E-01	0	1.68E+01	1	Repeated admissions	2
2015	0	4.76E+00	1	1.16E+01	0	Single admission	2
2015	0	4.74E+00	1	7.51E-01	0	Single admission	2
2015	0	4.61E+00	0	2.52E+00	0	Single admission	2
2015	0	4.61E+00	0	2.11E+00	0	Single admission	2
2015	0	4.60E+00	0	2.29E+00	0	Single admission	2
2015	0	4.60E+00	0	1.02E+00	0	Single admission	2
2015	0	4.59E+00	1	1.02E+00	0	Single admission	2
2015	0	4.58E+00	0	5.26E+00	0	Single admission	2
2015	0	4.57E+00	0	1.56E-01	0	Single admission	2
2015	0	4.97E-03	1	6.25E+01	1	Single admission	2
2015	0	4.51E+00	0	1.30E+00	0	Single admission	2
2015	0	4.49E+00	0	4.04E+00	0	Single admission	2
2015	0	5.43E+00	1	2.09E+00	0	Single admission	3
2015	0	5.41E+00	0	1.06E+00	0	Single admission	3
2015	12	5.28E+00	0	7.51E-01	0	Repeated admissions	3
2015	9	5.23E+00	0	5.88E+00	0	Single admission	3
2015	0	5.15E+00	0	3.85E-01	0	Single admission	3
2015	16	5.08E+00	0	1.49E+00	0	Single admission	3
2015	10	8.58E-01	0	8.64E-01	1	Single admission	3
2015	13	4.91E+00	0	2.71E-01	0	Single admission	3
2015	0	4.90E+00	0	1.13E+01	0	Single admission	3
2015	0	4.86E+00	0	2.71E+00	0	Single admission	3
2015	0	4.84E+00	0	1.02E+00	0	Single admission	3
2015	0	4.76E+00	1	1.11E+00	0	Single admission	3
2015	0	4.72E+00	1	1.11E+00	0	Single admission	3

Admission year	Age (years)	Follow up time (years)	Sex (Females=1)	PDRLast	Outcome (Deceased=1)	Single/repeated admission	Diagnostic group
2015	0	1.65E+00	0	3.28E+00	1	Repeated admissions	3
2015	13	4.58E+00	0	1.26E+00	0	Repeated admissions	3
2015	2	4.58E+00	1	8.09E+00	0	Single admission	3
2015	0	4.52E+00	1	4.60E+00	0	Single admission	3
2015	0	4.51E+00	0	3.53E-01	0	Single admission	3
2015	0	5.47E+00	0	1.07E+01	0	Single admission	4
2015	1	6.51E-01	0	2.57E+00	1	Repeated admissions	4
2015	1	2.93E-01	1	1.05E+00	1	Single admission	4
2015	0	5.30E+00	0	7.51E-01	0	Single admission	4
2015	0	5.30E+00	1	9.53E+00	0	Single admission	4
2015	1	5.25E+00	1	9.90E-01	0	Repeated admissions	4
2015	14	1.06E+00	0	1.65E+01	1	Single admission	4
2015	4	5.22E+00	0	6.66E+00	0	Single admission	4
2015	2	5.18E+00	1	4.92E+00	0	Repeated admissions	4
2015	0	5.10E+00	1	7.94E-01	0	Repeated admissions	4
2015	0	5.08E+00	0	1.90E+00	0	Single admission	4
2015	11	5.07E+00	0	2.05E+01	0	Single admission	4
2015	0	5.04E+00	1	2.80E+00	0	Single admission	4
2015	1	5.03E+00	0	7.51E-01	0	Single admission	4
2015	9	5.03E+00	1	1.08E+00	0	Single admission	4
2015	1	5.02E+00	0	8.99E-01	0	Single admission	4
2015	0	5.00E+00	1	2.62E+00	0	Single admission	4
2015	14	4.97E+00	0	2.07E+00	0	Single admission	4
2015	0	4.41E+00	0	1.88E+00	0	Single admission	4
2015	15	4.85E+00	1	1.54E+00	0	Single admission	4
2015	0	4.73E+00	0	1.76E+00	0	Repeated admissions	4
2015	1	4.67E+00	0	2.29E+00	0	Single admission	4
2015	1	4.67E+00	1	2.23E+00	0	Single admission	4
2015	16	4.53E+00	1	1.22E+00	0	Single admission	4
2015	11	4.51E+00	0	9.11E-01	0	Single admission	4

Admission year	Age (years)	Follow up time (years)	Sex (Females=1)	PDRLast	Outcome (Deceased=1)	Single/repeated admission	Diagnostic group
2015	2	4.51E+00	1	4.39E+00	0	Single admission	4
2015	18	4.50E+00	1	2.38E+00	0	Repeated admissions	4
2015	3	5.47E+00	0	1.07E+00	0	Single admission	5
2015	3	5.42E+00	0	2.47E-01	0	Single admission	5
2015	13	5.35E+00	1	1.60E+00	0	Single admission	5
2015	0	5.33E+00	1	4.25E+00	0	Single admission	5
2015	13	5.33E+00	0	1.15E-01	0	Repeated admissions	5
2015	3	8.93E-01	0	2.34E-01	1	Single admission	5
2015	2	5.31E+00	0	9.50E-01	0	Single admission	5
2015	0	5.30E+00	0	1.13E+00	0	Single admission	5
2015	1	5.23E+00	1	1.07E-01	0	Single admission	5
2015	7	5.23E+00	1	1.18E+00	0	Single admission	5
2015	18	1.88E-03	0	5.04E+00	1	Single admission	5
2015	2	5.19E+00	0	8.27E-01	0	Single admission	5
2015	3	5.19E+00	1	1.75E-01	0	Single admission	5
2015	17	1.38E-02	0	5.23E+01	1	Single admission	5
2015	1	5.08E+00	1	3.95E-01	0	Single admission	5
2015	7	5.08E+00	0	1.07E-01	0	Single admission	5
2015	5	2.09E+00	1	1.57E-01	1	Single admission	5
2015	1	5.05E+00	0	3.45E+00	0	Repeated admissions	5
2015	2	5.02E+00	0	2.16E-01	0	Single admission	5
2015	13	4.97E+00	1	1.09E+01	0	Repeated admissions	5
2015	0	4.93E+00	0	4.11E-01	0	Repeated admissions	5
2015	1	4.90E+00	0	8.12E-01	0	Single admission	5
2015	5	4.90E+00	1	1.27E+00	0	Single admission	5
2015	2	4.90E+00	0	3.40E-01	0	Single admission	5
2015	3	1.90E+00	1	1.17E+00	1	Repeated admissions	5
2015	6	4.88E+00	0	2.67E-01	0	Repeated admissions	5
2015	2	4.86E+00	1	1.15E+00	0	Single admission	5
2015	11	4.84E+00	1	1.31E-01	0	Single admission	5

Admission year	Age (years)	Follow up time (years)	Sex (Females=1)	PDRLast	Outcome (Deceased=1)	Single/repeated admission	Diagnostic group
2015	7	4.82E+00	1	5.62E-01	0	Repeated admissions	5
2015	8	4.78E+00	1	2.39E-01	0	Single admission	5
2015	1	4.77E+00	0	1.07E-01	0	Single admission	5
2015	0	4.71E+00	0	9.91E-01	0	Single admission	5
2015	16	2.93E+00	1	2.20E-01	1	Single admission	5
2015	1	4.67E+00	1	5.82E-01	0	Single admission	5
2015	5	4.65E+00	1	8.99E-01	0	Single admission	5
2015	0	3.08E-01	0	9.33E-01	1	Repeated admissions	5
2015	13	4.62E+00	0	5.66E-01	0	Single admission	5
2015	11	4.60E+00	0	3.48E-01	0	Single admission	5
2015	5	4.59E+00	1	1.61E-01	0	Single admission	5
2015	0	4.58E+00	0	6.98E+00	0	Single admission	5
2015	0	4.48E+00	0	1.38E+00	0	Single admission	5
2015	0	5.44E+00	0	9.37E+00	0	Single admission	6
2015	1	5.43E+00	0	2.41E+01	0	Single admission	6
2015	1	5.43E+00	0	7.51E-01	0	Single admission	6
2015	11	5.42E+00	0	2.56E+01	0	Single admission	6
2015	1	5.35E+00	0	1.09E+00	0	Single admission	6
2015	0	5.34E+00	0	8.16E-01	0	Single admission	6
2015	15	8.01E-01	0	3.91E+00	1	Repeated admissions	6
2015	11	5.28E+00	1	7.27E+00	0	Single admission	6
2015	0	5.24E+00	1	2.71E-01	0	Repeated admissions	6
2015	1	5.19E+00	1	5.13E+00	0	Single admission	6
2015	8	5.13E+00	1	1.97E+00	0	Single admission	6
2015	10	5.12E+00	1	7.83E-01	0	Single admission	6
2015	0	5.07E+00	0	1.54E+00	0	Single admission	6
2015	13	7.99E-03	0	1.56E+01	1	Single admission	6
2015	0	5.02E+00	1	5.18E+00	0	Single admission	6
2015	1	5.02E+00	0	1.50E+00	0	Repeated admissions	6
2015	15	5.01E+00	0	8.83E+00	0	Single admission	6

Admission year	Age (years)	Follow up time (years)	Sex (Females=1)	PDRLast	Outcome (Deceased=1)	Single/repeated admission	Diagnostic group
2015	15	5.00E+00	0	2.31E+01	0	Single admission	6
2015	2	4.19E+00	0	7.51E-01	1	Repeated admissions	6
2015	0	8.38E-02	1	7.25E+01	1	Single admission	6
2015	8	4.94E+00	0	4.20E+00	0	Single admission	6
2015	0	4.92E+00	0	9.70E-01	0	Single admission	6
2015	1	4.87E+00	0	1.03E+01	0	Repeated admissions	6
2015	0	4.81E+00	0	1.36E+01	0	Single admission	6
2015	0	4.79E+00	0	7.51E-01	0	Single admission	6
2015	1	7.71E-01	1	2.95E+01	1	Repeated admissions	6
2015	4	4.74E+00	0	1.12E+00	0	Single admission	6
2015	1	4.73E+00	0	2.11E+00	0	Repeated admissions	6
2015	1	4.66E+00	1	3.09E+00	0	Single admission	6
2015	10	4.66E+00	0	3.44E-01	0	Single admission	6
2015	0	4.65E+00	0	2.06E+00	0	Single admission	6
2015	5	4.62E+00	0	2.26E+00	0	Repeated admissions	6
2015	8	4.61E+00	1	9.63E-01	0	Single admission	6
2015	0	4.60E+00	1	2.00E+00	0	Single admission	6
2015	7	4.58E+00	1	2.58E+00	0	Repeated admissions	6
2015	3	6.47E-03	0	6.68E+01	1	Single admission	6
2015	0	4.56E+00	0	1.24E+00	0	Single admission	6
2015	0	1.14E-01	0	2.26E+01	1	Single admission	6
2015	0	4.49E+00	0	5.92E+00	0	Single admission	6
2015	9	5.47E+00	1	6.38E-01	0	Single admission	7
2015	0	1.49E-01	0	9.93E+01	1	Repeated admissions	7
2015	5	5.45E+00	0	1.14E+00	0	Single admission	7
2015	15	5.45E+00	1	5.61E-01	0	Single admission	7
2015	13	5.44E+00	0	1.68E+00	0	Single admission	7
2015	10	5.43E+00	1	8.19E-01	0	Single admission	7
2015	1	5.33E+00	1	2.08E+00	0	Single admission	7
2015	0	5.33E+00	0	5.67E+00	0	Single admission	7

Admission year	Age (years)	Follow up time (years)	Sex (Females=1)	PDRLast	Outcome (Deceased=1)	Single/repeated admission	Diagnostic group
2015	6	5.29E+00	1	2.74E+00	0	Single admission	7
2015	3	5.27E+00	1	1.00E+00	0	Single admission	7
2015	0	5.23E+00	0	1.40E+00	0	Single admission	7
2015	12	5.23E+00	0	8.53E-01	0	Single admission	7
2015	13	5.22E+00	0	1.47E+00	0	Single admission	7
2015	14	5.19E+00	0	1.26E+00	0	Single admission	7
2015	10	1.48E-01	1	1.15E+00	0	Single admission	7
2015	18	5.15E+00	0	6.99E+00	0	Single admission	7
2015	0	5.09E+00	0	2.36E+00	0	Repeated admissions	7
2015	6	5.06E+00	0	6.77E-01	0	Single admission	7
2015	13	5.03E+00	0	7.48E-01	0	Single admission	7
2015	1	5.02E+00	0	1.37E+00	0	Single admission	7
2015	4	5.01E+00	1	3.02E+00	0	Single admission	7
2015	1	4.99E+00	1	3.71E-01	0	Single admission	7
2015	11	4.99E+00	1	2.76E+00	0	Single admission	7
2015	14	4.94E+00	1	4.19E+00	0	Single admission	7
2015	10	4.92E+00	0	5.06E-01	0	Single admission	7
2015	10	4.89E+00	0	9.08E-01	0	Single admission	7
2015	15	4.87E+00	0	2.30E-01	0	Single admission	7
2015	9	4.85E+00	1	1.36E+00	0	Single admission	7
2015	10	4.84E+00	0	3.76E+00	0	Single admission	7
2015	6	4.84E+00	0	3.64E+00	0	Single admission	7
2015	14	4.83E+00	0	3.25E+00	0	Single admission	7
2015	3	4.79E+00	0	1.20E+00	0	Single admission	7
2015	1	4.75E+00	0	1.17E+00	0	Single admission	7
2015	2	4.73E+00	0	9.50E-01	0	Single admission	7
2015	0	6.25E-02	1	3.86E+00	1	Single admission	7
2015	17	4.67E+00	1	1.31E+00	0	Single admission	7
2015	0	4.67E+00	1	1.46E+00	0	Single admission	7
2015	14	4.66E+00	0	2.14E-01	0	Single admission	7

Admission year	Age (years)	Follow up time (years)	Sex (Females=1)	PDRLast	Outcome (Deceased=1)	Single/repeated admission	Diagnostic group
2015	7	4.64E+00	1	1.57E+00	0	Single admission	7
2015	1	4.64E+00	0	2.48E-01	0	Single admission	7
2015	7	4.57E+00	1	1.67E+00	0	Single admission	7
2015	9	4.55E+00	1	1.08E+00	0	Single admission	7
2015	4	4.55E+00	0	1.89E+00	0	Single admission	7
2015	1	4.50E+00	0	1.26E+00	0	Single admission	7
2015	17	5.47E+00	0	6.02E+01	0	Single admission	8
2015	16	5.46E+00	0	9.50E-01	0	Repeated admissions	8
2015	2	5.45E+00	0	7.51E-01	0	Single admission	8
2015	4	5.45E+00	1	9.37E-01	0	Single admission	8
2015	9	5.44E+00	0	1.56E-01	0	Single admission	8
2015	18	5.43E+00	0	8.16E-01	0	Single admission	8
2015	3	5.40E+00	1	7.51E-01	0	Single admission	8
2015	6	5.39E+00	1	8.39E-01	0	Single admission	8
2015	6	5.32E+00	0	1.10E+01	0	Single admission	8
2015	11	5.30E+00	0	8.16E-01	0	Single admission	8
2015	9	5.28E+00	1	3.71E-01	0	Single admission	8
2015	3	5.25E+00	1	7.51E-01	0	Single admission	8
2015	14	5.22E+00	1	3.66E+00	0	Single admission	8
2015	2	5.21E+00	0	3.94E-01	0	Single admission	8
2015	1	5.20E+00	0	6.20E+00	0	Single admission	8
2015	5	5.20E+00	0	9.77E-01	0	Repeated admissions	8
2015	17	5.20E+00	1	7.51E-01	0	Single admission	8
2015	1	5.14E+00	1	2.49E+01	0	Single admission	8
2015	15	5.13E+00	0	1.72E+00	0	Single admission	8
2015	2	5.13E+00	1	1.02E+00	0	Single admission	8
2015	2	5.11E+00	0	1.56E-01	0	Single admission	8
2015	16	5.10E+00	1	7.94E-01	0	Single admission	8
2015	1	5.09E+00	0	2.71E-01	0	Single admission	8
2015	2	5.09E+00	0	8.62E-01	0	Single admission	8

Admission year	Age (years)	Follow up time (years)	Sex (Females=1)	PDRLast	Outcome (Deceased=1)	Single/repeated admission	Diagnostic group
2015	0	5.06E+00	1	4.69E+00	0	Single admission	8
2015	16	5.06E+00	0	9.65E-01	0	Single admission	8
2015	6	9.71E-01	1	1.02E+00	1	Repeated admissions	8
2015	4	5.03E+00	0	1.84E+00	0	Single admission	8
2015	9	5.02E+00	0	1.11E+00	0	Single admission	8
2015	3	5.02E+00	0	1.60E+00	0	Single admission	8
2015	7	4.99E+00	1	9.90E-01	0	Single admission	8
2015	13	4.97E+00	1	9.80E-01	0	Single admission	8
2015	3	4.95E+00	0	1.23E+00	0	Single admission	8
2015	14	4.93E+00	0	8.62E-01	0	Single admission	8
2015	9	2.24E+00	0	1.25E+00	0	Single admission	8
2015	2	4.92E+00	0	1.12E+00	0	Single admission	8
2015	6	4.91E+00	1	4.28E-01	0	Single admission	8
2015	3	4.91E+00	0	1.57E+00	0	Single admission	8
2015	9	4.90E+00	1	1.14E+00	0	Single admission	8
2015	11	4.90E+00	1	7.22E+00	0	Single admission	8
2015	14	4.90E+00	1	6.86E+00	0	Single admission	8
2015	1	4.84E+00	0	9.90E-01	0	Single admission	8
2015	1	2.74E-04	0	9.82E+01	1	Single admission	8
2015	17	4.77E+00	1	9.30E-01	0	Single admission	8
2015	10	4.76E+00	0	8.62E-01	0	Single admission	8
2015	15	4.74E+00	1	1.21E+00	0	Single admission	8
2015	10	4.73E+00	0	3.17E-01	0	Single admission	8
2015	14	4.72E+00	0	9.22E-01	0	Single admission	8
2015	17	4.69E+00	0	2.71E-01	0	Single admission	8
2015	2	4.68E+00	1	7.51E-01	0	Single admission	8
2015	6	4.68E+00	0	8.93E+01	0	Single admission	8
2015	7	4.62E+00	0	5.08E+00	0	Single admission	8
2015	4	4.62E+00	0	1.25E+00	0	Single admission	8
2015	8	4.59E+00	1	1.87E+00	0	Single admission	8

Admission year	Age (years)	Follow up time (years)	Sex (Females=1)	PDRLast	Outcome (Deceased=1)	Single/repeated admission	Diagnostic group
2015	1	4.58E+00	0	9.72E-01	0	Single admission	8
2015	17	4.56E+00	0	1.15E+00	0	Single admission	8
2015	15	4.51E+00	1	9.40E-01	0	Single admission	8
2015	1	4.48E+00	1	4.23E-01	0	Single admission	8
2015	0	5.47E+00	0	2.71E-01	0	Single admission	9
2015	0	5.44E+00	0	2.12E+01	0	Single admission	9
2015	1	7.92E-01	1	8.20E-01	1	Single admission	9
2015	10	5.44E+00	1	2.43E-01	0	Single admission	9
2015	0	5.40E+00	0	1.95E+00	0	Single admission	9
2015	5	5.38E+00	0	4.52E-01	0	Repeated admissions	9
2015	10	5.38E+00	0	1.07E-01	0	Single admission	9
2015	0	5.34E+00	1	1.18E+00	0	Single admission	9
2015	10	5.33E+00	0	3.76E-01	0	Single admission	9
2015	10	5.65E-01	1	2.14E-01	1	Single admission	9
2015	1	5.25E+00	0	5.43E-01	0	Single admission	9
2015	15	5.25E+00	0	5.58E+00	0	Single admission	9
2015	10	5.21E+00	0	1.07E-01	0	Single admission	9
2015	12	5.17E+00	1	8.92E-01	0	Single admission	9
2015	8	5.16E+00	0	7.51E-01	0	Single admission	9
2015	4	5.13E+00	0	2.71E-01	0	Single admission	9
2015	14	5.12E+00	1	2.14E-01	0	Single admission	9
2015	17	5.11E+00	0	5.01E-01	0	Single admission	9
2015	3	5.06E+00	0	5.76E+00	0	Single admission	9
2015	15	5.06E+00	1	1.82E+00	0	Single admission	9
2015	7	1.31E+00	1	2.03E-01	1	Single admission	9
2015	6	5.04E+00	1	1.14E+01	0	Repeated admissions	9
2015	0	5.02E+00	0	1.02E+00	0	Single admission	9
2015	0	4.96E+00	0	2.96E+00	0	Single admission	9
2015	0	4.96E+00	0	2.58E-01	0	Repeated admissions	9
2015	0	4.85E+00	0	6.77E-01	0	Single admission	9

Admission year	Age (years)	Follow up time (years)	Sex (Females=1)	PDRLast	Outcome (Deceased=1)	Single/repeated admission	Diagnostic group
2015	8	4.85E+00	0	5.74E-01	0	Single admission	9
2015	17	4.83E+00	1	1.15E-01	0	Repeated admissions	9
2015	1	8.12E-01	0	2.51E+00	1	Repeated admissions	9
2015	9	4.79E+00	1	1.32E+00	0	Single admission	9
2015	0	4.78E+00	0	1.07E-01	0	Single admission	9
2015	13	4.76E+00	1	5.72E-01	0	Repeated admissions	9
2015	0	4.75E+00	0	1.72E-01	0	Single admission	9
2015	11	4.75E+00	1	2.49E-01	0	Single admission	9
2015	1	4.75E+00	1	9.63E-01	0	Single admission	9
2015	9	4.70E+00	1	2.98E-01	0	Repeated admissions	9
2015	5	4.68E+00	0	3.08E-01	0	Single admission	9
2015	5	4.61E+00	0	2.93E-01	0	Single admission	9
2015	10	4.58E+00	1	1.82E+00	0	Single admission	9
2015	0	4.53E+00	1	2.16E-01	0	Single admission	9
2015	2	5.25E+00	0	1.32E+00	0	Single admission	10
2015	1	5.47E+00	1	7.26E-01	0	Single admission	10
2015	1	5.46E+00	0	1.19E+00	0	Single admission	10
2015	4	5.43E+00	0	3.98E+00	0	Repeated admissions	10
2015	8	5.43E+00	1	7.94E-01	0	Single admission	10
2015	2	5.39E+00	0	5.44E+00	0	Single admission	10
2015	11	5.38E+00	1	3.97E+00	0	Single admission	10
2015	13	5.35E+00	0	1.17E+00	0	Single admission	10
2015	1	5.32E+00	1	3.92E+00	0	Single admission	10
2015	0	5.31E+00	0	1.93E+00	0	Single admission	10
2015	7	5.29E+00	0	1.55E+00	0	Single admission	10
2015	12	5.29E+00	1	9.50E-01	0	Repeated admissions	10
2015	3	5.28E+00	1	2.49E+01	0	Single admission	10
2015	9	8.91E-01	0	1.06E+00	1	Repeated admissions	10
2015	16	5.23E+00	0	3.56E+00	0	Single admission	10
2015	3	5.18E+00	1	1.02E+00	0	Single admission	10

Admission year	Age (years)	Follow up time (years)	Sex (Females=1)	PDRLast	Outcome (Deceased=1)	Single/repeated admission	Diagnostic group
2015	2	5.16E+00	1	3.44E-01	0	Repeated admissions	10
2015	3	5.14E+00	0	2.38E-01	0	Single admission	10
2015	9	5.13E+00	1	7.94E-01	0	Single admission	10
2015	11	5.12E+00	0	8.05E-01	0	Single admission	10
2015	8	5.09E+00	0	9.83E-01	0	Single admission	10
2015	12	8.05E-02	1	7.51E-01	0	Single admission	10
2015	5	5.00E+00	0	5.71E+00	0	Single admission	10
2015	1	4.98E+00	1	4.65E+00	0	Single admission	10
2015	0	3.95E+00	0	3.47E+00	1	Single admission	10
2015	18	3.94E+00	1	6.26E+00	1	Single admission	10
2015	7	4.92E+00	0	7.94E-01	0	Single admission	10
2015	5	1.38E+00	1	5.60E+00	1	Single admission	10
2015	1	4.57E+00	1	8.06E+00	1	Repeated admissions	10
2015	13	4.88E+00	0	2.34E+00	0	Single admission	10
2015	6	4.88E+00	1	6.38E+00	0	Single admission	10
2015	6	4.83E+00	0	8.16E-01	0	Single admission	10
2015	1	4.81E+00	0	9.92E-01	0	Single admission	10
2015	5	4.79E+00	0	9.63E-01	0	Single admission	10
2015	1	4.76E+00	0	7.51E-01	0	Single admission	10
2015	1	4.75E+00	1	2.32E+00	0	Single admission	10
2015	13	4.75E+00	0	1.09E+00	0	Single admission	10
2015	12	4.73E+00	0	7.51E-01	0	Repeated admissions	10
2015	0	3.83E+00	0	1.86E+00	0	Single admission	10
2015	10	4.72E+00	0	7.34E+00	0	Single admission	10
2015	1	4.71E+00	1	1.50E+00	0	Single admission	10
2015	0	4.70E+00	0	1.25E+01	0	Single admission	10
2015	2	4.65E+00	0	1.71E+00	0	Single admission	10
2015	5	4.61E+00	0	8.05E-01	0	Single admission	10
2015	1	4.60E+00	0	1.30E+00	0	Single admission	10
2015	9	4.60E+00	0	1.19E+00	0	Single admission	10

Admission year	Age (years)	Follow up time (years)	Sex (Females=1)	PDRLast	Outcome (Deceased=1)	Single/repeated admission	Diagnostic group
2015	2	4.58E+00	1	5.76E+00	0	Single admission	10
2015	1	5.79E-01	1	1.70E+00	1	Repeated admissions	10
2015	0	2.76E+00	0	1.73E+00	1	Single admission	10
2015	2	4.53E+00	1	1.02E+00	0	Single admission	10
2015	0	4.51E+00	0	1.35E+00	0	Single admission	10
2015	15	4.50E+00	0	5.61E-02	0	Single admission	10
2015	1	4.49E+00	1	1.63E+00	0	Single admission	10
2015	0	5.47E+00	1	1.01E+01	0	Single admission	11
2015	0	5.46E+00	1	1.28E+00	0	Single admission	11
2015	0	5.44E+00	1	4.10E-01	0	Single admission	11
2015	0	5.41E+00	0	8.51E+00	0	Single admission	11
2015	0	5.40E+00	0	1.44E+00	0	Repeated admissions	11
2015	0	5.38E+00	0	1.05E+01	0	Single admission	11
2015	1	5.35E+00	1	1.17E+00	0	Single admission	11
2015	0	5.34E+00	0	2.30E+00	0	Repeated admissions	11
2015	0	5.33E+00	0	8.83E-01	0	Repeated admissions	11
2015	1	5.32E+00	1	1.07E-01	0	Repeated admissions	11
2015	0	5.29E+00	1	7.51E-01	0	Single admission	11
2015	0	7.10E-01	1	1.20E-01	0	Single admission	11
2015	1	5.25E-01	1	5.31E+00	1	Repeated admissions	11
2015	0	5.26E+00	1	2.40E+00	0	Single admission	11
2015	1	5.25E+00	0	8.33E-01	0	Repeated admissions	11
2015	0	5.24E+00	0	1.15E+00	0	Single admission	11
2015	0	5.23E+00	1	2.47E-01	0	Repeated admissions	11
2015	0	5.21E+00	1	5.98E+00	0	Repeated admissions	11
2015	0	5.18E+00	1	2.21E+00	0	Repeated admissions	11
2015	0	5.17E+00	1	2.98E-01	0	Single admission	11
2015	5	5.15E+00	1	6.30E-01	0	Single admission	11
2015	0	5.15E+00	0	1.11E+01	0	Single admission	11
2015	0	5.12E+00	0	2.23E+00	0	Repeated admissions	11

Admission year	Age (years)	Follow up time (years)	Sex (Females=1)	PDRLast	Outcome (Deceased=1)	Single/repeated admission	Diagnostic group
2015	0	5.08E+00	0	1.04E+00	0	Single admission	11
2015	0	5.08E+00	1	2.23E+01	0	Single admission	11
2015	0	5.06E+00	0	4.27E+01	0	Single admission	11
2015	0	5.04E+00	0	1.87E-01	0	Repeated admissions	11
2015	16	5.02E+00	0	1.14E+00	0	Single admission	11
2015	11	5.02E+00	1	1.79E-01	0	Single admission	11
2015	0	5.01E+00	1	8.87E+00	0	Single admission	11
2015	0	5.01E+00	1	1.90E+01	0	Single admission	11
2015	0	5.00E+00	1	1.71E+00	0	Single admission	11
2015	0	5.00E+00	0	1.42E+00	0	Repeated admissions	11
2015	0	4.99E+00	0	8.05E+00	0	Single admission	11
2015	0	4.97E+00	0	7.35E-01	0	Single admission	11
2015	0	4.94E+00	1	2.74E+00	0	Single admission	11
2015	0	4.89E+00	0	2.35E+00	0	Single admission	11
2015	0	4.87E+00	1	3.24E+00	0	Single admission	11
2015	0	4.87E+00	0	1.38E+01	0	Single admission	11
2015	0	4.85E+00	1	6.82E+00	0	Single admission	11
2015	0	4.81E+00	0	2.12E+00	0	Single admission	11
2015	0	4.81E+00	1	6.35E+00	0	Single admission	11
2015	0	4.80E+00	0	4.27E+00	0	Single admission	11
2015	0	4.79E+00	0	8.05E-01	0	Repeated admissions	11
2015	0	4.77E+00	0	2.33E+00	0	Repeated admissions	11
2015	2	4.77E+00	0	2.71E-01	0	Single admission	11
2015	0	4.75E+00	1	7.51E-01	0	Repeated admissions	11
2015	0	4.74E+00	1	7.51E-01	0	Single admission	11
2015	0	4.74E+00	1	9.24E-01	0	Single admission	11
2015	0	4.73E+00	1	5.06E-01	0	Single admission	11
2015	0	4.72E+00	0	1.02E+00	0	Single admission	11
2015	0	4.70E+00	0	1.07E-01	0	Single admission	11
2015	0	4.69E+00	0	8.53E+00	0	Single admission	11

Admission year	Age (years)	Follow up time (years)	Sex (Females=1)	PDRLast	Outcome (Deceased=1)	Single/repeated admission	Diagnostic group
2015	0	4.68E+00	1	1.65E+00	0	Single admission	11
2015	0	4.67E+00	1	1.36E+00	0	Single admission	11
2015	0	4.66E+00	0	1.27E+00	0	Single admission	11
2015	12	4.65E+00	1	2.71E-01	0	Single admission	11
2015	0	4.65E+00	1	1.34E+00	0	Single admission	11
2015	0	4.64E+00	0	2.88E+01	0	Single admission	11
2015	0	4.64E+00	1	1.90E-01	0	Single admission	11
2015	0	4.63E+00	0	1.37E+00	0	Repeated admissions	11
2015	1	4.62E+00	0	6.44E+00	0	Repeated admissions	11
2015	0	4.61E+00	0	1.76E+00	0	Single admission	11
2015	2	4.59E+00	0	6.04E-01	0	Single admission	11
2015	2	4.57E+00	1	5.26E+00	0	Single admission	11
2015	0	4.57E+00	1	5.88E-01	0	Repeated admissions	11
2015	0	4.57E+00	1	6.02E+00	0	Repeated admissions	11
2015	0	4.54E+00	0	6.76E+00	0	Single admission	11
2015	0	4.54E+00	0	2.16E+00	0	Single admission	11
2015	6	4.54E+00	0	1.67E-01	0	Single admission	11
2015	0	4.54E+00	0	7.51E-01	0	Repeated admissions	11
2015	2	5.47E+00	0	2.09E-01	0	Single admission	12
2015	2	5.46E+00	0	3.38E+00	0	Single admission	12
2015	0	5.45E+00	0	9.49E-01	0	Single admission	12
2015	0	5.45E+00	1	1.64E-01	0	Single admission	12
2015	0	5.45E+00	1	1.03E+00	0	Single admission	12
2015	0	5.45E+00	1	1.18E+00	0	Single admission	12
2015	1	5.44E+00	0	6.92E+00	0	Repeated admissions	12
2015	3	5.44E+00	0	1.30E+00	0	Repeated admissions	12
2015	0	5.44E+00	1	3.17E-01	0	Single admission	12
2015	0	5.43E+00	0	7.28E+00	0	Single admission	12
2015	0	5.43E+00	0	1.35E+00	0	Single admission	12
2015	0	5.43E+00	0	8.68E+00	0	Single admission	12

Admission year	Age (years)	Follow up time (years)	Sex (Females=1)	PDRLast	Outcome (Deceased=1)	Single/repeated admission	Diagnostic group
2015	0	5.41E+00	0	6.51E-01	0	Single admission	12
2015	0	5.41E+00	0	8.05E-01	0	Single admission	12
2015	0	7.53E-02	0	9.93E+01	1	Repeated admissions	12
2015	0	5.40E+00	1	5.91E-01	0	Single admission	12
2015	0	5.40E+00	0	3.66E-01	0	Single admission	12
2015	2	5.40E+00	1	4.80E+00	0	Single admission	12
2015	1	5.39E+00	0	1.56E-01	0	Single admission	12
2015	0	5.39E+00	1	1.95E+00	0	Single admission	12
2015	0	5.38E+00	0	1.26E+00	0	Single admission	12
2015	0	5.37E+00	1	1.15E+00	0	Single admission	12
2015	0	5.37E+00	0	3.36E+00	0	Single admission	12
2015	0	5.37E+00	1	1.13E+00	0	Single admission	12
2015	1	5.37E+00	0	7.51E-01	0	Single admission	12
2015	0	2.59E+00	0	4.05E+00	0	Single admission	12
2015	0	5.37E+00	0	2.55E+00	0	Single admission	12
2015	0	5.36E+00	0	8.10E-01	0	Single admission	12
2015	2	5.36E+00	0	2.35E-01	0	Repeated admissions	12
2015	0	5.36E+00	0	5.91E-01	0	Single admission	12
2015	0	5.36E+00	0	1.56E-01	0	Single admission	12
2015	5	3.64E-02	0	7.56E+00	1	Single admission	12
2015	0	5.35E+00	0	1.04E+00	0	Single admission	12
2015	0	5.32E+00	1	1.11E+00	0	Single admission	12
2015	0	5.32E+00	0	7.27E-01	0	Single admission	12
2015	0	5.32E+00	1	1.10E+00	0	Single admission	12
2015	2	5.32E+00	1	1.56E-01	0	Single admission	12
2015	0	5.31E+00	0	1.84E-01	0	Single admission	12
2015	0	5.31E+00	0	5.13E-01	0	Single admission	12
2015	0	5.31E+00	0	2.54E-01	0	Single admission	12
2015	0	5.30E+00	1	2.84E+00	0	Single admission	12
2015	1	5.30E+00	0	5.91E-01	0	Single admission	12

Admission year	Age (years)	Follow up time (years)	Sex (Females=1)	PDRLast	Outcome (Deceased=1)	Single/repeated admission	Diagnostic group
2015	1	5.29E+00	1	4.05E-01	0	Single admission	12
2015	2	5.28E+00	0	2.35E-01	0	Single admission	12
2015	0	5.28E+00	0	2.04E-01	0	Repeated admissions	12
2015	1	5.27E+00	0	3.60E-01	0	Single admission	12
2015	13	6.96E-03	0	3.33E+01	1	Single admission	12
2015	0	5.25E+00	0	8.13E-01	0	Single admission	12
2015	0	5.25E+00	0	6.55E-01	0	Single admission	12
2015	2	9.47E-03	0	7.32E+01	1	Single admission	12
2015	7	5.24E+00	1	1.03E+00	0	Single admission	12
2015	0	5.24E+00	1	1.10E+00	0	Single admission	12
2015	9	2.76E+00	1	1.54E+00	1	Repeated admissions	12
2015	0	5.21E+00	0	1.58E+00	0	Single admission	12
2015	0	5.20E+00	1	2.71E-01	0	Single admission	12
2015	6	5.20E+00	0	9.90E-01	0	Single admission	12
2015	0	5.19E+00	0	4.23E+00	0	Single admission	12
2015	1	5.18E+00	0	1.49E+00	0	Single admission	12
2015	6	4.97E-02	0	2.05E+01	1	Single admission	12
2015	0	8.39E-01	1	1.80E-01	1	Single admission	12
2015	0	5.12E+00	0	1.48E+00	0	Single admission	12
2015	0	5.09E+00	1	2.80E+00	0	Single admission	12
2015	5	5.07E+00	0	4.19E+00	0	Single admission	12
2015	0	5.07E+00	0	1.94E+00	0	Single admission	12
2015	0	5.06E+00	1	6.29E+00	0	Single admission	12
2015	2	5.04E+00	0	9.49E-01	0	Single admission	12
2015	0	5.04E+00	1	7.51E-01	0	Single admission	12
2015	0	5.02E+00	1	2.80E+00	0	Single admission	12
2015	0	5.02E+00	1	1.58E+00	0	Repeated admissions	12
2015	1	4.96E+00	1	8.33E-01	0	Repeated admissions	12
2015	0	4.96E+00	0	2.70E-01	0	Single admission	12
2015	2	4.96E+00	1	1.44E+01	0	Repeated admissions	12

Admission year	Age (years)	Follow up time (years)	Sex (Females=1)	PDRLast	Outcome (Deceased=1)	Single/repeated admission	Diagnostic group
2015	9	4.95E+00	0	2.64E+00	0	Single admission	12
2015	1	4.94E+00	1	7.22E+00	0	Single admission	12
2015	2	4.90E+00	0	7.51E-01	0	Repeated admissions	12
2015	0	4.90E+00	0	1.75E+00	0	Single admission	12
2015	4	4.88E+00	1	8.33E+00	0	Single admission	12
2015	3	4.87E+00	0	4.03E+00	0	Single admission	12
2015	5	5.82E-03	1	1.98E+00	1	Repeated admissions	12
2015	3	4.84E+00	1	2.37E-01	0	Repeated admissions	12
2015	4	4.83E+00	0	1.80E-01	0	Single admission	12
2015	1	4.79E+00	0	4.94E+00	0	Repeated admissions	12
2015	1	4.75E+00	0	3.58E-01	0	Single admission	12
2015	0	4.73E+00	0	7.64E+00	0	Single admission	12
2015	6	4.70E+00	1	2.24E-01	0	Single admission	12
2015	18	4.68E+00	0	1.90E+00	0	Single admission	12
2015	5	4.68E+00	1	4.48E+00	0	Single admission	12
2015	0	4.67E+00	0	1.52E+00	0	Single admission	12
2015	2	4.66E+00	1	9.04E-01	0	Single admission	12
2015	2	4.66E+00	0	6.42E+00	0	Single admission	12
2015	0	4.65E+00	1	2.43E+00	0	Single admission	12
2015	1	4.64E+00	0	1.73E-01	0	Single admission	12
2015	5	4.61E+00	0	6.72E-02	0	Single admission	12
2015	4	4.61E+00	1	4.56E+00	0	Single admission	12
2015	1	4.60E+00	0	3.29E-01	0	Single admission	12
2015	0	4.60E+00	0	5.91E-01	0	Single admission	12
2015	0	9.53E-01	0	2.24E+01	1	Single admission	12
2015	1	4.58E+00	1	2.24E-01	0	Single admission	12
2015	0	4.55E+00	1	8.58E-01	0	Single admission	12
2015	2	4.55E+00	1	9.30E-01	0	Single admission	12
2015	1	4.55E+00	0	2.80E+00	0	Repeated admissions	12
2015	3	4.53E+00	0	2.98E-01	0	Repeated admissions	12

Admission year	Age (years)	Follow up time (years)	Sex (Females=1)	PDRLast	Outcome (Deceased=1)	Single/repeated admission	Diagnostic group
2015	3	4.53E+00	1	2.04E-01	0	Single admission	12
2015	0	4.51E+00	0	1.27E+00	0	Single admission	12
2015	0	4.50E+00	0	4.74E-01	0	Single admission	12
2015	4	4.49E+00	1	3.79E+00	0	Single admission	12
2015	0	5.37E+00	0	5.91E-01	0	Single admission	13
2016	3	4.24E+00	1	2.64E+00	0	Single admission	1
2016	0	4.06E+00	1	7.51E-01	0	Single admission	1
2016	0	4.36E+00	0	1.60E+00	0	Single admission	2
2016	0	4.36E+00	1	2.80E+00	0	Single admission	2
2016	0	4.23E+00	0	2.87E+00	0	Single admission	2
2016	0	1.09E-02	0	9.63E+00	1	Single admission	2
2016	0	4.11E+00	0	9.94E+00	0	Single admission	2
2016	0	4.08E+00	1	2.55E+01	0	Single admission	2
2016	0	4.06E+00	0	6.66E+00	0	Single admission	2
2016	0	1.54E-01	0	1.15E+01	1	Single admission	2
2016	0	3.83E+00	1	1.00E+00	0	Single admission	2
2016	0	3.65E+00	1	7.51E-01	0	Single admission	2
2016	0	3.65E+00	0	7.51E-01	0	Single admission	2
2016	0	3.56E+00	0	7.51E-01	0	Single admission	2
2016	0	3.48E+00	1	7.51E-01	0	Single admission	2
2016	8	4.46E+00	1	5.41E-01	0	Single admission	3
2016	1	4.30E+00	0	4.47E+00	0	Single admission	3
2016	0	7.73E-01	0	2.31E+00	1	Single admission	3
2016	1	4.06E+00	0	8.22E-01	0	Repeated admissions	3
2016	15	3.92E+00	0	8.95E-01	0	Single admission	3
2016	9	3.74E+00	1	3.03E+00	0	Single admission	3
2016	1	2.47E-01	1	7.51E-01	1	Repeated admissions	3
2016	12	8.33E-01	1	7.51E-01	1	Single admission	3
2016	3	3.62E+00	0	7.51E-01	0	Single admission	3
2016	1	3.56E+00	1	7.51E-01	0	Single admission	3

Admission year	Age (years)	Follow up time (years)	Sex (Females=1)	PDRLast	Outcome (Deceased=1)	Single/repeated admission	Diagnostic group
2016	0	3.55E+00	0	7.51E-01	0	Single admission	3
2016	0	3.52E+00	1	7.51E-01	0	Single admission	3
2016	6	3.48E+00	0	7.51E-01	0	Single admission	3
2016	1	3.48E+00	1	7.51E-01	0	Single admission	3
2016	0	4.46E+00	0	7.31E+00	0	Single admission	4
2016	4	4.43E+00	1	8.33E-01	0	Repeated admissions	4
2016	0	4.43E+00	1	1.20E+00	0	Single admission	4
2016	0	4.42E+00	1	7.51E-01	0	Single admission	4
2016	2	4.38E+00	1	1.84E+00	0	Single admission	4
2016	10	4.37E+00	0	1.56E+00	0	Single admission	4
2016	2	4.24E+00	0	2.49E+00	0	Single admission	4
2016	3	4.24E+00	1	3.81E+00	0	Single admission	4
2016	1	4.18E+00	1	9.82E-01	0	Repeated admissions	4
2016	6	4.10E+00	1	2.45E+00	0	Single admission	4
2016	0	4.09E+00	1	9.32E-01	0	Single admission	4
2016	0	4.42E-03	0	1.54E+01	1	Single admission	4
2016	0	4.04E+00	0	1.51E+00	0	Single admission	4
2016	9	4.01E+00	0	7.26E+00	0	Single admission	4
2016	0	4.00E+00	1	7.51E-01	0	Single admission	4
2016	0	9.72E-03	0	5.68E+00	1	Single admission	4
2016	5	3.81E+00	1	7.51E-01	0	Single admission	4
2016	0	3.75E+00	1	1.67E+00	0	Repeated admissions	4
2016	6	3.74E+00	1	1.20E+00	0	Single admission	4
2016	1	3.73E+00	1	1.37E+00	0	Single admission	4
2016	0	3.72E+00	0	7.51E-01	0	Single admission	4
2016	1	3.70E+00	0	7.51E-01	0	Single admission	4
2016	0	1.03E-01	1	7.51E-01	1	Single admission	4
2016	1	3.69E+00	0	7.51E-01	0	Repeated admissions	4
2016	0	3.67E+00	0	7.51E-01	0	Single admission	4
2016	5	1.89E-02	0	7.51E-01	1	Single admission	4

Admission year	Age (years)	Follow up time (years)	Sex (Females=1)	PDRLast	Outcome (Deceased=1)	Single/repeated admission	Diagnostic group
2016	4	3.65E+00	1	7.51E-01	0	Single admission	4
2016	0	3.63E+00	0	7.51E-01	0	Repeated admissions	4
2016	5	3.56E+00	0	7.51E-01	0	Single admission	4
2016	5	3.56E+00	0	7.51E-01	0	Single admission	4
2016	1	3.51E+00	0	7.51E-01	0	Single admission	4
2016	2	3.48E+00	0	7.51E-01	0	Single admission	4
2016	11	4.47E+00	0	1.71E-01	0	Single admission	5
2016	6	4.44E+00	0	5.91E-01	0	Single admission	5
2016	1	3.27E-01	0	2.00E+00	1	Single admission	5
2016	3	4.40E+00	0	2.84E-01	0	Single admission	5
2016	0	1.34E-01	1	6.32E+00	1	Single admission	5
2016	13	4.35E+00	1	4.64E-01	0	Repeated admissions	5
2016	13	1.87E+00	0	3.53E-01	1	Repeated admissions	5
2016	9	3.24E+00	0	3.75E-01	1	Single admission	5
2016	7	4.26E+00	0	3.73E+00	0	Single admission	5
2016	1	1.02E+00	0	9.24E-01	1	Repeated admissions	5
2016	5	4.21E+00	0	4.36E-01	0	Single admission	5
2016	12	4.10E+00	1	4.88E-01	0	Single admission	5
2016	1	4.09E+00	0	4.46E+00	0	Repeated admissions	5
2016	3	4.08E+00	0	1.20E+00	0	Single admission	5
2016	3	4.07E+00	0	9.77E-01	0	Single admission	5
2016	5	4.06E+00	1	7.51E-01	0	Repeated admissions	5
2016	11	4.06E+00	0	3.02E-01	0	Single admission	5
2016	13	1.53E+00	1	1.73E+00	1	Single admission	5
2016	0	3.89E+00	0	2.10E+00	0	Single admission	5
2016	14	3.88E+00	0	8.33E-01	0	Single admission	5
2016	0	3.88E+00	0	8.51E-01	0	Single admission	5
2016	0	3.83E+00	0	5.46E+00	0	Single admission	5
2016	8	3.82E+00	1	1.04E+00	0	Single admission	5
2016	3	3.79E+00	1	9.24E-01	0	Single admission	5

Admission year	Age (years)	Follow up time (years)	Sex (Females=1)	PDRLast	Outcome (Deceased=1)	Single/repeated admission	Diagnostic group
2016	1	3.78E+00	1	9.20E-01	0	Single admission	5
2016	3	3.75E+00	1	4.72E-01	0	Single admission	5
2016	0	3.72E+00	0	9.87E+00	0	Single admission	5
2016	9	3.68E+00	0	7.51E-01	0	Single admission	5
2016	11	1.31E+00	1	7.51E-01	1	Single admission	5
2016	0	3.27E-01	1	7.51E-01	1	Single admission	5
2016	7	3.60E+00	1	7.51E-01	0	Single admission	5
2016	11	6.05E-01	0	7.51E-01	1	Single admission	5
2016	0	3.56E+00	1	7.51E-01	0	Repeated admissions	5
2016	1	3.14E+00	1	7.51E-01	1	Single admission	5
2016	0	4.44E+00	1	7.65E+01	0	Single admission	6
2016	13	8.64E-03	1	1.77E+01	1	Single admission	6
2016	0	4.36E+00	0	2.10E+00	0	Single admission	6
2016	14	4.34E+00	1	1.50E+00	0	Single admission	6
2016	14	4.33E+00	1	4.81E-01	0	Single admission	6
2016	7	4.33E+00	0	4.62E+00	0	Single admission	6
2016	0	4.27E+00	1	7.51E-01	0	Single admission	6
2016	0	4.18E+00	0	1.20E+01	0	Single admission	6
2016	8	4.14E+00	0	1.46E+00	0	Single admission	6
2016	0	4.06E+00	1	5.61E+00	0	Repeated admissions	6
2016	0	4.05E+00	1	1.30E+00	0	Single admission	6
2016	15	3.99E+00	1	8.42E-01	0	Single admission	6
2016	0	3.97E+00	1	1.14E+00	0	Single admission	6
2016	2	3.95E+00	1	7.51E-01	0	Repeated admissions	6
2016	1	2.55E-01	0	1.07E+01	1	Single admission	6
2016	2	3.93E+00	1	1.23E+00	0	Single admission	6
2016	0	3.86E+00	1	1.22E+01	0	Single admission	6
2016	14	3.85E+00	0	1.26E+00	0	Single admission	6
2016	9	3.80E+00	0	7.51E-01	0	Repeated admissions	6
2016	17	3.80E+00	0	9.77E-01	0	Single admission	6

Admission year	Age (years)	Follow up time (years)	Sex (Females=1)	PDRLast	Outcome (Deceased=1)	Single/repeated admission	Diagnostic group
2016	1	3.77E+00	1	6.63E+00	0	Single admission	6
2016	0	3.77E+00	1	7.51E-01	0	Single admission	6
2016	0	3.77E+00	0	6.14E-01	0	Single admission	6
2016	18	3.64E+00	1	7.51E-01	0	Single admission	6
2016	11	3.60E+00	1	7.51E-01	0	Single admission	6
2016	0	4.88E-02	0	7.51E-01	1	Single admission	6
2016	0	3.59E+00	0	7.51E-01	0	Single admission	6
2016	7	3.58E+00	0	7.51E-01	0	Single admission	6
2016	9	3.56E+00	0	7.51E-01	0	Single admission	6
2016	4	4.44E+00	1	7.58E-01	0	Single admission	7
2016	0	3.07E+00	1	1.61E+01	1	Single admission	7
2016	9	4.43E+00	1	1.23E+01	0	Single admission	7
2016	14	4.41E+00	0	1.09E+00	0	Single admission	7
2016	9	4.39E+00	1	2.58E+00	0	Single admission	7
2016	2	4.35E+00	1	1.44E+01	0	Single admission	7
2016	5	4.31E+00	1	7.00E+00	0	Single admission	7
2016	0	4.30E+00	0	1.38E+00	0	Repeated admissions	7
2016	2	4.30E+00	0	5.21E+00	0	Single admission	7
2016	7	3.17E+00	1	3.78E+00	0	Single admission	7
2016	8	4.27E+00	0	8.47E+00	0	Single admission	7
2016	7	4.25E+00	1	1.38E+01	0	Single admission	7
2016	0	4.20E+00	0	1.39E+01	0	Single admission	7
2016	12	4.15E+00	0	3.10E+00	0	Single admission	7
2016	12	4.14E+00	1	3.86E+00	0	Single admission	7
2016	0	4.09E+00	0	3.89E+00	0	Single admission	7
2016	1	4.07E+00	0	1.01E+01	0	Single admission	7
2016	2	4.05E+00	1	1.28E+01	0	Single admission	7
2016	13	4.04E+00	0	6.02E+00	0	Single admission	7
2016	15	4.03E+00	0	3.15E+00	0	Single admission	7
2016	11	4.01E+00	1	4.98E+00	0	Single admission	7

Admission year	Age (years)	Follow up time (years)	Sex (Females=1)	PDRLast	Outcome (Deceased=1)	Single/repeated admission	Diagnostic group
2016	2	3.86E+00	1	7.76E+00	0	Single admission	7
2016	18	3.83E+00	1	6.96E+00	0	Single admission	7
2016	6	5.47E-03	0	1.96E+00	1	Single admission	7
2016	4	3.80E+00	0	1.40E+01	0	Single admission	7
2016	7	3.78E+00	0	4.77E+00	0	Single admission	7
2016	10	3.70E+00	1	7.51E-01	0	Repeated admissions	7
2016	12	3.69E+00	0	7.51E-01	0	Single admission	7
2016	18	3.69E+00	0	7.51E-01	0	Single admission	7
2016	1	3.65E+00	1	7.51E-01	0	Single admission	7
2016	1	3.65E+00	0	7.51E-01	0	Single admission	7
2016	1	3.64E+00	1	7.51E-01	0	Single admission	7
2016	6	3.63E+00	0	7.51E-01	0	Single admission	7
2016	13	3.60E+00	0	7.51E-01	0	Single admission	7
2016	17	3.59E+00	1	7.51E-01	0	Single admission	7
2016	2	3.59E+00	0	7.51E-01	0	Single admission	7
2016	0	3.58E+00	0	7.51E-01	0	Single admission	7
2016	16	4.48E+00	1	3.34E+00	0	Single admission	8
2016	11	4.46E+00	0	7.83E-01	0	Single admission	8
2016	1	4.45E+00	0	1.38E+00	0	Single admission	8
2016	11	4.43E+00	0	1.08E+00	0	Single admission	8
2016	3	4.42E+00	0	8.74E-01	0	Single admission	8
2016	8	4.41E+00	1	5.16E+00	0	Single admission	8
2016	3	4.40E+00	1	1.36E+00	0	Single admission	8
2016	12	4.38E+00	0	4.17E+00	0	Single admission	8
2016	8	4.35E+00	1	8.14E+00	0	Single admission	8
2016	6	4.35E+00	0	1.06E+00	0	Single admission	8
2016	9	4.34E+00	0	8.80E-01	0	Single admission	8
2016	4	4.33E+00	0	1.05E+00	0	Single admission	8
2016	1	4.33E+00	0	2.80E+00	0	Single admission	8
2016	8	4.32E+00	1	3.20E+00	0	Single admission	8

Admission year	Age (years)	Follow up time (years)	Sex (Females=1)	PDRLast	Outcome (Deceased=1)	Single/repeated admission	Diagnostic group
2016	3	4.32E+00	1	1.30E+00	0	Single admission	8
2016	14	4.19E+00	0	7.94E-01	0	Single admission	8
2016	14	4.18E+00	0	9.59E-01	0	Single admission	8
2016	2	4.15E+00	0	1.17E+00	0	Single admission	8
2016	9	4.15E+00	0	1.00E+00	0	Single admission	8
2016	10	4.13E+00	1	7.51E-01	0	Repeated admissions	8
2016	14	4.09E+00	0	9.53E-01	0	Single admission	8
2016	5	4.09E+00	1	3.73E+00	0	Single admission	8
2016	13	1.03E-03	0	5.20E+01	1	Single admission	8
2016	4	9.70E-01	1	1.55E+00	0	Single admission	8
2016	14	4.03E+00	0	3.73E+00	0	Single admission	8
2016	14	4.03E+00	0	8.16E-01	0	Single admission	8
2016	8	3.90E+00	0	8.50E-01	0	Single admission	8
2016	14	3.89E+00	0	3.84E+00	0	Single admission	8
2016	4	3.89E+00	1	1.35E+00	0	Single admission	8
2016	13	3.88E+00	0	4.11E+00	0	Single admission	8
2016	2	3.87E+00	0	7.51E-01	0	Repeated admissions	8
2016	1	3.86E+00	1	1.19E+01	0	Single admission	8
2016	8	3.82E+00	0	2.00E+00	0	Single admission	8
2016	0	3.82E+00	0	1.69E+00	0	Single admission	8
2016	12	3.79E+00	1	9.50E-01	0	Single admission	8
2016	14	3.77E+00	0	8.16E-01	0	Single admission	8
2016	2	3.75E+00	0	9.89E-01	0	Single admission	8
2016	4	3.75E+00	0	1.19E+00	0	Single admission	8
2016	3	3.73E+00	0	1.80E+00	0	Single admission	8
2016	14	9.12E-01	1	7.51E-01	1	Single admission	8
2016	14	3.70E+00	1	7.51E-01	0	Repeated admissions	8
2016	18	3.70E+00	1	7.51E-01	0	Repeated admissions	8
2016	3	3.69E+00	1	7.51E-01	0	Single admission	8
2016	2	3.67E+00	1	7.51E-01	0	Single admission	8

Admission year	Age (years)	Follow up time (years)	Sex (Females=1)	PDRLast	Outcome (Deceased=1)	Single/repeated admission	Diagnostic group
2016	0	3.60E+00	0	7.51E-01	0	Single admission	8
2016	0	4.45E+00	0	5.13E-01	0	Single admission	9
2016	13	4.44E+00	1	2.56E+00	0	Single admission	9
2016	17	4.40E+00	1	3.09E-01	0	Single admission	9
2016	14	4.17E+00	1	2.54E+00	0	Single admission	9
2016	0	4.12E+00	0	4.39E+00	0	Repeated admissions	9
2016	13	4.10E+00	1	4.99E-01	0	Single admission	9
2016	14	4.06E+00	1	4.84E-01	0	Single admission	9
2016	12	4.04E+00	1	6.45E-01	0	Single admission	9
2016	13	3.83E+00	1	5.04E-01	0	Single admission	9
2016	7	3.83E+00	0	3.52E-01	0	Single admission	9
2016	0	3.79E+00	0	5.65E-01	0	Single admission	9
2016	10	3.75E+00	1	3.73E-01	0	Single admission	9
2016	14	2.59E+00	0	7.51E-01	1	Single admission	9
2016	17	4.46E+00	0	7.61E-01	0	Single admission	10
2016	14	4.45E+00	1	1.06E+00	0	Single admission	10
2016	2	4.41E+00	1	8.51E-01	0	Single admission	10
2016	0	4.40E+00	0	1.11E+00	0	Single admission	10
2016	1	4.39E+00	1	1.49E+00	0	Single admission	10
2016	2	1.08E+00	1	8.33E-01	1	Repeated admissions	10
2016	0	8.36E-01	1	5.33E+00	1	Single admission	10
2016	1	4.28E+00	0	1.10E+01	0	Single admission	10
2016	7	4.27E+00	0	1.62E+00	0	Single admission	10
2016	4	4.25E+00	0	4.56E+00	0	Single admission	10
2016	10	4.20E+00	0	6.21E+00	0	Single admission	10
2016	2	4.19E+00	0	6.49E+00	0	Single admission	10
2016	1	4.19E+00	0	1.37E+00	0	Single admission	10
2016	4	4.18E+00	0	4.18E+00	0	Single admission	10
2016	1	4.14E+00	0	9.11E-01	0	Single admission	10
2016	1	4.12E+00	0	1.14E+00	0	Repeated admissions	10

Admission year	Age (years)	Follow up time (years)	Sex (Females=1)	PDRLast	Outcome (Deceased=1)	Single/repeated admission	Diagnostic group
2016	1	4.12E+00	0	1.39E+00	0	Single admission	10
2016	13	4.11E+00	1	8.51E-01	0	Single admission	10
2016	1	4.10E+00	0	6.87E+00	0	Single admission	10
2016	3	4.10E+00	0	3.66E+00	0	Single admission	10
2016	1	4.09E+00	1	1.38E+00	0	Single admission	10
2016	8	4.09E+00	0	1.39E+00	0	Repeated admissions	10
2016	13	4.07E+00	0	3.60E+00	0	Single admission	10
2016	2	4.01E+00	1	1.62E+00	0	Single admission	10
2016	0	4.01E+00	1	6.92E+00	0	Single admission	10
2016	5	3.98E+00	1	8.51E-01	0	Repeated admissions	10
2016	1	3.97E+00	0	2.80E+00	0	Repeated admissions	10
2016	17	3.95E+00	0	7.51E-01	0	Single admission	10
2016	14	3.94E+00	1	1.18E+00	0	Single admission	10
2016	0	3.88E+00	1	7.51E-01	0	Single admission	10
2016	4	3.87E+00	0	1.45E+00	0	Single admission	10
2016	0	3.84E+00	0	6.15E+00	0	Single admission	10
2016	15	3.84E+00	0	9.41E+00	0	Single admission	10
2016	0	3.81E+00	1	1.66E+00	0	Single admission	10
2016	7	3.81E+00	1	1.20E+00	0	Single admission	10
2016	11	9.56E-01	1	1.03E+00	1	Single admission	10
2016	1	3.79E+00	1	1.38E+00	0	Single admission	10
2016	1	3.79E+00	0	2.11E+00	0	Single admission	10
2016	6	3.78E+00	0	4.50E+00	0	Single admission	10
2016	0	3.78E+00	0	7.51E-01	0	Single admission	10
2016	16	3.68E+00	0	7.51E-01	0	Single admission	10
2016	7	3.68E+00	0	7.51E-01	0	Single admission	10
2016	5	3.67E+00	0	7.51E-01	0	Single admission	10
2016	1	3.55E+00	0	7.51E-01	0	Single admission	10
2016	0	3.54E+00	0	7.51E-01	0	Single admission	10
2016	8	3.52E+00	0	7.51E-01	0	Single admission	10

Admission year	Age (years)	Follow up time (years)	Sex (Females=1)	PDRLast	Outcome (Deceased=1)	Single/repeated admission	Diagnostic group
2016	12	3.51E+00	1	7.51E-01	0	Single admission	10
2016	4	3.49E+00	1	7.51E-01	0	Single admission	10
2016	0	4.43E+00	1	1.22E+00	0	Single admission	11
2016	0	4.38E+00	0	8.03E+00	0	Single admission	11
2016	0	4.37E+00	1	8.40E+00	0	Single admission	11
2016	0	4.35E+00	0	1.46E+00	0	Single admission	11
2016	0	4.35E+00	1	7.43E+00	0	Single admission	11
2016	0	4.34E+00	1	8.29E+00	0	Repeated admissions	11
2016	0	4.33E+00	0	7.51E-01	0	Repeated admissions	11
2016	0	3.08E+00	1	7.89E+00	0	Single admission	11
2016	0	4.31E+00	1	6.20E+00	0	Single admission	11
2016	0	4.29E+00	1	3.15E+00	0	Single admission	11
2016	0	4.27E+00	1	1.05E+00	0	Single admission	11
2016	0	4.26E+00	0	7.72E-01	0	Repeated admissions	11
2016	0	4.23E+00	0	7.69E+00	0	Single admission	11
2016	0	4.22E+00	0	2.59E+00	0	Single admission	11
2016	0	1.79E-02	0	1.17E+01	1	Single admission	11
2016	0	4.21E+00	0	2.76E+00	0	Single admission	11
2016	0	1.70E-02	1	5.49E+01	1	Single admission	11
2016	0	4.20E+00	0	1.34E+01	0	Single admission	11
2016	0	9.48E-02	0	1.00E+01	1	Repeated admissions	11
2016	0	4.19E+00	0	1.50E+00	0	Single admission	11
2016	2	4.17E+00	0	9.05E+00	0	Single admission	11
2016	11	4.17E+00	1	1.20E+00	0	Repeated admissions	11
2016	0	2.74E-04	0	2.80E+00	1	Single admission	11
2016	0	4.16E+00	1	6.14E+00	0	Repeated admissions	11
2016	0	4.16E+00	1	4.67E+00	0	Single admission	11
2016	0	4.15E+00	0	9.96E+00	0	Single admission	11
2016	0	4.14E+00	0	1.71E+00	0	Single admission	11
2016	0	4.12E+00	0	1.59E+01	0	Single admission	11

Admission year	Age (years)	Follow up time (years)	Sex (Females=1)	PDRLast	Outcome (Deceased=1)	Single/repeated admission	Diagnostic group
2016	0	4.10E+00	1	7.51E-01	0	Repeated admissions	11
2016	0	4.04E+00	0	7.51E-01	0	Repeated admissions	11
2016	0	4.04E+00	0	1.67E+00	0	Single admission	11
2016	1	2.79E-01	1	2.64E+01	1	Repeated admissions	11
2016	0	4.02E+00	0	2.69E+00	0	Single admission	11
2016	0	3.93E+00	0	2.08E+00	0	Single admission	11
2016	0	3.89E+00	0	7.51E-01	0	Single admission	11
2016	0	3.89E+00	1	2.14E+00	0	Single admission	11
2016	0	3.88E+00	0	7.51E-01	0	Single admission	11
2016	0	3.86E+00	0	4.47E+00	0	Single admission	11
2016	0	3.86E+00	1	2.62E+00	0	Single admission	11
2016	0	3.85E+00	1	1.20E+00	0	Single admission	11
2016	0	3.83E+00	1	1.20E+00	0	Single admission	11
2016	0	3.82E+00	0	2.00E+00	0	Single admission	11
2016	1	3.79E+00	0	4.24E+00	0	Single admission	11
2016	0	3.78E+00	1	5.21E+00	0	Single admission	11
2016	0	3.77E+00	1	1.32E+01	0	Single admission	11
2016	0	3.77E+00	1	7.51E-01	0	Repeated admissions	11
2016	0	4.44E-02	0	7.05E+00	1	Repeated admissions	11
2016	12	3.74E+00	0	9.74E-01	0	Single admission	11
2016	0	3.71E+00	1	7.51E-01	0	Single admission	11
2016	1	3.70E+00	0	7.51E-01	0	Single admission	11
2016	0	3.68E+00	1	7.51E-01	0	Single admission	11
2016	0	3.63E+00	0	7.51E-01	0	Single admission	11
2016	0	3.61E+00	1	7.51E-01	0	Single admission	11
2016	0	3.60E+00	0	7.51E-01	0	Single admission	11
2016	0	3.60E+00	1	7.51E-01	0	Single admission	11
2016	0	3.57E+00	1	7.51E-01	0	Single admission	11
2016	0	3.55E+00	0	7.51E-01	0	Single admission	11
2016	0	3.54E+00	0	7.51E-01	0	Single admission	11

Admission year	Age (years)	Follow up time (years)	Sex (Females=1)	PDRLast	Outcome (Deceased=1)	Single/repeated admission	Diagnostic group
2016	0	3.53E+00	0	7.51E-01	0	Single admission	11
2016	0	3.53E+00	0	7.51E-01	0	Repeated admissions	11
2016	0	3.52E+00	0	7.51E-01	0	Single admission	11
2016	0	3.48E+00	0	7.51E-01	0	Single admission	11
2016	1	4.48E+00	0	1.20E+00	0	Single admission	12
2016	3	4.47E+00	1	2.16E-01	0	Single admission	12
2016	0	4.47E+00	0	1.07E-01	0	Repeated admissions	12
2016	12	4.47E+00	1	5.56E+00	0	Single admission	12
2016	0	4.44E+00	0	7.47E+00	0	Single admission	12
2016	0	4.44E+00	1	1.17E+00	0	Single admission	12
2016	5	4.43E+00	0	1.14E+00	0	Single admission	12
2016	0	4.43E+00	0	1.42E+00	0	Single admission	12
2016	1	7.96E-02	1	2.80E+00	1	Single admission	12
2016	7	4.40E+00	0	5.29E+00	0	Single admission	12
2016	3	1.58E+00	0	7.51E-01	1	Repeated admissions	12
2016	1	4.39E+00	0	4.19E+00	0	Single admission	12
2016	0	4.39E+00	1	1.75E+00	0	Single admission	12
2016	2	4.39E+00	1	4.18E+00	0	Single admission	12
2016	0	4.39E+00	0	3.13E+00	0	Single admission	12
2016	0	4.38E+00	1	6.46E-01	0	Single admission	12
2016	0	1.90E-01	1	7.31E+00	1	Single admission	12
2016	0	4.38E+00	0	1.77E+00	0	Single admission	12
2016	3	4.37E+00	1	1.25E+00	0	Repeated admissions	12
2016	2	4.36E+00	0	1.14E+00	0	Single admission	12
2016	0	4.36E+00	1	5.09E+00	0	Single admission	12
2016	0	4.35E+00	0	4.02E+00	0	Single admission	12
2016	3	3.58E+00	1	6.28E+00	0	Single admission	12
2016	2	4.35E+00	1	6.44E+00	0	Single admission	12
2016	0	4.34E+00	1	6.20E+00	0	Single admission	12
2016	8	4.34E+00	0	7.83E-01	0	Single admission	12

Admission year	Age (years)	Follow up time (years)	Sex (Females=1)	PDRLast	Outcome (Deceased=1)	Single/repeated admission	Diagnostic group
2016	7	4.33E+00	1	1.35E+00	0	Single admission	12
2016	4	4.32E+00	0	7.51E-01	0	Single admission	12
2016	1	4.32E+00	0	8.51E-01	0	Single admission	12
2016	2	4.32E+00	1	1.76E+00	0	Single admission	12
2016	1	1.17E+00	0	7.63E+00	1	Single admission	12
2016	2	4.32E+00	0	4.21E+00	0	Repeated admissions	12
2016	1	4.31E+00	0	4.53E+00	0	Single admission	12
2016	0	4.31E+00	0	5.73E-01	0	Repeated admissions	12
2016	1	4.31E+00	1	1.11E+00	0	Single admission	12
2016	0	4.31E+00	1	4.78E+00	0	Single admission	12
2016	0	4.30E+00	0	1.46E+00	0	Single admission	12
2016	0	4.30E+00	0	7.51E-01	0	Single admission	12
2016	0	4.30E+00	0	4.19E+00	0	Single admission	12
2016	0	4.30E+00	1	2.80E+00	0	Single admission	12
2016	1	4.29E+00	1	3.42E+00	0	Single admission	12
2016	0	4.28E+00	0	1.84E+00	0	Single admission	12
2016	1	4.28E+00	1	1.58E+01	0	Single admission	12
2016	6	4.28E+00	1	6.81E+00	0	Repeated admissions	12
2016	3	4.27E+00	0	3.29E+00	0	Single admission	12
2016	0	4.27E+00	0	7.41E+00	0	Single admission	12
2016	1	4.27E+00	0	1.26E+01	0	Single admission	12
2016	1	4.26E+00	1	7.51E-01	0	Repeated admissions	12
2016	1	4.26E+00	0	2.41E+00	0	Single admission	12
2016	3	4.26E+00	1	1.39E+00	0	Single admission	12
2016	0	4.23E+00	1	7.51E-01	0	Single admission	12
2016	0	4.23E+00	1	1.39E+00	0	Repeated admissions	12
2016	1	4.23E+00	1	1.11E+00	0	Single admission	12
2016	0	4.22E+00	1	1.08E+00	0	Single admission	12
2016	4	4.21E+00	0	1.13E+00	0	Single admission	12
2016	0	4.20E+00	1	3.39E+00	0	Single admission	12

Admission year	Age (years)	Follow up time (years)	Sex (Females=1)	PDRLast	Outcome (Deceased=1)	Single/repeated admission	Diagnostic group
2016	0	4.19E+00	0	8.07E+00	0	Single admission	12
2016	15	4.19E+00	0	3.77E-01	0	Single admission	12
2016	10	9.69E-01	1	1.68E+00	1	Single admission	12
2016	0	4.16E+00	0	4.24E+00	0	Single admission	12
2016	0	4.16E+00	0	7.51E-01	0	Single admission	12
2016	0	4.16E+00	1	9.72E-01	0	Single admission	12
2016	0	4.12E+00	0	9.90E-01	0	Single admission	12
2016	0	4.12E+00	0	7.51E-01	0	Repeated admissions	12
2016	17	4.11E+00	0	7.19E+00	0	Single admission	12
2016	12	4.09E+00	0	1.45E+00	0	Single admission	12
2016	2	4.08E+00	0	3.03E+00	0	Single admission	12
2016	0	1.86E+00	1	3.52E+00	1	Repeated admissions	12
2016	0	4.07E+00	1	4.55E+00	0	Single admission	12
2016	5	4.06E+00	0	4.41E+00	0	Single admission	12
2016	7	4.04E+00	0	1.23E+00	0	Single admission	12
2016	0	4.03E+00	1	8.33E-01	0	Single admission	12
2016	0	4.03E+00	0	9.49E-01	0	Single admission	12
2016	0	4.01E+00	0	2.80E+00	0	Single admission	12
2016	0	3.40E+00	0	7.51E-01	0	Single admission	12
2016	2	4.00E+00	1	1.33E+00	0	Single admission	12
2016	1	3.99E+00	1	5.25E+00	0	Single admission	12
2016	1	3.99E+00	1	5.25E+00	0	Single admission	12
2016	0	3.99E+00	1	5.35E+00	0	Single admission	12
2016	0	3.97E+00	1	5.57E-01	0	Repeated admissions	12
2016	1	3.97E+00	1	6.22E+00	0	Single admission	12
2016	1	3.96E+00	0	1.14E+00	0	Repeated admissions	12
2016	1	6.60E-01	0	9.61E+00	1	Single admission	12
2016	0	3.88E+00	0	1.23E+00	0	Single admission	12
2016	1	3.81E+00	1	7.51E-01	0	Repeated admissions	12
2016	1	3.81E+00	0	8.69E-01	0	Single admission	12

Admission year	Age (years)	Follow up time (years)	Sex (Females=1)	PDRLast	Outcome (Deceased=1)	Single/repeated admission	Diagnostic group
2016	0	3.80E+00	0	1.15E+00	0	Repeated admissions	12
2016	2	3.80E+00	1	4.06E+00	0	Single admission	12
2016	1	3.79E+00	0	7.51E-01	0	Single admission	12
2016	0	3.79E+00	1	4.54E+00	0	Single admission	12
2016	8	3.78E+00	0	1.26E+00	0	Single admission	12
2016	1	3.74E+00	1	1.14E+00	0	Single admission	12
2016	0	3.73E+00	0	5.20E-01	0	Single admission	12
2016	1	3.70E+00	1	7.51E-01	0	Single admission	12
2016	1	3.68E+00	0	7.51E-01	0	Single admission	12
2016	6	3.66E+00	1	7.51E-01	0	Single admission	12
2016	1	3.65E+00	0	7.51E-01	0	Single admission	12
2016	0	3.64E+00	1	7.51E-01	0	Repeated admissions	12
2016	0	3.63E+00	1	7.51E-01	0	Repeated admissions	12
2016	2	3.62E+00	1	7.51E-01	0	Single admission	12
2016	1	3.62E+00	0	7.51E-01	0	Single admission	12
2016	1	3.57E+00	1	7.51E-01	0	Single admission	12
2016	0	3.56E+00	0	7.51E-01	0	Single admission	12
2016	0	3.55E+00	0	7.51E-01	0	Single admission	12
2016	2	3.55E+00	1	7.51E-01	0	Single admission	12
2016	0	3.54E+00	0	7.51E-01	0	Single admission	12
2016	6	3.53E+00	1	7.51E-01	0	Repeated admissions	12
2016	0	3.53E+00	0	7.51E-01	0	Single admission	12
2016	1	3.53E+00	0	7.51E-01	0	Single admission	12
2016	0	3.51E+00	0	7.51E-01	0	Single admission	12
2016	0	3.51E+00	0	7.51E-01	0	Single admission	12
2016	1	3.51E+00	0	7.51E-01	0	Single admission	12
2016	0	3.51E+00	1	7.51E-01	0	Single admission	12
2016	0	3.50E+00	1	7.51E-01	0	Single admission	12
2016	0	3.50E+00	0	7.51E-01	0	Single admission	12
2016	0	3.50E+00	0	7.51E-01	0	Single admission	12

Admission year	Age (years)	Follow up time (years)	Sex (Females=1)	PDRLast	Outcome (Deceased=1)	Single/repeated admission	Diagnostic group
2016	2	3.50E+00	1	7.51E-01	0	Single admission	12
2016	0	3.59E+00	0	7.51E-01	0	Single admission	13
2016	5	3.54E+00	0	7.51E-01	0	Single admission	13
2016	15	3.54E+00	0	7.51E-01	0	Single admission	13

Diagnostic groups

1	Nephrology
2	Certain perinatal conditions
3	Gastrointestinal including hepatic failure
4	Infection including septic shock + systemic inflammatory response syndrome
5	Haematology and oncology
6	Cardiovascular/Circulatory
7	Endocrine and metabolic diseases
8	Trauma and poison
9	Observations, postoperative or symptom based
10	Neurological including convulsions
11	Malformations
12	Respiratory
13	Unknown
