# Supplementary material for: Risks for death after admission to pediatric intensive care (PICU)—A comparison with the general population
Source: PLoS One. 2022 Oct 7;17(10):e0265792. doi: 10.1371/journal.pone.0265792 (PMC9543762; doi:10.1371/journal.pone.0265792)
Supplement: S2 Table — The hierarchical nature of patients with repeated admissions to PICU. (PDF) [file pone.0265792.s002.pdf]

Suppl. Table S2 Number of repeated admissions

Number of repeated admissions	Number or patients
15	2
14	1
13	2
12	2
11	1
10	6
9	6
8	8
7	13
6	25
5	49
4	80
3	179
2	555
