# Supplementary material for: Risks for death after admission to pediatric intensive care (PICU)—A comparison with the general population
Source: PLoS One. 2022 Oct 7;17(10):e0265792. doi: 10.1371/journal.pone.0265792 (PMC9543762; doi:10.1371/journal.pone.0265792)
Supplement: S3 Table — The number of patients at follow-up years 0 to 12 for the entire study cohort. (PDF) [file pone.0265792.s003.pdf]

Suppl. Table S3

Time (years)	Patients at risk			
	Single admissions		Repeated admissions	
	Females	Males	Females	Males
0	1578	2175	405	524
1	1493	2069	363	475
2	1481	2056	346	452
3	1473	2042	336	444
4	1390	1937	318	432
5	1230	1699	282	393
6	1059	1437	247	340
7	863	1219	216	294
8	697	1010	172	251
9	533	811	141	206
10	391	574	109	155
11	251	343	59	88
12	89	105	21	22

Data from Figure 2
