# Supplementary material for: Risks for death after admission to pediatric intensive care (PICU)—A comparison with the general population
Source: PLoS One. 2022 Oct 7;17(10):e0265792. doi: 10.1371/journal.pone.0265792 (PMC9543762; doi:10.1371/journal.pone.0265792)
Supplement: Attachment — Submitted filename: Responses to Reviewers.docx [file pone.0265792.s005.docx]

Responses to Reviewers' Comments to Author,

Thank you for Your constructive and thoughtful comments and suggestions. We are grateful for the opportunity to revise our article and agree that the manuscript can be much improved by changes according to your suggestions.

Below written in red, are our responses to Your suggestions and comments.

Reviewer #2: Thank you for providing the supplementary material. The responses, along with the additional tables in the supplements, addressed most of my previous concerns.

I have a remaining comment/question regarding Figure 3. In my previous review, I had asked about the non-zero SMR at year=0 (time of PICU admission). The response did not really clear up my original question.

In Figure 3, there is a certain "binning" taking place for the number of years on the horizontal axis, i.e. data are plotted only for discrete years 0, 1, 2, 3, 4, and 5 after PICU admission. When you plot the SMR for say year=0, are you counting the observed deaths up to, but strictly less than, 1 year from PICU admission? Likewise, does the SMR for year=1 reflect observed deaths that occurred at least 1 year, but strictly less than 2 years, after PICU admission?

Reply: We thank the reviewer for pointing out this important weakness. We are grateful for the opportunity to make clarifications and have now made changes accordingly in Figure 3 on the horizontal axis and the accompanying Figure legend. The plotted years on the horizontal axis are showing intervals, i.e., SMR for year 0= from PICU admission – end of observation period (up to 12.5 years), year 1= one year after PICU admission – end of observation period, year 2= two years after PICU admission – end of observation period etc. Figure 3 and Figure legend.

This revised statement -- "The SMRs, during the total follow-up time for the entire PICU patient population with single and repeated admissions was 35.2% ... and 108.0 ...." (lines 176-179, page 14) -- where the stated numbers correspond to the year=0 point on the horizontal axis, still does not track with my understanding of what Figure 3 is supposed to be showing. How does "total follow-up time" track with year=0?

Reply: We appreciate this comment, to clarify we have made changes in the manuscript accordingly, lines 168-186, page 14.

Figure 3, especially the number corresponding to year=0 for repeated admissions, is referenced in the Discussion section, so please make sure to explain clearly/thoroughly what year=0 (after PICU admission) really means.

Reply: We thank the reviewer for pointing this out to us. We have revised the text accordingly, lines 243-245 and line 251 page 17.
